# Redundancy maximization as a principle of associative memory learning in Hopfield networks

**DOI:** 10.1038/s41467-026-78777-2

**Published:** 2026-11-06

**Authors:** Mark Blümel, Andreas C. Schneider, Valentin Neuhaus, David A. Ehrlich, Marcel Graetz, Michael Wibral, Abdullah Makkeh, Viola Priesemann

**Affiliations:** 1 Complex Systems Theory, Max Planck Institute for Dynamics and Self-Organization, Göttingen, Germany; 2 Faculty of Physics, Institute for the Dynamics of Complex Systems, University of Göttingen, Göttingen, Germany; 3 Göttingen Campus Institute for Dynamics of Biological Networks, University of Göttingen, Göttingen, Germany; 4 Champalimaud Centre for the Unknown, Lisbon, Portugal

**Keywords:** Computer science, Information theory and computation, Learning algorithms

## Abstract

Associative memory, traditionally modeled by Hopfield networks, enables the retrieval of previously stored patterns from partial or noisy cues. Yet, the local computational principles which are required to enable this function remain incompletely understood. To formally characterize the local information processing in such systems, we employ a recent extension of information theory—Partial Information Decomposition. It decomposes the contribution of different inputs to an output into unique information from each input, redundant information across inputs, and synergistic information that emerges from combining different inputs. Applying this framework to individual neurons in classical Hopfield networks we find that below the memory capacity, the information in a neuron’s activity is characterized by high redundancy between the external pattern input and the internal recurrent input, while synergy and unique information are close to zero until the memory capacity is surpassed and performance drops steeply. Inspired by this observation, we use redundancy maximization at each neuron as an information-theoretic learning goal. This increases the network’s memory capacity to 1.59, a more than tenfold improvement over the 0.14 capacity of classical Hopfield networks, and also outperforming recent state-of-the-art implementations of Hopfield networks. Overall, this work establishes redundancy maximization as a design principle for associative memories and opens pathways for new associative memory models based on information-theoretic goals.

## Abstract (web summary)

Local computational principles for modeling associative memory remain incompletely understood. The authors show that high local redundant information between the inputs of each neuron is a key principle of associative memory learning in Hopfield networks, and that direct maximization of this redundancy results in a new state-of-the-art learning rule.

## Introduction

Associative memory—the ability to retrieve patterns from noisy or partial inputs—is a fundamental brain function, enabling the retrieval of memories from imperfect sensory stimuli. This type of content-addressable memory can be modeled by recurrent neural networks called “Hopfield networks”, for which their inventor John Hopfield was recognized with the Nobel Prize in physics in 2024^1,2^. Recently, continuous-valued extensions of Hopfield networks have also found renewed application in machine learning^3^.

Despite decades of development since their first introduction in 1982, the principles underlying associative memory formation remain incompletely understood. Originally, Hopfield networks were trained using the biologically-inspired Hebbian learning rule based on firing coincidences. Since then, new learning rules have been introduced that display improved memory capacity and stability^4,5^. Nevertheless, a key question remains: Is there an underlying principle that governs the formation of associative memory? And, if so, can it be exploited directly to improve performance?

To answer these questions, we propose to analyze Hopfield networks from an information processing perspective. Hopfield networks store patterns as attractors of their neural dynamics, created by training the network’s weights using the patterns as a teaching signal. How the information of the recurrent dynamics and the teaching signal together predicts the neuron’s firing thus becomes pivotal to the network’s performance. Describing this relation in an abstract, implementation-independent manner can be achieved using the framework of information theory^6,7^. In particular, the mutual information quantifies general statistical dependencies between two variables, enabling the quantification of the amount of information in a neuron’s output that is contained in the input from other neurons (recurrent input) or in the teaching signal (target input). However, using only mutual information it is impossible to differentiate how that information is contributed in unique, redundant, or synergistic ways. We can differentiate these input contributions using a recent extension of information theory called Partial Information Decomposition (PID, ref. ^8^, see Fig. 2c).

In this work, we use PID as a tool to describe the individual neuron’s function in a Hopfield network in both an analytic and constructive manner. The analysis of classical Hebbian Hopfield networks reveals that neurons show high redundancy in their firing between recurrent and teaching inputs. Building on this insight, we then use PID to construct local goal functions which optimize for redundancy and other information processing goals directly.

Information theory and the concept of PID have been used to analyze information processing in artificial neural networks in the past^9–12^. Furthermore, it has been shown that infomorphic neurons which train by directly optimizing a PID goal function are in principle capable of solving tasks from different learning paradigms^13^ and have been demonstrated to achieve high performance and local interpretability for supervised learning tasks in particular^14^.

The main contributions of this work are (i) an information-theoretic analysis of a classical Hebbian Hopfield network which reveals that redundancy between recurrent input and target (teaching signal) dominates the neurons’ output when operating below capacity, (ii) the construction of infomorphic associative memory neurons that directly maximize a local PID goal function, building on and improving the proof of concept provided by Makkeh et al.^13^, and (iii) experimental results showcasing that infomorphic neurons that maximize redundancy significantly outperform the original Hebbian learning rule and other previous learning methods.

## Results

**Fig. 1:**
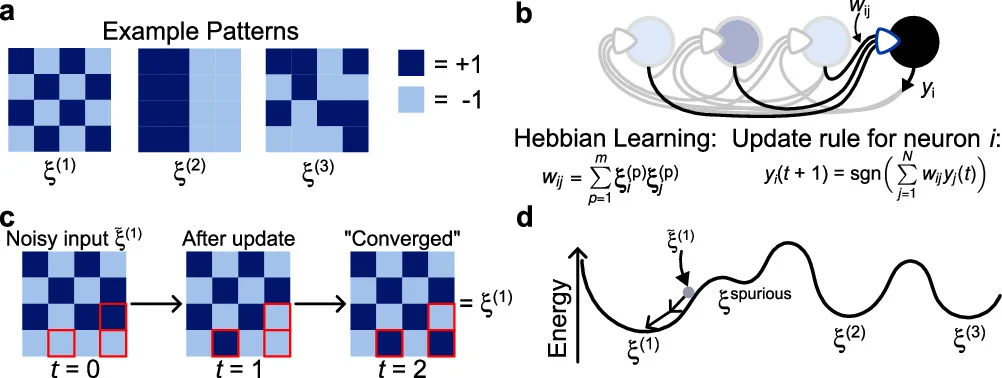
Hopfield networks store patterns as attractors and retrieve them by iteratively updating noisy inputs. **a** Three example patterns that could be stored in a Hopfield network (vectors *ξ*^(*p*)^ re-formatted to 2-D for visual clarity). Based on the patterns, the weights of the network (**b**) can be directly computed. **c** The neurons are initialized in a state that is a corrupted version of a pattern (here *ξ*^(1)^ with corruptions marked in red) and the network updates its states based on the given update rule. If successful, the network converges to the original pattern. **d** The stored patterns *ξ*^(*p*)^ become minima in the energy landscape of the network. If the model starts close to a stored pattern, it will converge back to its minimum energy state and recover the pattern. However, if the capacity of the network is exceeded, i.e., the minima become too close in the energy landscape, new local minima can form, resulting in so-called spurious memories. This energy landscape view holds only for symmetric Hopfield networks (i.e., where *w*_*ij*_ = *w*_*ji*_).

A Hopfield network consists of *N* recurrently connected neurons. Initialized with a noisy or incomplete version of a stored pattern, the network iteratively updates its neuron states, converging to a stable state that corresponds to the stored pattern and thereby completing its pattern and correcting its errors (see Fig. 1). For uncorrelated patterns, the number of patterns that can be stored this way grows linearly with network size, so one defines the memory load $\alpha=\frac{m}{N}$ for *m* patterns in *N* neurons. The largest memory load at which all patterns are still recalled defines the memory capacity *α*_*c*_, which for the classical Hebbian learning reaches *α*_H_ ≈ 0.14 ^15^.

### Redundancy in Hebbian learning

**Fig. 2:**
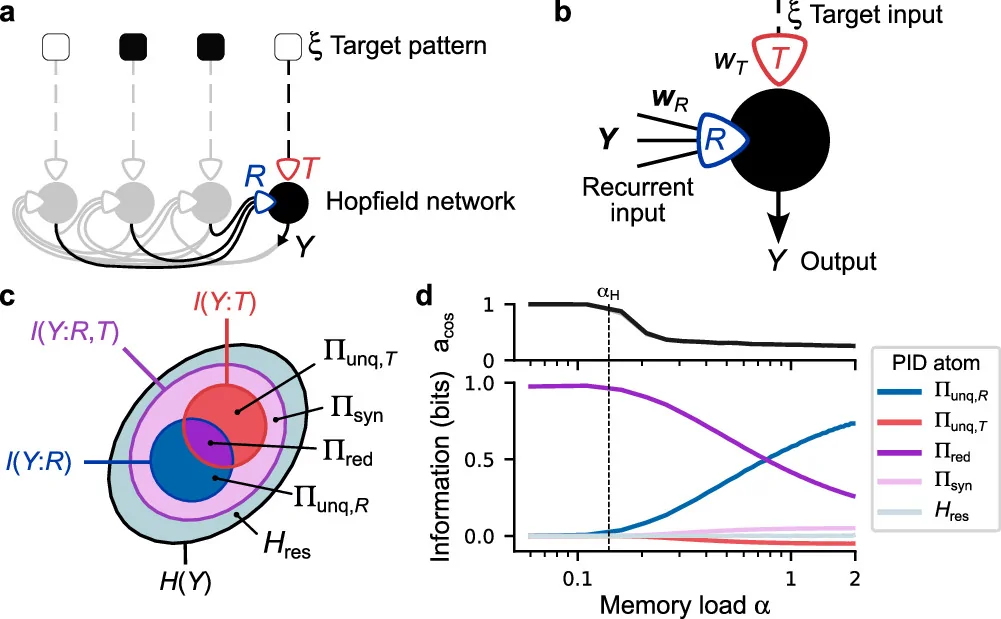
For classical Hopfield networks trained with Hebbian learning, redundant information between target and recurrent input coincides with successful memory storage. **a** Schematic of the analysis set-up for Hopfield networks. To measure how information is represented, each neuron is compared to a non-driving target input *T* that provides the ground-truth pattern, in addition to its recurrent input *R*. **b** Each neuron in the Hopfield network aggregates its recurrent inputs and produces an output *Y*. The neurons are initialized in the target state *ξ*. **c** Partial information decomposition (PID) separates the entropy of the output *Y* into five parts: Unique information (provided by only one of the two inputs), redundant (shared by both inputs), synergistic (emerging only from the combination of inputs) and residual entropy (not explained by the inputs). **d** The PID profile as a function of memory load *α*. Below the network’s memory capacity (*α*_H_ ≈ 0.14, indicated by dashed black line), the redundancy Π_red_ is high. Above capacity, as recall fails, redundancy collapses and is replaced by unique information from the recurrent input. The accuracy of recall is shown in black. The PID profiles show the median of 20 network initializations, with values first averaged across all neurons. The accuracy curve is the median of the 20 initializations. Shaded areas indicate the central 90 % percentile of values. Results are from a network with *N* = 500 neurons to minimize finite-size effects (see Supplementary Note 5.2). A more technical and analytical discussion of the results, including a reproduction of panel d using other PID measures, can be found in Supplementary Note 6.

To understand the information-theoretic footprint of Hebbian Hopfield networks, we first investigate a network with 500 neurons trained using Hebbian learning. Alongside the recurrent input *R* that each neuron receives, we introduce the corresponding target input *T* that carries the ground truth pattern but does not drive the neuron (see Fig. 2a, b). Both inputs are used to decompose the output’s entropy into the different PID atoms (see Section “Methods”). Computing the PID across memory loads *α* reveals a systematic shift in the composition of the neurons’ output: for memory loads below and close to the memory capacity of *α*_H_ ≈ 0.14, redundant information is the largest information contribution (see Fig. 2d), indicating that high redundant information Π_red_ coincides with successful learning.

Above the memory capacity, redundant information begins to fall while the unique information Π_unq,R_ begins to rise, indicating that the neurons start to encode more non-relevant information from the recurrent inputs. In Supplementary Note 6 we provide a formal connection between high redundancy and high recall accuracy in Hebbian Hopfield networks as well as empirical results using other PID measures.

Overall, the results suggest that high redundant information may play an important role in successful associative memory function.

### Redundancy as a computational goal

To evaluate whether the information-theoretic description found in subsection “Redundancy in Hebbian learning” is sufficient to describe associative memory function, this section introduces a constructive approach to show how maximization of redundancy can be used as a computational goal for associative memory learning and shows that it outperforms the classical Hopfield network.

**Fig. 3:**
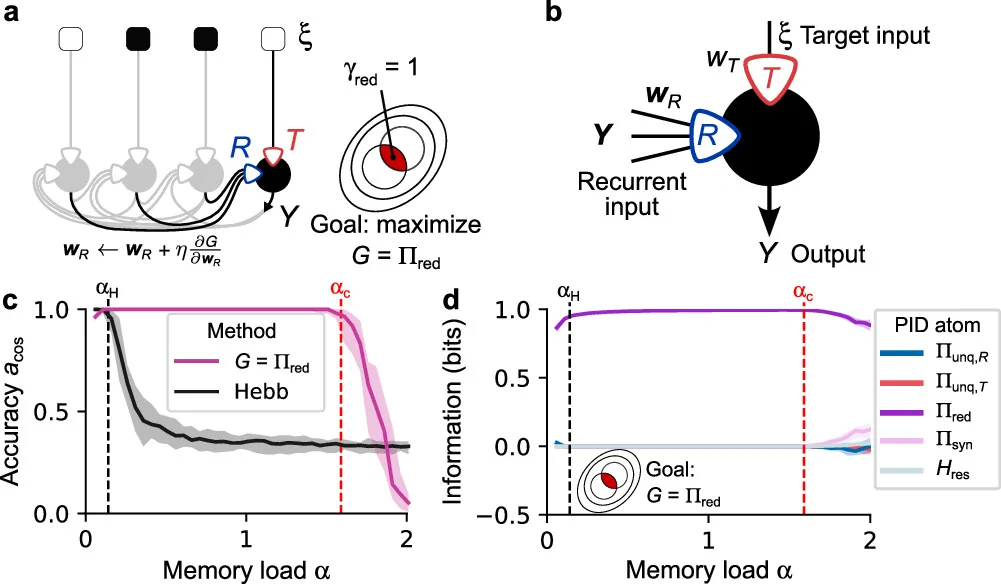
Redundancy maximization between recurrent connections and a target is a sufficient principle for memorization in Hopfield networks, achieving a memory capacity of ${\alpha }_{{{{\rm{c}}}}}^{{\rm{red}}}\approx 1.59$. **a** Schematic of the infomorphic Hopfield model. During training of the infomorphic Hopfield network, the recurrent connections ***w***_*R*_ are updated using gradient ascent on the goal function *G* = Π_red_. **b** Each neuron in the infomorphic Hopfield network aggregates its input into two compartments—the recurrent input *R* and the target input *T*. Based on this, they stochastically produce an output *Y*. **c** Recall accuracy as a function of memory load *α* for a network with 100 neurons. Redundancy maximization achieves a memory capacity of ${\alpha }_{{{{\rm{c}}}}}^{{\rm{red}}}=1.59\,[1.56,1.61]$, far exceeding the Hebbian capacity of *α*_H_ ≈ 0.14 (capacities marked by dashed lines). **d** The PID profile shows the mean information atoms per neuron as a function of memory load *α*. Redundancy dominates below capacity $\alpha < {\alpha }_{{{{\rm{c}}}}}^{{\rm{red}}}$, then falls off as the capacity is crossed and the other atoms become non-zero. In (**c**), the curve shows the median calculated across 20 network initializations. In (**d**), values are first averaged across all neurons, and the curve then shows the median of these averages across the initializations. For both panels, the shaded area represents the central 90% of the data, spanning the 5th to the 95th percentile.

To this end, we employ infomorphic neurons, which integrate their inputs into two separate compartments (the recurrent input *R* and the target input *T*) and update their recurrent weights by gradient ascent on a PID-based goal function (Fig. 3a, b; subsection “Information-theoretic learning” and Supplementary Note 1.3). The target input is only used during training and omitted during testing. At test time, the network relaxes on its recurrent input alone under the same update rule as the classical Hopfield network. Motivated by the results of the previous section, we first use the simple neuron goal function *G* = Π_red_, i.e., we train each neuron to maximize the redundant information between its inputs about the output.

To evaluate the performance of these infomorphic Hopfield networks, we train networks with *N* = 100 neurons for different numbers of patterns and test them by initializing them in a stored pattern and running them until convergence (see also Supplementary Note 1.4). Recall is then scored by the cosine similarity between the converged state and the stored pattern, averaged over all patterns. Memory capacity *α*_*c*_ is defined as the largest memory load below which this accuracy remains above 0.95. We find that the network memorizes all patterns up to a memory load of ${\alpha }_{{{{\rm{c}}}}}^{{\rm{red}}}=1.59\,[1.56,1.61]$ followed by a sharp drop in recall accuracy above this memory capacity (see Fig. 3c). The square brackets indicate bootstrapped 95% confidence intervals of the capacity *α*_c_ across 20 network initializations.

The transition above ${\alpha }_{{{{\rm{c}}}}}^{{\rm{red}}}$ can be explained by performing the same PID analysis as in subsection “Redundancy in Hebbian learning”. As shown in Fig. 3d, redundancy is uniformly high below ${\alpha }_{{{{\rm{c}}}}}^{\mathrm{red}}$ apart from some finite size effects for low numbers of patterns discussed in Supplementary Note 5.2. However, when the threshold is crossed, the information encoded by the neurons is no longer purely redundant. Instead, as the neurons fail to encode only task-relevant redundant information, small amounts of other information atoms, particularly synergistic information Π_syn_, start to arise.

### Mutual information goals

**Fig. 4:**
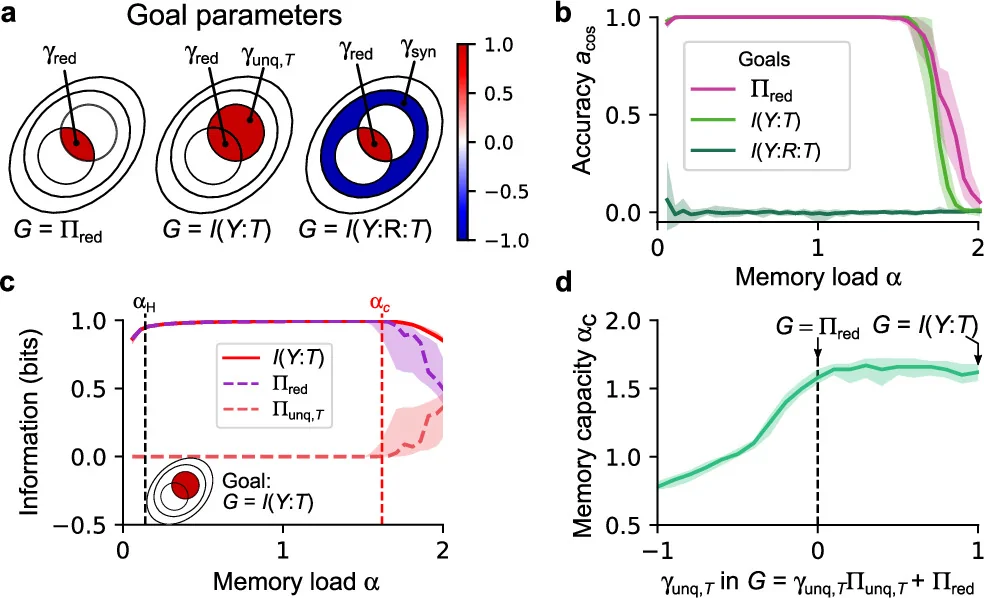
Infomorphic Hopfield networks trained with a classical mutual information goal achieve high memory capacity by implicitly maximizing redundancy. **a** The redundancy goal and two alternative learning objectives based on classical information theory: Maximizing the mutual information between output and target, *G* = *I*(*Y*: *T*), and maximizing the co-information, *G* = *I*(*Y*: *R*: *T*). **b** A performance comparison of the two alternative goals as a function of memory load shows that maximizing mutual information (*G* = *I*(*Y*: *T*)) achieves a very similar capacity as maximizing redundancy (*G* = Π_red_) alone. In contrast, maximizing co-information (*G* = *I*(*Y*: *R*: *T*)) fails to store any patterns. **c** Information profiles for the successful *G* = *I*(*Y*: *T*) goal. Beyond its memory capacity *α*_c_, mutual information *I*(*Y*: *T*) stays high, but redundancy (Π_red_) falls and is replaced by unique information from the target (Π_unq,*T*_). **d** The memory capacity *α*_c_ is shown as a function of *γ*_unq,*T*_ in the goal *G* = *γ*_unq,*T*_Π_unq,*T*_ + Π_red_. While positive values of *γ*_unq,*T*_ have no strong effect on the memory capacity, negative values are detrimental. The curves in (**b**, **d**) show the median across 20 network initializations while in (**c**), values are first averaged across all neurons. In all panels, the shaded area represents the central 90% of the data.

Besides optimizing redundancy or other PID-based goal functions directly, the infomorphic approach also allows us to compare the former to goal functions based on classical information theory alone. To this end, we consider two main candidate goal functions (Fig. 4a), with two further variants discussed below: (i) the mutual information between the target inputs and the neuron’s output *I*(*Y*: *T*) and (ii) the co-information, *I*(*Y*: *R*: *T*), which is sometimes used as a classical measure of the tradeoff between redundancy and synergy^16^. Co-information is formally defined as

1 $$I(Y:R:T)=	 \, I(Y:R)+I(Y:T)-I(Y:R,T)\\=	 \, {{\Pi }}_{\mathrm{red}}-{{\Pi }}_{\mathrm{syn}}.$$

As before, we first evaluate the capacity of the networks trained with the two classical goal functions (Fig. 4b). While maximizing co-information fails to store any patterns, maximizing target mutual information *I*(*Y*: *T*) achieves very similar performance to maximizing redundancy and reaches a memory capacity of *α*_c_ = 1.62 [1.61, 1.64]. In addition, the joint mutual information *I*(*Y*: *T*, *R*) reaches a capacity of *α*_c_ = 0.85 [0.84, 0.86], while the recurrent mutual information *I*(*Y*: *R*) fails to learn any patterns.

The high performance of the mutual information optimization can be understood by looking at the PID profile shown in Fig. 4: The mutual information *I*(*Y*: *T*) can be decomposed into Π_red_ and Π_unq,*T*_, i.e., into a redundant and a unique contribution. Below the maximum capacity *α*_c_, the mutual information is maximized only by maximizing the redundant information Π_red_ between the two inputs, explaining the similar performance to optimizing this redundancy alone. Above that point, the mutual information remains high, but redundancy decreases, matched by an increase in unique information of the target input Π_unq,*T*_. The corresponding decrease in accuracy suggests that the mutual information goal works only as long as it results in a maximization of redundancy, further suggesting redundancy as the underlying principle of Hopfield memory function. The effect of varying the unique contribution *γ*_unq,*T*_ in the goal function *G* = *γ*_unq,*T*_Π_unq,*T*_ + Π_red_ indicates that additional maximization of target unique information hardly impacts the memory capacity while negative values lead to a decrease in performance (see Fig. 4d).

### Improving the capacity of the infomorphic goal

**Fig. 5:**
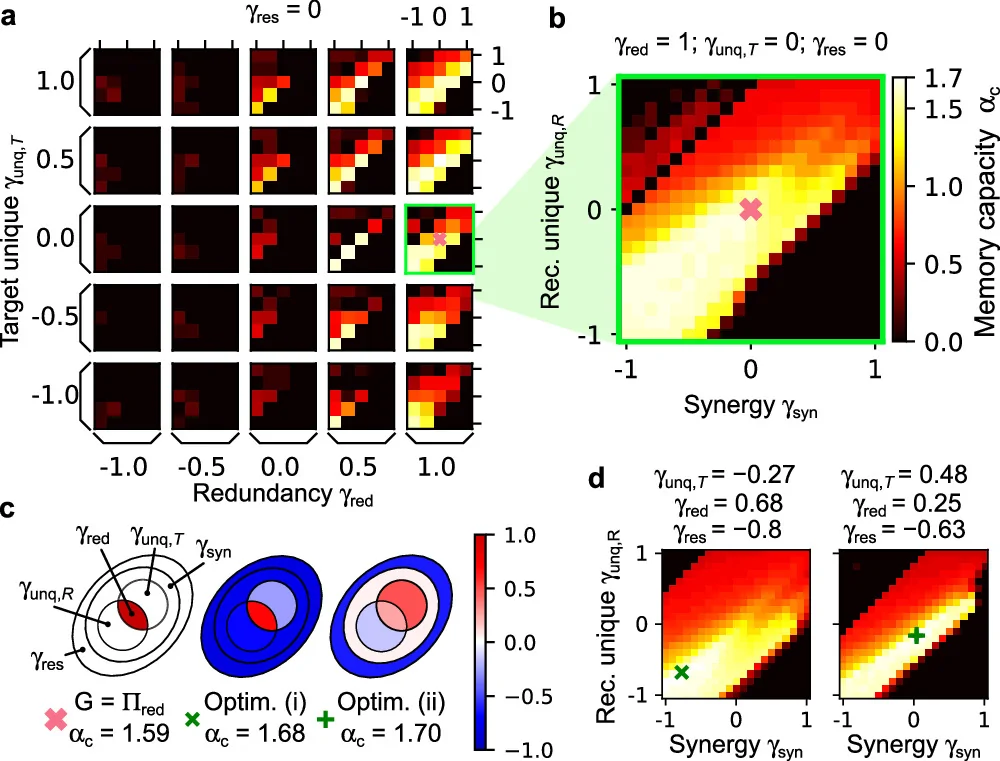
Hyperparameter optimization reveals composite information goals which outperform redundancy maximization. **a** The memory capacity landscape *α*_c_ as a function of the goal parameters *γ*_*i*_ reveals the performance across large parts of the parameter space. The goal parameters for redundancy *γ*_red_ and target unique information *γ*_unq,*T*_ vary on the outer axes, while the goal parameters for synergy *γ*_syn_ and recurrent unique information *γ*_unq,*R*_ vary on the inner axes. The remaining goal parameter for residual entropy *γ*_res_ is fixed at 0. **b** A more detailed subspace of a reveals the area around the heuristic goal. Here, *γ*_unq,*T*_ = 0 and *γ*_red_ = 1 while *γ*_unq,*R*_ and *γ*_syn_ are varied. Suppressing both Π_unq,*R*_ and Π_syn_ slightly improves the capacity above redundancy maximization. **c** A direct optimization of the capacity on the full goal space reveals new goals. The heuristic redundancy goal as well as two of the best performing goals are illustrated. The optimized goals reach a memory capacity of ${\alpha }_{{{{\rm{c}}}}}^{{{{\rm{(i)}}}}}=1.68\,[1.65,1.72]$ and ${\alpha }_{{{{\rm{c}}}}}^{{{{\rm{(ii)}}}}}=1.70\,[1.66,1.71]$. The exact goal parameters and the results of the optimizations are listed in Supplementary Table 2. **d** As in (**b**), the panel shows two slices of the landscape around the optimized goals. The landscapes show a similar structure with distinct local optima.

While optimizing redundancy has proven sufficient for achieving high associative memory performance, it remains open whether this simple intuitive goal is optimal or whether admixtures of other PID atoms can improve performance even further. To investigate this, we performed a parameter sweep over the *γ* parameters in the general goal function *G* = *γ*_unq,*R*_Π_unq,*R*_ + *γ*_unq,*T*_Π_unq,*T*_ + *γ*_red_Π_red_ + *γ*_syn_Π_syn_, with a fixed parameter for residual entropy *γ*_res_ = 0. We then measured the network capacities for the different optimization goals (see Fig. 5).

The resulting performance landscape reveals that the highest capacities are indeed achieved in proximity of the heuristic redundancy goal *G* = Π_red_ (Fig. 5a), consistent with the intuition that in order to memorize a pattern, each neuron’s recurrent activation must align with its target activation. The performance remains mostly stable for positive additions of Π_unq,*T*_, as long as the redundant contribution dominates. Suppressing the target-unrelated information of each neuron by choosing negative parameters for both recurrent unique information and synergy, *γ*_unq,*R*_ and *γ*_*syn*_, appears to slightly increase performance, but only if both parameters are approximately equal (Fig. 5b). In line with this result, the capacity decreases when either or both of them are positive.

Similar to the approach of Schneider et al.^14^, we further used a hyperparameter optimization technique to find optimal hyperparameter combinations. We used the CMA-ES sampler^17^ to find the goal parameters that maximize the capacity of the model. Two of the best goals found with this approach reach capacities of ${\alpha }_{{{{\rm{c}}}}}^{{{{\rm{(i)}}}}}=1.68\,[1.65,1.72]$ and ${\alpha }_{{{{\rm{c}}}}}^{{{{\rm{(ii)}}}}}=1.70\,[1.66,1.71]$, slightly outperforming the heuristic redundancy maximization (${\alpha }_{{{{\rm{c}}}}}^{{\rm{red}}}=1.59\,[1.56,1.61]$) (see Fig. 5c). The two goal functions are

2 $${G}^{{\rm{(i)}}}=	 -0.27\,{{\Pi }}_{{{{\rm{unq}}}},T}-0.68\,{{\Pi }}_{{{{\rm{unq}}}},R}+0.68\,{{\Pi }}_{{{{\rm{red}}}}}-0.77\,{{\Pi }}_{{{{\rm{syn}}}}}-0.80\,{H}_{{{{\rm{res}}}}}\,{{\mbox{and}}}\,\\ {G}^{{\rm{(ii)}}}=	 0.48\,{{\Pi }}_{{{{\rm{unq}}}},T}-0.16\,{{\Pi }}_{{{{\rm{unq}}}},R}+0.25\,{{\Pi }}_{{{{\rm{red}}}}}+0.04\,{{\Pi }}_{{{{\rm{syn}}}}}-0.63\,{H}_{{{{\rm{res}}}}}.$$

While both goals maximize redundancy, the first suppresses all other contributions and the second only suppresses Π_unq,*R*_ and *H*_res_ while it maximizes Π_unq,*T*_, similar to the mutual information goal *I*(*Y*: *T*). The results of the other optimizations can be found in Supplementary Note 3. The landscapes around the optimized goals have a similar qualitative structure in the subspace of *γ*_unq,*R*_ and *γ*_red_, while the optima are at different values (see Fig. 5d).

### Performance comparison

**Fig. 6:**
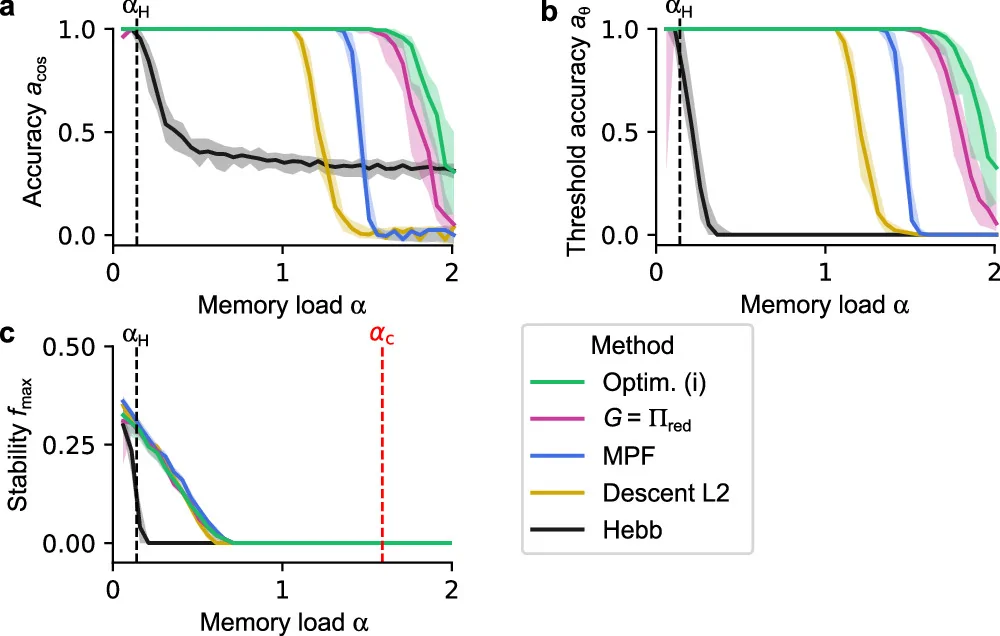
In terms of memory capacity, the infomorphic approach outperforms other high-performance learning methods. **a** Comparison of the performance of the Hebbian learning rule, our heuristic and optimized infomorphic goals, and two high-performance methods, the descent L2 method^5,18^ and the minimum probability flow (MPF)^4^. Using cosine similarity (${a}_{\cos }$) as the accuracy metric, both infomorphic goals outperform all other methods. Above capacity, Hebbian learning displays spurious memories^34^. **b** Same as in (**a**), but accuracy is measured using a strict threshold (*a*_*θ*_ with *θ* = 0.95). This metric reveals that the Hebbian learning rule is unable to fully reconstruct patterns above its capacity *α* > *α*_H_ as its accuracy falls to zero. **c** The stability of memory retrieval is shown as a function of memory load *α*. The figure is adapted from Tolmachev and Manton^5^, with us adding MPF and the infomorphic goals. The infomorphic method is on par with the other goals, although it was not even designed to optimize stability. The curves in (**a**–**c**) show the median values across 20 network initializations, and the shaded areas denote the corresponding central 90% percentile.

Infomorphic Hopfield networks achieve competitive performance compared to other state-of-the-art learning rules for Hopfield networks. To demonstrate this, we selected two learning methods which are also based on goal functions—the minimum probability flow goal (MPF)^4^ and descending the L2 norm (Descent L2)^5,18^. Initially introduced by Sohl-Dickstein et al.^19^, MPF demonstrates that by minimizing the probability of entering neighboring states of the pattern states *ξ*, many patterns can be encoded in a robust way. Our approach differs from MPF by introducing local goal functions at the level of each neuron and by explicitly connecting the probabilistic approach to information theory. Tolmachev and Manton^5^ demonstrate that learning in a Hopfield network can often be implemented as descent of different loss functions that describe the stability of the training patterns. They compare different norms, revealing that minimizing the L2 norm between the pattern and the next evolved time step achieves high memory capacity. Both MPF and Descent L2 reach state-of-the-art capacities above the Hebbian learning rule, but are outperformed by the infomorphic redundancy maximization (see Fig. 6a, b).

In addition, we also evaluate the stability of the memories encoded in different networks (Fig. 6c). The stability is measured by introducing progressively more noise *f* into the patterns and testing whether recall of memories is still successful. The area under the curve ${f}_{\max }$ is a proxy for the overall stability of memories and indicates the point up to which 95 % of the pattern elements are reconstructed. For more details, see Supplementary Note 1.3. The redundancy goal’s profile for stability follows an overall trajectory similar to that of the other learning methods. Note that above *α* ≈ 0.8 stability is reduced to almost zero for all methods.

## Discussion

This work presents a new perspective on associative memories by examining the information-processing at the scale of individual neurons. Using PID, we quantify how recurrent and target inputs predict neuronal output. We demonstrate that effective pattern storage requires the recurrent and target inputs to redundantly determine firing, highlighting redundancy as a key local information-processing principle underlying associative memory function. Analyzing classical Hopfield networks (subsection “Redundancy in Hebbian learning”), we find that below the memory capacity, neurons predominantly process information redundantly, while above capacity, redundancy degrades and unique recurrent contributions increase, indicating a loss of task-relevant encoding (Fig. 2d).

This connection is not coincidental: as we show analytically in Supplementary Note 6, when using a Heaviside activation function, successful recall requires the conditional input distributions *p*(*R*∣*T* = ± *w*_*T*_) to have non-overlapping support across the decision boundary—a condition that is mathematically equivalent to maximal redundancy regardless of how the weights are obtained. We corroborate this finding with a closed-form derivation for Hebbian networks that reproduces the empirical profile of Fig. 2d, and holds across alternative PID measures and network sizes (Supplementary Notes 6.4 and 6.5).

Building on this insight, we construct infomorphic Hopfield networks where each neuron directly maximizes redundant information between its inputs. These networks significantly outperform classical Hebbian Hopfield networks in memory capacity and stability (subsection “Redundancy as a computational goal”). Further optimizing the redundancy-based objective enhances performance by suppressing irrelevant information contributions (subsection “Improving the capacity of the infomorphic goal”, Fig. 5c). Using only classical information-theoretic measures, we find that maximizing mutual information between target pattern elements and neuron output yields comparable performance to PID-based models, which can, however, be ascribed to an implicit optimization of redundancy (Fig. 4).

Across varying memory loads, the observed correspondence between high redundancy and recall accuracy supports our claim that it underlies successful associative memory learning (Fig. 4c). Finally, benchmarking against state-of-the-art methods confirms that infomorphic Hopfield networks achieve superior memory capacities, up to *α*_c_ = 1.70 [1.66, 1.71], while maintaining competitive stability (Fig. 6).

The approach of constructing local information-theoretic goal functions to perform associative memory learning has previously been suggested by Makkeh et al.^13^. In this work, we improve upon these results by replacing the complex custom activation function used by Makkeh et al.^13^ by a sign function during testing, which is replaced by a differentiable sigmoid for training, as well as a soft binning procedure, and demonstrate that *G* = Π_red_ and the classical goal *I*(*Y*: *T*) both achieve a memory capacity that is fourfold the capacity reported in ref. ^13^. Furthermore, we show that a direct optimization of the goal parameters further increases the memory capacity.

In addition, information theory has previously been employed to analyze Hopfield networks. Notably, the mutual information of the target pattern and the internal state *I*(***ξ***: ***Y***) has been prominently utilized. Dominguez and Bollé^20^ initially established *I*(***ξ***: ***Y***) as a suitable measure for evaluating the quality of pattern retrieval. Dominguez et al.^21,22^ subsequently demonstrated that *I*(***ξ***: ***Y***) was optimal for a sparsely connected network, which ultimately enhanced the memory capacity. Additionally, information-theoretic concepts have been used to train (deep) neural networks^13,16,23^. Previous research has demonstrated the significance of maximizing *I*(***ξ***: ***Y***) in enhancing the memory capacity, which in this work we explain by its implicit maximization of redundancy.

Our results demonstrate that maximizing redundancy directly can significantly improve memory capacity for Hopfield networks. However, promoting redundancy alone does not suffice to reach the theoretical optimum *α*_c_ = 2 of twice the number of neurons established by Cover^24^. Beyond classical associative memories, it has been shown that this bound can be exponentially exceeded by dense associative memories, which incorporate a layered architecture to improve performance^25,26^. Similarly, Schneider et al.^14^ constructed infomorphic networks with multiple layers, achieving substantial improvements in supervised learning tasks compared to single-layer networks. These findings open an intriguing avenue for future research: leveraging the framework of infomorphic networks to identify the information-processing mechanisms required to realize dense associative memories with hidden units. We hypothesize that the general principle of redundancy will continue to play a central role.

Furthermore, since the infomorphic update rule requires the estimation of the full joint probabilities, the approach is computationally expensive and biologically implausible on its own. Additionally, the interaction between the target weight scale *w*_*T*_ and network size, and their effect on the memory capacity is not yet fully understood. A more systematic investigation of this dependence is the subject of future work. However, the main advantage of the infomorphic approach lies in its flexible and interpretable goal functions. In future research, approximations to these learning rules may be devised which more implicitly achieve the principle of redundancy maximization.

A further important direction concerns correlated patterns. While it has been shown in classical Hebbian Hopfield networks that spatial correlation is detrimental to storage capacity^27^, we expect that infomorphic Hopfield networks could perform better in this setting, since infomorphic networks can better differentiate which target pattern elements are truly informative of the output. Promising first results in this direction are shown in Supplementary Note 7.

Another interesting research direction is to apply the principles of memory found here to complex objectives. For example, curiosity has been theorized as the seeking of novel patterns or information based on memorized patterns^28^. Infomorphic networks could reproduce this behavior by following a mixed goal, maximizing redundancy for memorization and unique information from the target for exploration.

In summary, Partial Information Decomposition (PID) and infomorphic networks form a powerful pair of tools for analysis and construction of associative memory networks. Together, they offer a new level of interpretability at the local level that can be used to assess the strengths and weaknesses of existing learning methods and to inspire the design of network architectures with state-of-the-art performance in associative memory tasks.

## Methods

### Hopfield networks

The *N* neurons of the Hopfield networks are updated based on the activity of other neurons in the network. Given a network state vector ${\{{y}_{i}(t)\}}_{i}$ at time *t*, the state of neuron *i* at the next timestep is computed as

3 $${y}_{i}(t+1)={\rm{sgn}}\left({r}_{i}\right),$$

where sgn (*x*) denotes the sign function and the recurrent input *r*_*i*_ is defined as a weighted sum of the activities of all neurons *y*_*j*_ as

4 $${r}_{i}=\sum_{j=1}^{N}{w}_{ij}{y}_{j}(t).$$

The originally proposed training method for the weights ${\{{w}_{ij}\}}_{ij}$ was Hebbian learning. In this learning rule, neurons which co-activate as part of the same pattern become more strongly connected, making the patterns attractors in the energy landscape. For bipolar patterns ***ξ***^(*p*)^ (see Fig. 1a for example patterns) with pattern index *p* (i.e., with pattern elements ${\xi }_{i}^{(p)}=\pm 1$) the resulting weights using the Hebbian learning rule are given by ref. ^29^

5 $${w}_{ij}=\mathop{\sum}\limits_{p=1}^{m}{\xi }_{i}^{(p)}{\xi }_{j}^{(p)},$$

where *m* is the total number of patterns. There are typically no self-connections in the network and therefore *w*_*ii*_ = 0. When training on uncorrelated patterns, the number of memories (patterns) *m*_*c*_ that a Hopfield network can successfully store scales linearly with network size *N*. The corresponding memory capacity is defined as ${\alpha }_{{\rm{c}}}=\frac{{m}_{{\rm{c}}}}{N}$ and depends on the learning method. The Hebbian rule has a capacity of *α*_H_ ≈ 0.14 ^15^, lower than the theoretical upper bound of *α*_c_ = 2 ^24,30^.

### Partial information decomposition

To understand how individual neurons contribute to associative memory function, we investigate the information processing at a single neuron. From an information-theoretic viewpoint, each neuron can be viewed as a channel that receives information about the outputs of other neurons via the recurrent input *R* and produces a bipolar output signal *Y*. The total entropy of a neuron’s output *H*(*Y*) can be decomposed into two parts, namely the information *I*(*Y*: *R*) explained by *R*, and the residual entropy *H*(*Y*∣*R*), such that *H*(*Y*) = *I*(*Y*: *R*) + *H*(*Y*∣*R*).

To evaluate whether this output information is relevant to the memory task, we introduce an auxiliary input variable *T* that corresponds to the targeted output for a given pattern and acts as a teaching signal for the neuron (Fig. 2a). While the total information which the recurrent and the new target input together hold about the neuron output *Y* is quantified by the joint mutual information *I*(*Y*: *R*, *T*), classical information theory cannot explain how this information is provided by the two source variables *R* and *T*: Some parts of the neuron’s firing might be explainable uniquely by the recurrent input (denoted by Π_unq,*R*_) and thus be unrelated to the target input, or uniquely by the target input, i.e., relevant but not encoded in *R* (Π_unq,*T*_). Other parts of the output information may be carried redundantly (Π_red_) in both sources, meaning they are both relevant and encoded in *R*, while yet others may be carried synergistically (Π_syn_), meaning that both information sources are necessary to uncover this piece of information. Enumerating and quantifying these information atoms is the subject of Partial Information Decomposition (PID)^8,31^.

The four PID atoms are related to the three classical mutual information terms via the so-called consistency equations^8^ (see Fig. 2c)

6 $$I(Y:R)=	 {{\Pi }}_{{\mathrm{unq}},R}+{{\Pi }}_{{\rm{red}}},\\ I(Y:T)=	 {{\Pi }}_{{\mathrm{unq}},T}+{{\Pi }}_{{\rm{red}}},\\ I(Y:R,T)=	 {{\Pi }}_{{\mathrm{unq}},R}+{{\Pi }}_{{\mathrm{unq}},T}+{{\Pi }}_{{\rm{red}}}+{{\Pi }}_{{\rm{syn}}}.$$

This set of linear equations is underdetermined and leaves open a degree of freedom in the values of atoms Π. To resolve this underdetermination, an additional quantity needs to be defined, which is usually a measure for redundancy. Throughout the literature, a plethora of different redundancy measures have been devised, which fulfill distinct requirements and have different operational interpretations [e.g., ref. ^32^ and references therein]. Throughout this work, we use the ${I}_{\cap }^{{{{\rm{sx}}}}}$ measure introduced by Makkeh et al.^33^, due to its differentiability, which is essential for optimization (see Supplementary Note 1.3). Using PID now allows for a more fine-grained decomposition of the entropy of *Y* into five components

7 $$H(Y)={{\Pi }}_{{\mathrm{unq}},R}+{{\Pi }}_{{\mathrm{unq}},T}+{{\Pi }}_{{\rm{red}}}+{{\Pi }}_{{\rm{syn}}}+{H}_{{{{\rm{res}}}}},$$

where *H*_res_ = *H*(*Y*∣*R*, *T*) denotes the residual entropy of the output *Y* not explained by either source variable *R* or *T* (see Supplementary Note 1.1 for explicit calculations of the information atoms).

### Information-theoretic learning

PID provides a valuable framework to analyze the information processing of an individual neuron in a Hopfield network. However, to investigate whether this information processing view provides a sufficient description of the neurons’ functions, PID can also be employed to build infomorphic neurons^13^, which optimize a PID-based goal function directly.

During evaluation, these infomorphic networks operate analogously to classical Hebbian Hopfield networks with the neuron’s activity given by Eq. (3). The network state is initialized in a target pattern, and neurons synchronously update their output based on both inputs until convergence or up to 100 time steps using the same update rule (Eq. (3)).

For training only, the true pattern element is introduced as an additional target input *T* to each neuron to guide the learning. The recurrent dynamics are updated only once, this time utilizing a sigmoid activation function to produce a firing probability and stochastically assigning +1 or −1 to *Y* according to this probability. After this, the joint input distribution is estimated using a differentiable soft binning approach (see Supplementary Note 4), enabling computation of PID atoms using the discrete ${I}_{\cap }^{\,{\mbox{sx}}\,}$ PID measure. Given these PID atoms, a general infomorphic objective function of the form

8 $$G={\gamma }_{{\mathrm{unq}},R}{{\Pi }}_{{\mathrm{unq}},R}+{\gamma }_{{\mathrm{unq}},T}{{\Pi }}_{{\mathrm{unq}},T}+{\gamma }_{{\mathrm{red}}}{{\Pi }}_{{\mathrm{red}}}+{\gamma }_{{\mathrm{syn}}}{{\Pi }}_{{\mathrm{syn}}}+{\gamma }_{{\rm{res}}}{H}_{{\rm{res}}},$$

can be evaluated, where *γ*_*i*_ are fixed parameters that control the maximization and minimization of individual information atoms. Finally, the weights of each infomorphic neuron are locally updated by gradient ascent on *G* using automatic differentiation.

Performance is assessed using cosine similarity between recalled and target patterns, with accuracy quantified both as an average over all patterns (${a}_{\cos }$) and via a threshold measure (*a*_*θ*_) to obtain the fraction of patterns recalled with above *θ* = 95% accuracy. The memory capacity (*α*_c_) is determined by evaluating accuracy at different memory loads using different random seeds, and stability is quantified by measuring the maximum fraction of corrupted bits ${f}_{\max }$ in the initial network state. More details can be found in Supplementary Note 1.

## Supplementary information

Supplementary Information

Transparent Peer Review file

## Data Availability

The simulation data generated in this study can be reproduced using the code and parameters described in the Methods and Supplementary Information. The code is available at https://github.com/Priesemann-Group/Infomorphic_Networks. No external datasets were used in this study.

## Code availability

The code to reproduce the results is available via the public GitHub repository https://github.com/Priesemann-Group/Infomorphic_Networks.
