# Supplementary material for: Redundancy maximization as a principle of associative memory learning in Hopfield networks
Source: Nat Commun. 2026 Nov 6;17:11499. doi: 10.1038/s41467-026-78777-2 (PMC13743752; doi:10.1038/s41467-026-78777-2)
Supplement: Supplementary file 1 [file 41467_2026_78777_MOESM1_ESM.pdf]

Supplementary Information

for

Redundancy Maximization as a Principle of Associative Memory Learning in Hopfield Networks

Mark Blümel†, Andreas C. Schneider†, Valentin Neuhaus†, David A. Ehrlich, Marcel Graetz, Michael Wibral, Abdullah Makkeh, Viola Priesemann

1 Supplementary Methods

1.1 Calculating PID atoms

In the case of two source variables, the calculation of all PID atoms follows from the three consistency equations

$$\begin{aligned} I(Y:R) &= \Pi_{\text{unq},R} + \Pi_{\text{red}}, \\ I(Y:T) &= \Pi_{\text{unq},T} + \Pi_{\text{red}}, \\ I(Y:R,T) &= \Pi_{\text{unq},R} + \Pi_{\text{unq},T} + \Pi_{\text{red}} + \Pi_{\text{syn}}. \end{aligned} \tag{1}$$

and one additional axiom. In our case, we define redundant information using the $I_\cap^{\text{sx}}$ measure introduced by Makkeh et al. [1], obtaining

$$\begin{aligned} \Pi_{\text{red}} := I_\cap^{\text{sx}}(Y:R;T) &:= \sum_{r,t,y} p(r,t,y) i_\cap^{\text{sx}}(y:r;t) \\ &:= \sum_{r,t,y} p(r,t,y) \log \frac{p(y, r\cup t)}{p(y)p(r\cup t)} \\ &:= \sum_{r,t,y} p(r,t,y) \log \frac{p(y,r)+p(y,t)-p(y,r,t)}{p(y)[p(r)+p(t)-p(r,t)]}. \end{aligned} \tag{2}$$

Given this definition, the other atoms are calculated via Moebius inversion:

$$\begin{aligned} \Pi_{\text{unq},R} &= I(Y:R) - \Pi_{\text{red}}, \\ \Pi_{\text{unq},T} &= I(Y:T) - \Pi_{\text{red}}, \\ \Pi_{\text{syn}} &= I(Y:R,T) - \Pi_{\text{unq},R} - \Pi_{\text{unq},T} - \Pi_{\text{red}}. \end{aligned} \tag{3}$$

1.2 Infomorphic Hopfield Network Set-up

The architecture of infomorphic Hopfield networks is similar to that of the classical Hopfield network introduced in the main text Methods section. The number of infomorphic neurons N corresponds to the number of pattern elements of the target patterns to be stored in the recurrent weights of the network. During training, each neuron receives two different inputs. The first input is a weighted sum of the outputs of the recurrently connected neurons

$$r_i = \sum_j^N w_{ij}^R y_j. \tag{4}$$

The second, target input T is given by the (weighted) element of the target pattern that the neuron encodes, i.e.,

$$t_i = w_i^T \xi_i^{(p)}. \tag{5}$$

Given these two inputs, the probability of neuron i returning state $+1$ is given by

$$p(y_i = +1) = \sigma\left(r_i + t_i\right), \tag{6}$$

with σ indicating the sigmoid function, while the probability of state -1 is given by $1 - p(y_i = +1)$.

1.3 Training

By treating the inputs and the output as random variables, we apply PID to optimize specific information-processing objectives. Before training, the network and optimizer are initialized according to Table 1 in subsection 1.5.

The training procedure itself begins by initializing the network state in the target pattern $\xi^{(p)}$ and presenting single elements $\xi_i^{(p)}$ of the same pattern via the target input t as teaching signal to each corresponding neuron i. The network is then run for one time step in a synchronous manner to obtain the recurrent inputs r and the output probabilities $p(Y = y \mid R = r, T = t)$ according to Equation 6. Over all patterns, we estimate the empirical joint probability mass functions $p(R = r, T = t)$ of R and T for each neuron using the (soft) plug-in binning method described in section 4.

Given these probability estimates, the PID terms are computed as described in Supplementary Notes 1.1. Then, the objective function G is evaluated according to the general infomorphic objective defined in the main text Methods section.. Finally, the recurrent weights $\boldsymbol{w}_R$ of each neuron are updated via gradient ascent on G, using automatic differentiation. While in principle the target weight w_T of each neuron could also be updated, in practice a constant weight of order 1 resulted in fastest learning and best performance. Specifically, we chose a constant $w_T = 2.3$.

1.4 Testing

To evaluate the performance of the trained network, we use a procedure similar to Hopfield. First, the neurons are initialized in the pattern $\xi^{(p)}$. As before, the recurrent inputs are given by Equation 4, but the neurons do not receive target input signals. The bipolar output of the neurons is then given by

$$y_i(t + 1) = \operatorname{sgn}(r(t)). \tag{7}$$

This step is repeated until the network converges to a fixed point or limit cycle, or for a maximum of 100 iterations. The network's performance is then evaluated based on the cosine similarity between the final network state $\mathbf{y}(t_f)$ and the target pattern $\boldsymbol{\xi}_i^{(p)}$. We use two measures to aggregate the individual similarities and arrive at an overall accuracy score. The first is the mean accuracy

$$a_{cos} = \frac{1}{P} \sum_p \langle(\mathbf{y}(t_f), \boldsymbol{\xi}^{(p)})\rangle, \tag{8}$$

where we average alignment both across neurons and across patterns. The second is the threshold accuracy a_θ

$$a_\theta = \frac{1}{P} \sum_p \mathcal{H}(\langle(\mathbf{y}(t_f), \boldsymbol{\xi}^{(p)})\rangle - \theta), \tag{9}$$

where $\mathcal{H}$ is the Heaviside function and θ the threshold parameter, which we set to $\theta = 0.95$. For this measure to be non-zero, at least some patterns must be restored with high accuracy.

To arrive at the memory capacity of a given learning method, we validate it on 20 different seeds. On each seed, we train networks with memory loads α increasing with a step size of 0.02. We take as the memory capacity the value α_c, at which for all $\alpha < \alpha_\mathrm{c}$, the network encodes memories with $a_{\cos}(\alpha) > 0.95$ (excluding finite size effects). Unless mentioned otherwise, we take the median over all validation seeds to arrive at a memory capacity.

1.4.1 Stability

In addition to estimating the performance, we also estimate the stability of the stored memories. We test the stability by introducing an increasing fraction of flips f to a pattern $\xi^{(p)}$ to initialize the network state and then measuring the accuracy $a_{\cos}(f)$. The stability $f_{\max}$ is given by the maximum fraction of flips under which the original memory can still be recovered with an accuracy $a_{\cos}(f_{max}) \geq \epsilon$ of at least $\epsilon = 0.95$. By varying the memory load α before measuring the stability, we can obtain a full stability profile $f_{\max}(\alpha)$. The area under the curve $f_{\max}(\alpha)$ can be viewed as a proxy for the overall level of stability achieved by a given rule. The choice of parameters is taken as in Tolmachev and Manton [2].

1.5 Model Parameters

In Table 1, we explain the parameters of infomorphic networks and their values that were used during training unless specified otherwise.

Supplementary Table 1: The model parameters.

Parameter	Definition/Meaning	Value
N	number of neurons	100
$\boldsymbol{w}_T^{\text{Hebb}}$	initialization of the target weights used in Hebbian setup	$10^{-10} \cdot \mathbf{1}_N$
$\boldsymbol{w}_T^{\text{Info}}$	initialization of the target weights used in infomorphic setup	$2.3 \cdot \mathbf{1}_N$
$\boldsymbol{w}_R$	initialization of the recurrent weights	$\sim \mathcal{N}\left(0, \lambda_r \sqrt{2/N}\right)$
λ_r	initial scale of recurrent weights	$1 \cdot 10^{-3}$
optimizer	algorithm to maximize G	Adam
η	learning rate	0.05
epochs	number of complete passes of the entire input patterns	5000
reps	number of consecutive times the pattern is presented	1
n_t	number of bins in target direction	2
n_r	number of bins in recurrent direction	60
σ_t	target kernel width as fraction of bin width	$1 \cdot 10^{-6}$
σ_r	recurrent kernel width as fraction of bin width	0.5
padding	how far the binning range extends beyond the data	1
sequential	True if states are updated sequentially	False
N_{iter}	maximum iterations during testing	100
θ	testing threshold for a_θ	0.95

2 Pseudocode for Training

Function 1: TrainHebbianHopfieldModel

Input: data

Output: trained model

```
INITIALIZE model;
INITIALIZE model.neuron_weights ← zero_matrix;
foreach pattern in data do
    model.neuron_weights ← model.neuron_weights + outer(pattern, pattern);
return model
```

3 Optimization Results

Each run of the optimization algorithm takes place on a single seed, producing a set of best goals which cluster close together. We have taken one candidate goal function from each of the 10 optimization results and verified them on a set of 20 different seeds. The results are depicted in Figure 5 of the main text. Out of these ten candidate goals, the best two were chosen for the corresponding Figure 6.

Function 2: TrainInfomorphicHopfieldModel

Input: data, num_epochs, goal_params

Output: trained model

```
INITIALIZE model;
foreach epoch in range(num_epochs) do
    INITIALIZE model_outputs;
    foreach pattern in data do
        INITIALIZE network_state ← pattern;
        network_state ← model.forward_network(t=pattern, r=network_state);
        model_outputs.append(network_state);
    foreach neuron in model do
        TrainInfomorphicNeuron(neuron, goal_params, y=output_state[neuron], t=data, r=model_outputs);
return model
```

Function 3: TrainInfomorphicNeuron

Input: neuron, goal_params, y, r, t

Output: None

BIN continuous values r in 60 and t in 2 equally sized bins;

COUNT occurrences of tuples (r, t);

COMPUTE empirical probability masses $p(r, t)$;

EVALUATE conditional probabilities $p(y \mid r, t)$ from the neurons;

CONSTRUCT full joint probability mass function $p(y, r, t) = p(r, t)p(y \mid r, t)$;

isx_redundancies ← ComputeIsxRedundancies($p(y, r, t)$);

pid_atoms ← ComputePIDAtoms(isx_redundancies);

goal ← scalar_product(goal_params, pid_atoms);

PERFORM autograd to maximize goal;

UPDATE neuron.weights;

Function 4: ComputeIsxRedundancies

Input: Joint probability mass function $p(y, r, t)$

Output: Isx Redundancy Measure Values

foreach antichain $\beta \in \Big\{\{\{1\},\{2\}\},\{\{1\}\},\{\{2\}\},\{\{1,2\}\}\Big\}$ **do**

  COMPUTE conditional probability mass functions $p(Y = y \mid \bigvee_{\boldsymbol{b}\in\beta} \bigwedge_{i\in\boldsymbol{b}} S_i = s_i)$;

  COMPUTE marginal probability mass function $p(Y = y)$;

  $I_\cap^{\mathrm{sx}}(Y : S_\beta) \leftarrow \sum_{y,r,t} p(Y = y, R = r, T = t) \log_2 \frac{p(Y=y \mid \bigvee_{\boldsymbol{b}\in\beta} \bigwedge_{i\in\boldsymbol{b}} S_i = s_i)}{p(Y=y)}$;

return $I_\cap^{\mathrm{sx}}(Y : S_\beta)$ for all antichains β

Supplementary Table 2: Estimates for the memory capacities shown in the main text, including the bootstrapped 95% confidence intervals. Additionally, the goals and capacities for other local optima found during the optimization procedure described in the main text are shown.

Goal Name	Capacity		Goal Parameter				
	Median	95% CI	$\gamma_{\text{unq},T}$	$\gamma_{\text{unq},R}$	γ_{red}	γ_{syn}	γ_{res}
Redundancy Π_{red}	1.59	[1.56, 1.61]	0	0	1	0	0
Target information $I(Y:T)$	1.62	[1.61, 1.64]	1	0	1	0	0
	1.60	[1.58, 1.62]	0.52	-0.41	0.94	-0.31	0.57
	1.64	[1.62, 1.65]	0.43	-0.51	0.72	-0.65	-0.05
	1.66	[1.63, 1.67]	0.26	-0.38	0.78	-0.4	-0.34
	1.66	[1.64, 1.68]	0.18	-0.36	0.68	-0.51	-0.17
	1.66	[1.62, 1.69]	0.41	-0.08	0.8	-0.17	0.04
	1.68	[1.65, 1.7]	0.06	-0.16	0.78	-0.08	-0.37
	1.68	[1.66, 1.7]	0.84	0.16	0.92	0.36	-0.46
	1.7	[1.66, 1.72]	0.26	-0.38	0.78	-0.4	-0.34
Optim. (i)	1.68	[1.65, 1.72]	-0.27	-0.68	0.68	-0.77	-0.80
Optim. (ii)	1.7	[1.66, 1.71]	0.48	-0.16	0.25	0.04	-0.63

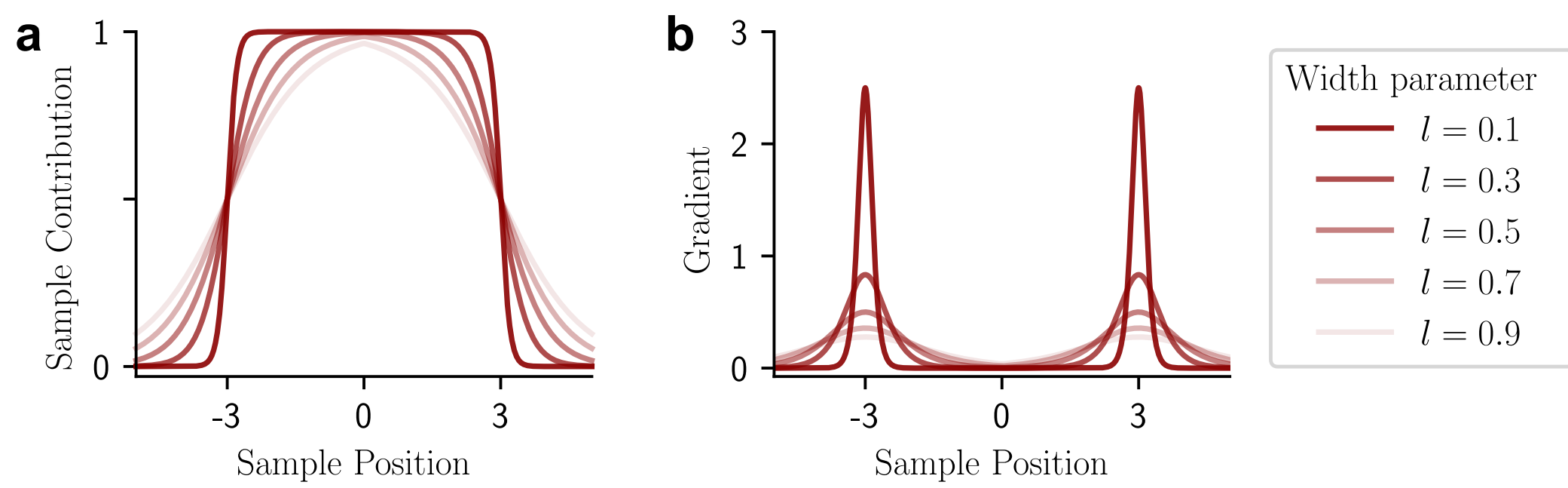

Supplementary Figure 1: **In the soft binning procedure, the histograms are smoothed at the bin edges resulting in non-zero gradients of the histogram. a** The contribution of a single sample to a bin covering the range $[-3, 3]$. The contribution scales with the distance to the center of the bin. **b** The corresponding magnitude of gradients to **a**. If l approaches zero, we converge to hard bin edges and the gradients become delta peaks.

4 Binning

4.1 Differentiable Binning

The PID terms are estimated using a discrete PID measure due to the lack of a differentiable PID measure for mixed discrete-continuous variables [3]. Thus, the continuous-valued inputs r and t are binned to allow estimating a discrete probability distribution $p(r,t)$. Since this operation is not differentiable, previous approaches to estimating the PID terms assumed that $p(r,t)$ is constant with respect to the weights w_R and $\boldsymbol{w}_T$, resorting to estimating $p(r,t)$ from a static histogram [4]. Taking into consideration the small changes in $p(r,t)$ with respect to the weights, however, might improve the PID estimates. Therefore, we implemented a kernel-based 'soft binning' approach that smooths the histogram at the bin edges providing estimates of $p(r,t)$ gradients (Figure 1).

4.2 Soft Binning Procedure

The soft binning procedure is composed of three steps. First, for each bin the distances d between its edges and the coordinates of every sample (r, t) in all dimensions is calculated. Then, each bin is assigned weights according to a kernel K for every sample. The choice of K and its properties are discussed in subsection 4.3. Finally, the bin weights obtained for every sample are normalized, so that the total count of the histogram adds up to the number of samples.

4.3 Kernel choice

The kernel $K(d)$ should be differentiable, monotonically fall to zero as distance of the bin to the sample increases and approach a delta function as its width parameter decreases. We chose a sigmoid kernel $K(d) = \Pi_{i\in\{x,y\}}\sigma((c_i/2-d)/l)$ that satisfies all of these constraints, where c_x and c_y are the the histogram sizes and l is the width parameter. As l tends to zero, the non-differentiable binning values are recovered (Figure 1).

Note that the normalization step in the binning procedure ensures that each sample has an equal impact on the total results. Otherwise, especially when l is smaller than half a bin size, samples that by chance lie closer to the center of a bin are more impactful than those that lie closer to an edge.

Since the size of the histogram scales throughout learning, $K(d)$ is also scaled in proportion to the bin size. This is done in each epoch by resetting the width parameter $l = f_l\sqrt{(c_x/2)^2 + (c_y/2)^2}$ for a fixed fraction f_l and with side lengths of a bin c_x and c_y.

5 Scaling Behavior

5.1 Scaling of Capacity and Stability

One of the fundamental properties of Hopfield networks is that the memory capacity α_c scales linearly with the network size N. To support this claim for infomorphic Hopfield networks, we repeated the experiments from the main text memory-capacity and performance-comparison figures at different network sizes (Figure 2). Both the memory load α and the stability $f_{\max}$ are defined as fractions of the network size N and should thus produce the same curve at different N. For the memory capacity, we compare at sizes $N \in [50, 100, 200, 400, 800]$. For sizes 50–400, all hyperparameters are unchanged, and the transition around $\alpha_c^{\text{red}} \approx 1.59$ stays the same. For $N = 800$, we repeated the experiments twice with different values for $|\boldsymbol{w}_T| \in \{2.3, 3\}$. For $|\boldsymbol{w}_T| = 2.3$, the capacity falls to $\alpha_c \approx 1.41$, while it increases to $\alpha_c \approx 1.66$ for $|\boldsymbol{w}_T| = 3$. These moderate changes indicate that important parameters may have different optimal values at different scales, and that in particular the external target weights have to be adapted to the amount of internal recurrent input a neuron receives.

We find that stability also scales linearly with network size. There are small increases in $f_{\max}$ as a function of α across most memory loads. However, only the maximum memory load with $f_{\max} > 0$ keeps increasing with network size. The other increases in $f_{\max}$ saturate for large networks.

In addition, the redundancy estimates for Hebbian networks are also independent of network size (see Figure 3).

5.2 Finite Size Effects

At small network sizes and very small memory loads, a small portion of neurons will receive constant target inputs with an entropy of $H(T) = 0$ and thus $I(Y : T) = 0$ and $\Pi_{\text{red}} = 0$, leading to reduced recall accuracy (Figure 4). This effect happens if neurons receive the same target input $T \in \{-w_T, w_T\}$ across all patterns. Writing the absolute number of memory patterns as $m = \alpha N$, and considering uncorrelated patterns, the expected number of neurons that receive only one target label can be written as $\langle N_{\text{const}}\rangle(\alpha) = \frac{N}{2^{\alpha N-1}}$, which goes to 0 for large N.

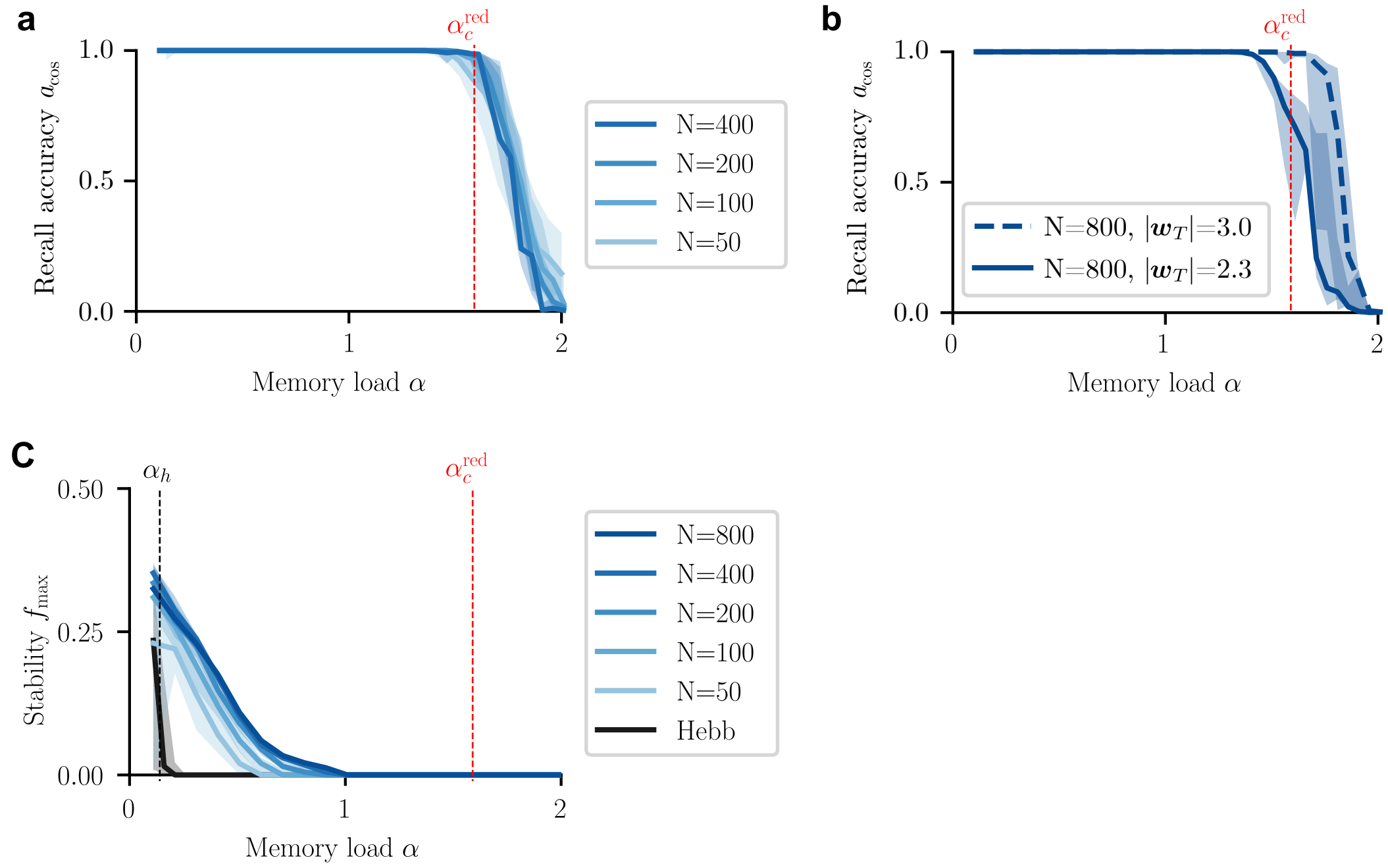

Supplementary Figure 2: **Network capacity and stability increases linearly with network size, with minor increases to capacity. a** Accuracy of networks trained with the redundancy rule is shown as a function of memory load α for different network sizes N. This figure reproduces the main text memory-capacity results with neuron counts N between 50 and 400. As the number of neurons increases, the drop in accuracy at α_c^{red} becomes sharper. The median over 20 runs is shown. **b** For $N = 800$, scaling the target weights from 2.3 to 3.0 leads to retaining high α_c. Here, the median over 5 runs is shown. **c** The stability $f_{\max}$ of networks trained on redundancy as a function of memory load α. This figure reproduces the main text memory-capacity results with neuron counts N between 50 and 400. The shape of the stability curve remains similar as network size is increased. The median over 20 runs is shown. For $N = 400, 800$ this was reduced to 5 runs.

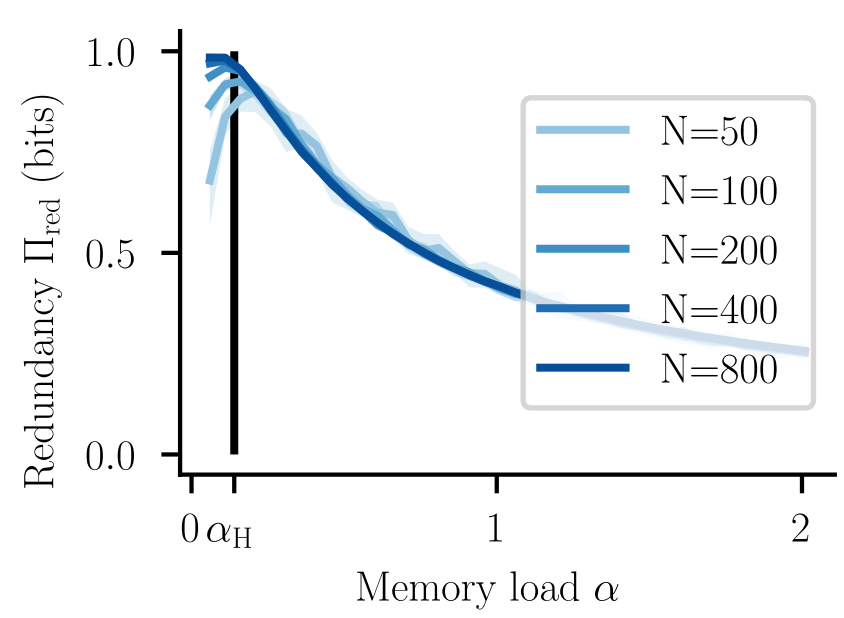

Supplementary Figure 3: **Redundancy estimates are robust with respect to network size.** The main text redundancy profile from Figure 2d for Hebbian networks at different network sizes N. The curves show the median over 20 network initializations, with values first averaged across all neurons. For very small memory loads the estimate converges due to finite size effects as explained in Supplementary Notes 5.2 (Figure 4).

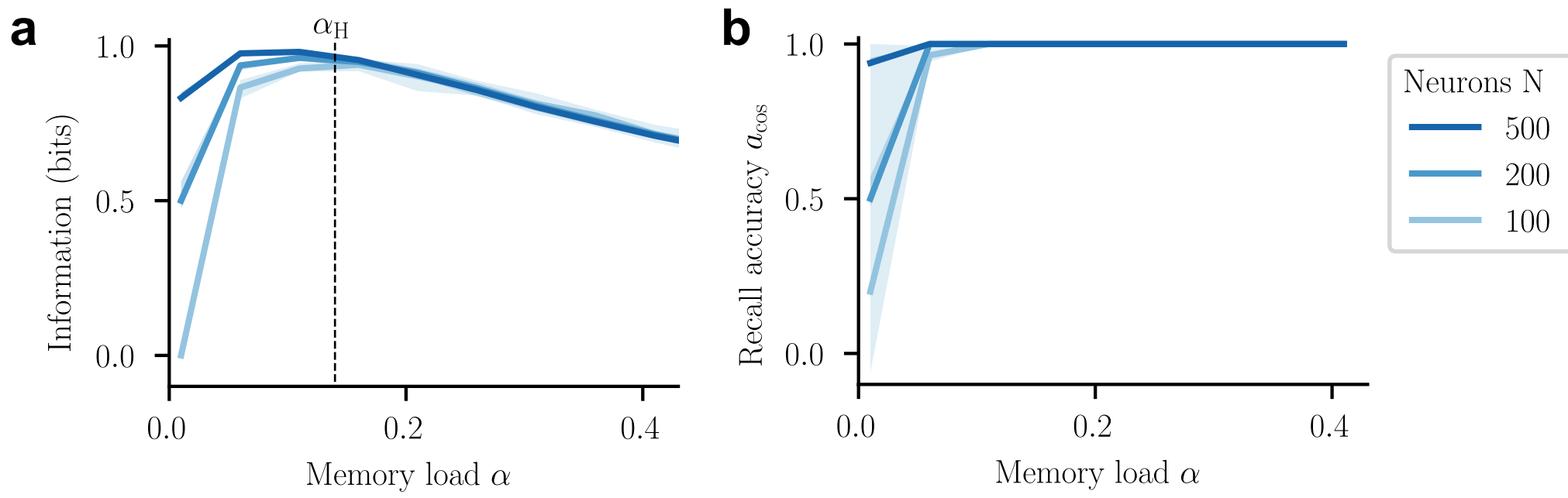

Supplementary Figure 4: **Finite size effects for very low memory loads can be counteracted by increasing the network size. a** The mean redundancy of neurons for different network sizes shows that finite size effects can be reduced for small memory loads α by increasing the number of neurons. For higher memory loads, those effects become smaller. **b** For networks trained under the redundancy goal, missing redundancy leads to unsuccessful training for small networks and small memory loads α. In **a**, values are first averaged across all neurons, and the curve then shows the median of these averages across the initializations. In **b**, the curve shows the median calculated across 20 network initializations. For both panels, the shaded area represents the central 90% of the data.

At finite sizes, however, the number of atoms without any entropy can become significant. In particular, for the low memory loads below the Hebbian capacity α_H this leads to an underestimation of the average redundancy of the network. This effect can be reduced by increasing the number of neurons while keeping α fixed (Figure 4a). This explains why the finite size effects can only be observed for small memory loads and can be countered by increasing the number of neurons as done in the main text. For infomorphic neurons, the finite size effects also prevent learning successfully at small memory loads α in small networks (Figure 4b). Because of the higher capacity of infomorphic networks, they are less important at the memory capacity α_c^{red}.

6 Derivation of Redundancy for Associative Memory

6.1 Redundancy as a Necessary Requirement for Associative Memory

In the following, we provide further intuition for why high redundancy is found in Hebbian Hopfield networks as well as other learning algorithms.

For successful recall of memories, each neuron's activity needs to be aligned with its respective target value, i.e. $Y = 1$ iff $T > 0$. Both in the Hebbian and infomorphic Hopfield networks we use a Heaviside activation function in the test phase, implying that $Y = 1$ iff $R \geq 0$. The goal of training thus becomes to change the recurrent weights such that the conditional distributions for each target value become disjoint and restricted to one quadrant of the R-T-plane each, with $p(r \mid T \geq 0) > 0$ only if $r > 0$, and vice versa $p(r \mid T < 0) > 0$ only if $r < 0$ (see Figure 6a).

We now show that this separation condition directly implies maximal redundancy. We start from the $I_\cap^{\text{sx}}$ redundancy measure given in Equation 2, which can be written as

$$\begin{aligned} I_\cap^{\text{sx}} &= \sum_{r,t,y} p(r,t,y) \log \frac{p(y, r \cup t)}{p(y)p(r \cup t)} \\ &= \sum_{r,t,y} p(r,t,y) \log \frac{p(y,r) + p(y,t) - p(y,r,t)}{p(y)[p(r) + p(t) - p(r,t)]}. \end{aligned} \tag{10}$$

We first inspect the second line of Equation 10. If the two conditional distributions $p(R \mid T = w_T)$ and $p(R \mid T = -w_T)$ are restricted to the first and third quadrant, respectively and the output Y is predicting the target T correctly, then

$\forall y, r, t : p(y, r, t) > 0 \implies p(y, r, -t) = p(y, -r, t) = 0$. As a result, the union probability in the denominator (indicated by a purple cross in Figure 6) collapses to

$$\begin{aligned} p(r \cup t) &= p(r) + p(t) - p(r, t) \\ &= p(r, t) + p(r, -t) + p(t) - p(r, t) \\ &= p(r \mid -t) p(-t) + p(t) \\ &= p(t) \end{aligned} \tag{11}$$

for all (r, t) with non-zero probability mass $p(r, t)$. By the same logic, the union probability in the numerator collapses to $p(y, r \cup t) = p(y, t) = p(t)$. Combining these effects leads to redundant information taking on its maximum value given by the entropy of the target signal as

$$I_\cap^{\text{sx}} = \sum_{r,t,y} p(r, t, y) \log \frac{p(y, r \cup t)}{p(y) p(r \cup t)} \tag{12}$$

$$= \sum_{r,t,y} p(r, t, y) \log \frac{1}{p(y)} = H(Y) = H(T) = 1\,\text{bit}. \tag{13}$$

In the case of imperfect prediction, $p(y \mid t) < 1$ and $I_\cap^{\text{sx}} = I(Y : T)$. This mutual information is maximized if and only if the neuron always fires when $T > 0$ and never fires when $T < 0$ or vice versa. Note that information theory is agnostic to signs, such that the case of $p(\operatorname{sgn} R \neq \operatorname{sgn} T) = 1$ also maximizes redundancy. Makkeh et al. [5] dealt with this case by training a separate decoder, here we preclude it through positive target weights w_T.

Next, we show that any deviation from the separated supports necessarily decreases the redundancy. If the target weights w_T are close to zero, as is the case in Hebbian learning, the Heaviside activation function $p(y|r, t) = \delta_{y,\operatorname{sgn}(r+t)}$ reduces to $\delta_{y,\operatorname{sgn}(r)}$ for almost all inputs, which produces

$$\begin{aligned} I_\cap^{\text{sx}} &= \sum_{r,t,y} p(r, t, y) \log \frac{p(y, r \cup t)}{p(y) p(r \cup t)} \\ &= \sum_{r,t,y} \delta_{y,\operatorname{sgn}(r)} p(r, t) \log \frac{p(y, r) + p(y, t) - p(y, r, t)}{p(y)[p(r) + p(t) - p(r, t)]} \\ &= \sum_{r,t} p(r, t) \log \frac{p(Y = \operatorname{sgn} r, r) + p(Y = \operatorname{sgn} r, t) - p(Y = \operatorname{sgn} r, r, t)}{p(y)[p(r) + p(t) - p(r, t)]} \\ &= \sum_{r,t} p(r, t) \log \left[\frac{p(r) + \sum_{\operatorname{sgn} r' = \operatorname{sgn} r} p(r', t) - p(r, t)}{p(y)[p(r) + p(t) - p(r, t)]} \right]. \end{aligned} \tag{14}$$

In general, we have $\sum_{\operatorname{sgn} r' = \operatorname{sgn} r} p(r', t) \leq p(t)$. In particular, this turns into a strict inequality if the conditional probability $p(R \mid T = t)$ has support for both positive and negative values of r. In this case, the logarithm will become negative and consequently the redundant information will not be maximal, $I_\cap^{\text{sx}} < 1 = H(Y)$. From this, it follows that redundancy can only be maximal when the two conditional distributions $p(R \mid T = t)$ have support on opposite sides of the $R = 0$ line, and thus are non-overlapping. Since the disjoint probability mass functions are the only way to achieve perfect recall accuracy, redundancy is therefore necessary for associative memory function.

6.2 Optimizing Redundancy in Infomorphic Networks

Having established redundancy as necessary for associative memory function, it remains to be shown that it can also function as a goal to be optimized directly in infomorphic networks.

An important distinction to the Hopfield setup discussed so far is that during training, infomorphic networks use a sigmoid activation function. Thus, the probability of an output Y is dependent on the magnitude of the activation

function's inputs R and T. For our case of fixed target weights w_T, gradient-based learning can only change the recurrent weights and thus move the conditional probability distributions along the r-axis in Figure 6. Under the sigmoid activation function a global maximum is obtained for the support of $p(R \mid T)$ going to $\pm\infty$. The sigmoid thus provides smooth gradients towards the optimal weight configuration, making gradient-based learning possible.

In practice, networks trained on redundancy learn to separate the two conditional distributions $p(R \mid T = \pm 1)$. Instead of the normal distributions found in Hebbian learning, recurrent inputs R are skewed towards larger absolute values (Figure 5).

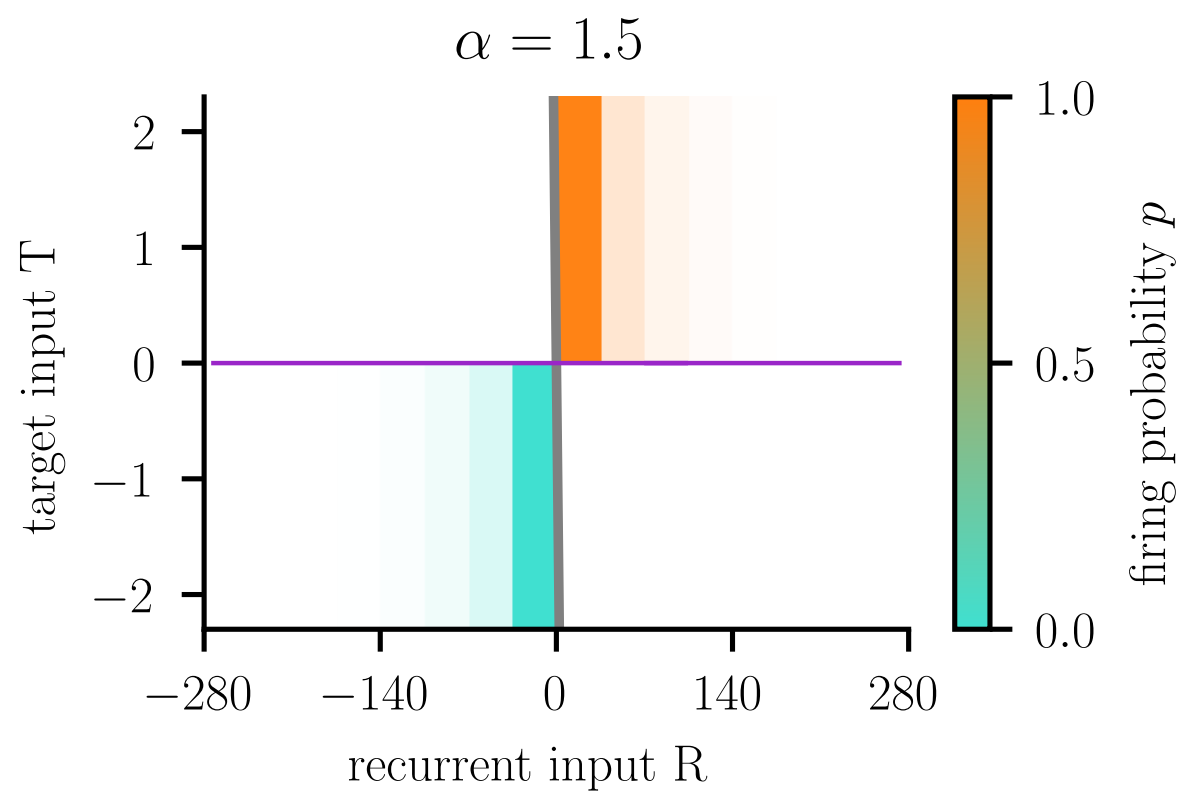

Supplementary Figure 5: **Training on redundancy leads to asymmetric recurrent input distributions with disjoint supports.** Joint distribution of inputs (R, T) for a network trained on redundancy at memory load $\alpha = 1.5$. Hue indicates the firing probability $p(Y = +1 \mid R, T)$ and saturation the sample density. The conditional distributions $p(R \mid T = \pm w_T)$ have disjoint supports on opposite sides of $R = 0$, in agreement with the geometric condition for maximal redundancy and successful recall (Supplementary Notes 6.1). The shape of the distribution is asymmetric, unlike the symmetric Gaussian distributions found in the Hebbian case.

6.3 Analytical Derivation of Redundancy in Hebbian Hopfield Networks

Hebbian Hopfield networks in particular allow for an analytical derivation of the redundancy for a given memory load.

First, we take the definition of the joint probability distribution $p(y, r, t) = p(t)p(r \mid t)p(y \mid r, t)$, where $y \in \{-1, +1\}$ is the neuron output, r the recurrent input and $t \in \{-1 \cdot w_T, +1 \cdot w_T\}$ the pattern element. In the main text, the pattern element was defined as $t = w_T \xi_i^{(q)}$. Since w_T only scales the label of the pattern input but does not enter the network dynamics, the joint distribution $p(y, r, t)$ is invariant under the choice of w_T. We therefore set $w_T = 1$ without loss of generality. Using that $y = \mathrm{sgn}(r)$ is a deterministic function of r, we can rewrite the joint probability distribution as

$$p(y, r, t) = \frac{1}{2} \cdot p(r \mid t) \cdot p(y \mid r), \tag{15}$$

where $p(y \mid r) = \delta_{y,\mathrm{sgn}(r)}$. So we only have to define $p(r \mid t)$. Following the derivation of McEliece et al. [6], we decompose the local field using the Hebbian weights as defined in the main text. For a network with N neurons storing m patterns and assuming perfect recurrent input r of a pattern $\xi^{(q)}$ ($y_j = \xi_j^{(q)}\ \forall j \neq i$), the local field of a single neuron i becomes

$$r_i^{(q)} = \sum_{j \neq i}^{N} w_{ij}\, \xi_j^{(q)} = (N-1)\, \xi_i^{(q)} + \sum_{j \neq i}^{N} \sum_{p \neq q}^{m} \xi_i^{(p)}\, \xi_j^{(p)}\, \xi_j^{(q)}. \tag{16}$$

The assumption holds, if we look at the first iteration of the Hopfield network as done in the main part of the paper. Each of the $\xi_i^{(p)} \xi_j^{(p)} \xi_j^{(q)}$ in the double sum is a Rademacher random variable (i.e., ± 1 with equal probability), and there

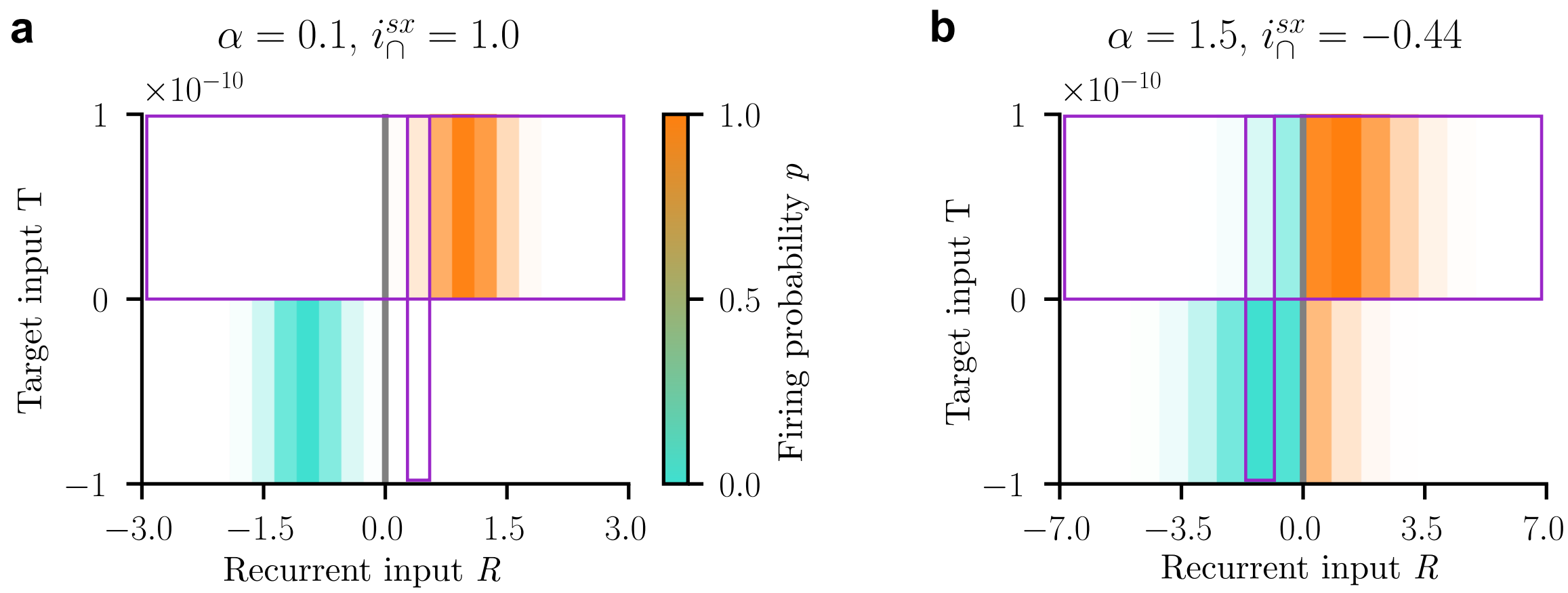

Supplementary Figure 6: **Redundancy measures the stability of associative memory solutions. a** Input distribution for neurons in a Hebbian network trained at a memory load of $\alpha = 0.1$, below the memory capacity α_H. The firing probability p is indicated by hue and the frequency of inputs by saturation. The recurrent inputs R are aligned to the sign of T with no misaligned samples despite some variance. The pointwise redundancy considers all bins with either the same R or the same T, represented by the purple cross. Here, redundancy is high across samples, since the distributions neither overlap nor cross the decision boundary indicated in gray. **b** Input distribution for Hebbian networks trained at a memory load of $\alpha = 1.5$, well above the memory capacity. The variance in recurrent inputs R has increased to the point that a relevant fraction of inputs are misaligned w.r.t. the target T. Redundancy is reduced because for fixed target, the neuron has non-zero probability for both outputs. In addition, for some bins the vertical component of pointwise redundancy also includes samples from the other distribution.

are $L = (N-1)(m-1)$ such independent terms. If k of the L terms equal $+1$, the sum evaluates to $2k - L$, with $k \sim \text{Binomial}(L, \frac{1}{2})$. Using our definition of the t input ($t = \xi_i^{(q)}$), the conditional distribution can be written as

$$p\left(r \mid t\right) = \binom{L}{\frac{r-(N-1)\,t+L}{2}} \frac{1}{2^L}. \tag{17}$$

It is important to note that R is not continuous as it is a sum of $L+1$ terms that are each ± 1 with the allowed values being $r \in \{(N-1)\,t - L, (N-1)\,t - L + 2, \cdots, (N-1)\,t + L\}$, i.e. integers spaced by 2. With this, we get the discrete joint distribution

$$p(y, r, t) = \frac{1}{2}\binom{L}{\frac{r-(N-1)t+L}{2}} \frac{1}{2^L}\delta_{y,\text{sgn}(r)}. \tag{18}$$

Now, we can use the formulation of $I_\cap^{\text{sx}}$ derived above given by

$$I_\cap^{\text{sx}} = \sum_{r,t,y} p(r,t,y) \log \frac{p(y \mid t) + p(y, r \mid -t)}{p(y)[1 + p(r \mid -t)]} \tag{19}$$

to find an expression for the redundancy. From Equation 18 follows directly that $p(y) = \frac{1}{2}$ which simplifies the logarithm to

$$\log_2 \frac{2(p(r \mid -t) + p(y \mid t))}{p(r \mid -t) + 1} \tag{20}$$

in all cases of non-zero join probability $p(y, r, t) \neq 0$.

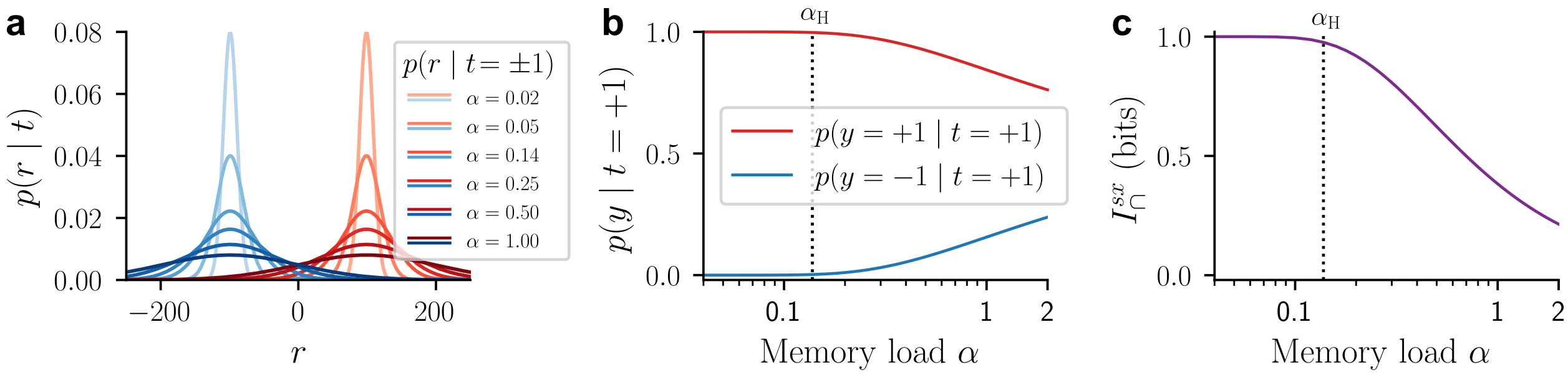

Supplementary Figure 7: **Redundant information between target and recurrent input is high at low memory loads and decreases for larger memory loads. a** Conditional distribution $p(r \mid t \pm 1)$ with $N = 100$ neurons at increasing memory loads α. The two distributions (red: $t = +1$, blue: $t = -1$) are well separated at low α and progressively overlap near the decision boundary $r = 0$ as more patterns are stored. **b** Probability of correct ($y = t$ in red) and incorrect ($y \neq t$ in blue) neuron output as a function of α. Below the Hopfield capacity ($\alpha_{\mathrm{H}} \approx 0.138$) the errors are negligible and increase steadily above it. **c** Analytically calculated redundancy $I_{\cap}^{\mathrm{sx}}(Y : R; T)$. The result agrees with the empirical results reported in the main text.

Due to the symmetry of the neurons we can use $p(r \mid t = +1) = p(-r \mid t = -1)$ to combine the $t = \pm 1$ contributions to derive at

$$\begin{aligned} I_{\cap}^{\mathrm{sx}}(Y : R; T) &= \sum_r p(r \mid t = +1) \log_2 \frac{2\,(p(r \mid t = -1) + p(\mathrm{sgn}(r) \mid t = +1)}{p(r \mid t = -1) + 1} \\ &= \sum_r p(r \mid t = +1) \log_2 \frac{2\,p(r \mid t = -1) + 2 + 2p(\mathrm{sgn}(r) \mid t = +1) - 2}{p(r \mid t = -1) + 1} \\ &= \sum_r p(r \mid t = +1) \log_2 \left(2 + \frac{2\,p(\mathrm{sgn}(r) \mid t = +1) - 2}{p(r \mid t = -1) + 1} \right) \end{aligned} \tag{21}$$

where the sum runs over all allowed values of r as defined above. As the definition of Equation 21 depends on the probability $p(r \mid t)$, which is defined based on a discrete grid spacing, the redundancy depends on both N and m separately.

Equation 17 directly gives us the expression for $p(r \mid t = -1)$ and $p(r \mid t = +1)$ while the probability $p(\mathrm{sgn}(r) \mid t = +1)$ can be written as

$$\begin{aligned} p(\mathrm{sgn}(r) = +1 \mid t = +1) &= \sum_{r \geq 0} p(r \mid t = +1) = \sum_{k=k^*}^{L} \binom{L}{k} \frac{1}{2^L} \\ p(\mathrm{sgn}(r) = -1 \mid t = +1) &= \sum_{r < 0} p(r \mid t = +1) = \sum_{k=0}^{k^*-1} \binom{L}{k} \frac{1}{2^L}, \end{aligned} \tag{22}$$

where k^* is given by $\left\lceil \frac{L-N+1}{2} \right\rceil$ which corresponds to the value of k where r switches sign. The qualitative behavior of the different distributions are shown in Figure 7.

Equation 21 now analytically shows that redundancy is high if the memory load is low: the distributions $p(r \mid t = +1)$ and $p(r \mid t = -1)$ have essentially no overlap (see Figure 7a). So for any r where $p(r \mid t = +1)$ has a significant mass, $p(r \mid t = -1)$ is negligible. At the same time, $p(\mathrm{sgn}(r) \mid t = +1) \approx 1$ since the neuron is almost always correct for low α. On the other hand, if the memory load increases, the probability that the neuron fires correctly $p(\mathrm{sgn}(r) \mid t = +1)$, goes down (see Figure 7b) while the overlap between the $p(r \mid t = +1)$ and $p(r \mid t = -1)$ goes up. This results in a decrease of the redundancy for an increase in α (see Figure 7c). This result supports the empirical result presented in the main text (Section 2.1).

6.4 Robustness of PID Estimation

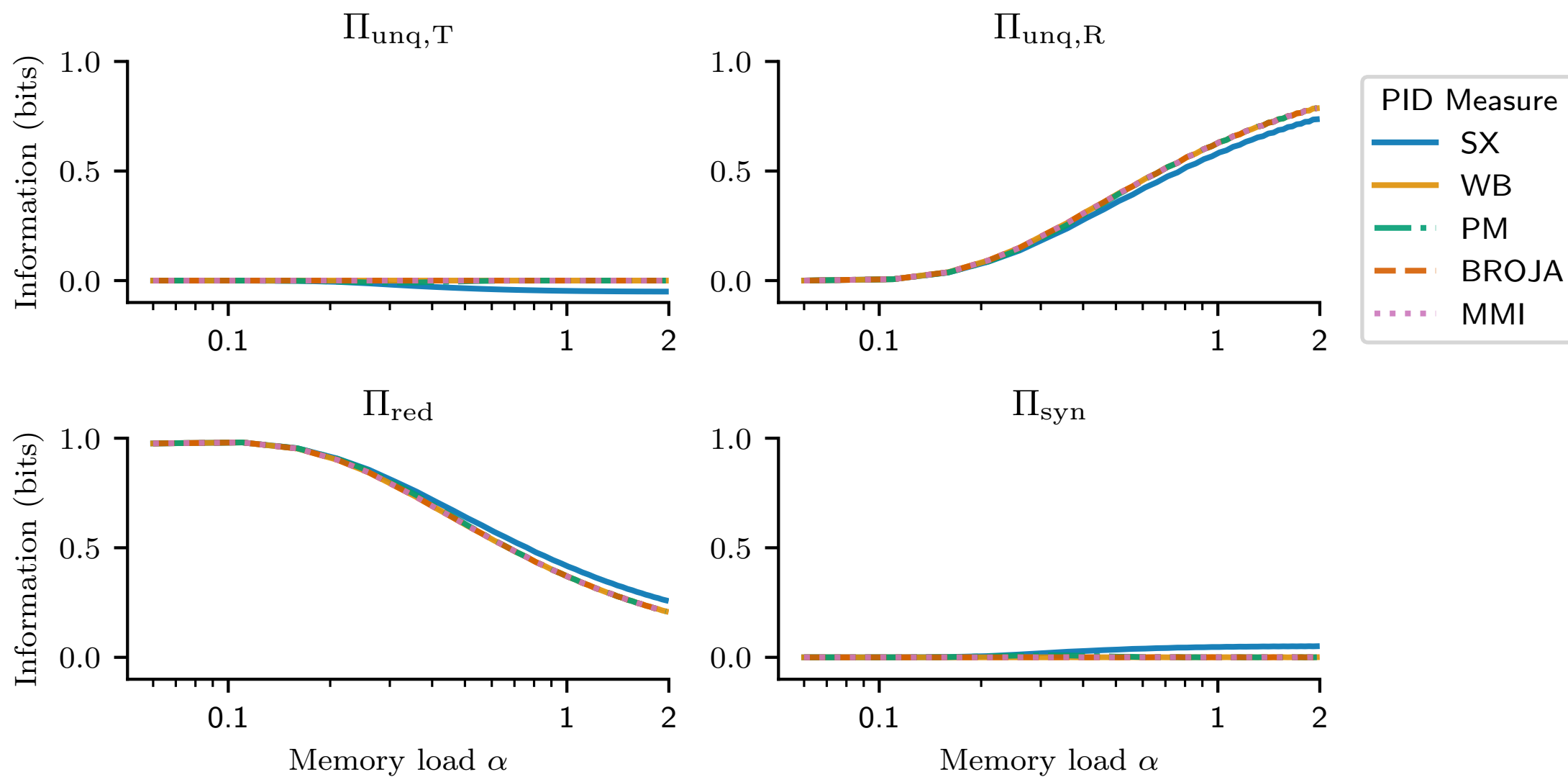

Supplementary Figure 8: **The analysis of the PID profile stays consistent between various different measures of PID.** The four panels show the profile of the different information atoms using various PID measures. While there is a numerical difference between the measures, the qualitative result are consistent. The residual entropy is not included as it is no information atom in the classical sense and does not depend on the choice of PID measure. Curves show the median of 20 network initializations, with values first averaged across all neurons 500 neurons. Shaded areas indicate the central 90% percentile of values.

To validate the empirical results of the analysis of Hebbian Hopfield networks discussed in the main text and the analytical results above, which are based on the $I_\cap^{\mathrm{sx}}$ measure, we performed the same analysis for different PID measures. In particular, we applied $I_\cap^{\mathrm{BROJA}}$ [7], $I_\cap^{\mathrm{min}}$[8], I^{PM}[9] and I^{MMI}[10] (see Figure 8). While there are small numerical differences between the results of the different measures, the overall qualitative results agree with the results based on the $I_\cap^{\mathrm{sx}}$ measure. We used the DIT toolbox [11] to compute all additional measures except $I_\cap^{\mathrm{BROJA}}$, for which we used the optimized implementation provided by Makkeh et al. [12].

As an additional check of the stability of the estimation, we also compare the results for different number of bins used in the dimension of the recurrent input n_r (see Figure 9). As before, the results are not identical which is due to an estimation bias depending on the number of bins. However, the qualitative results are consistent with the main text results. With an increase of n_r, the empirical results approach the analytical results derived on the calculation above. The choice of $n_r = 60$ for our experiments strikes a balance between computational efficiency and estimation accuracy.

Note that in all of the results, the sample size used to estimate the probability distributions is given by $N\alpha$, which is dependent on the memory load α and the number of neurons N. However, the results do not change if we limit the number of samples to be constant for different memory loads.

7 Correlations

The capacity results presented here assume that patterns are created i.i.d. and in particular without spatial correlation. In first experiments, we find that for the redundancy method, more correlation leads to improved capacity (Figure 10a). This is in contrast to Hebbian learning, where the same correlation decreases capacity (Figure 10b).

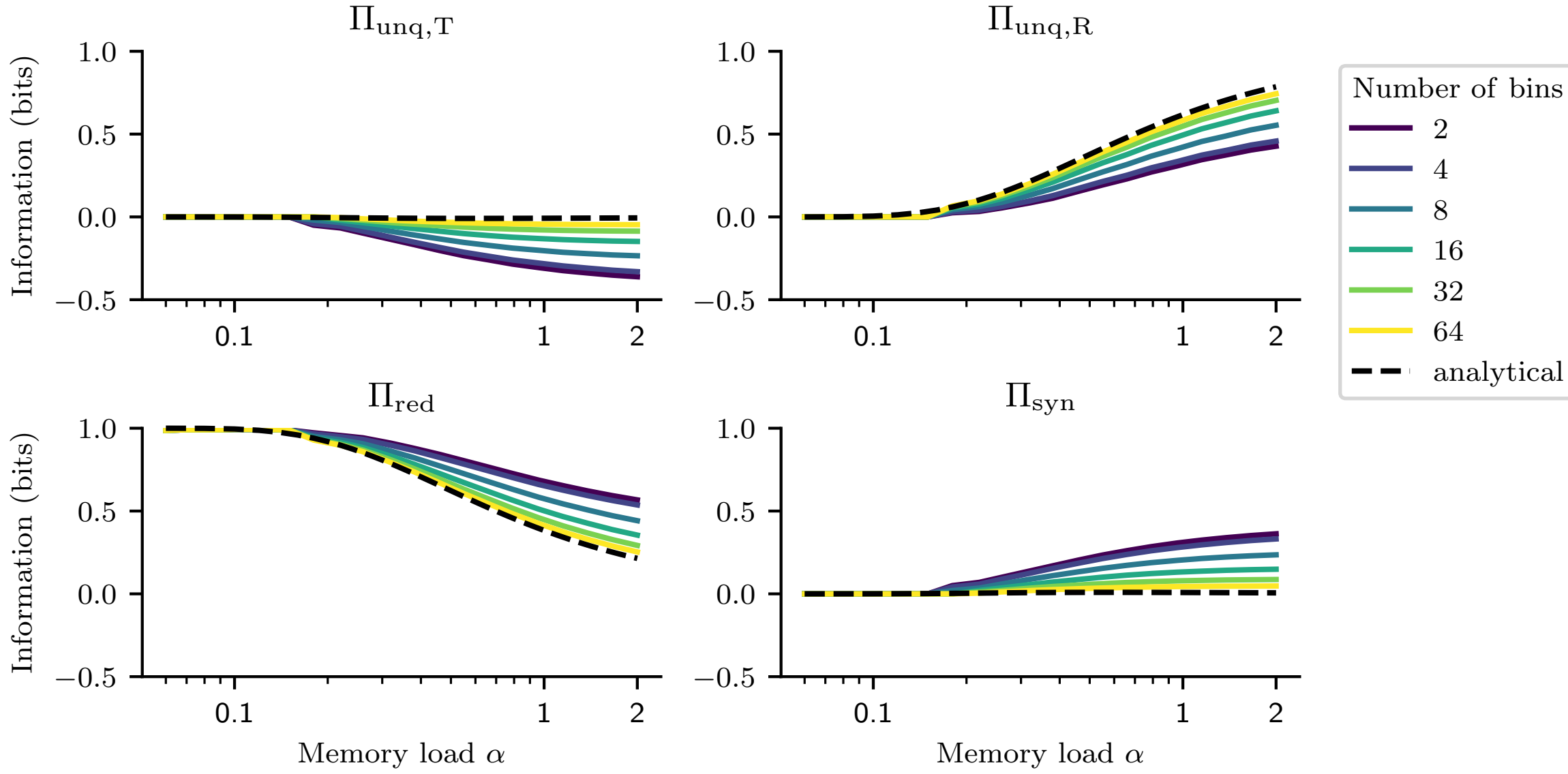

Supplementary Figure 9: **The qualitative results of the PID profile of the Hebbian Hopfield network are consistent between different estimations of probability distributions.** The four panels show the information atoms discussed in the main text Hebbian-learning results.. The different curves show the results for different number of bins used in the dimension of the recurrent input n_r (the standard setting being $n_r = 60$). While the numerical results depend on the number of bins, the qualitative results of an increase of $\Pi_{\text{unq},R}$ together with a decrease of Π_{red} is consistent among all curves. For larger n_r, the results become closer to the analytical results based on the derivation above. Curves show the median of 20 network initializations, with values first averaged across all 500 neurons. Shaded areas indicate the central 90% percentile of values.

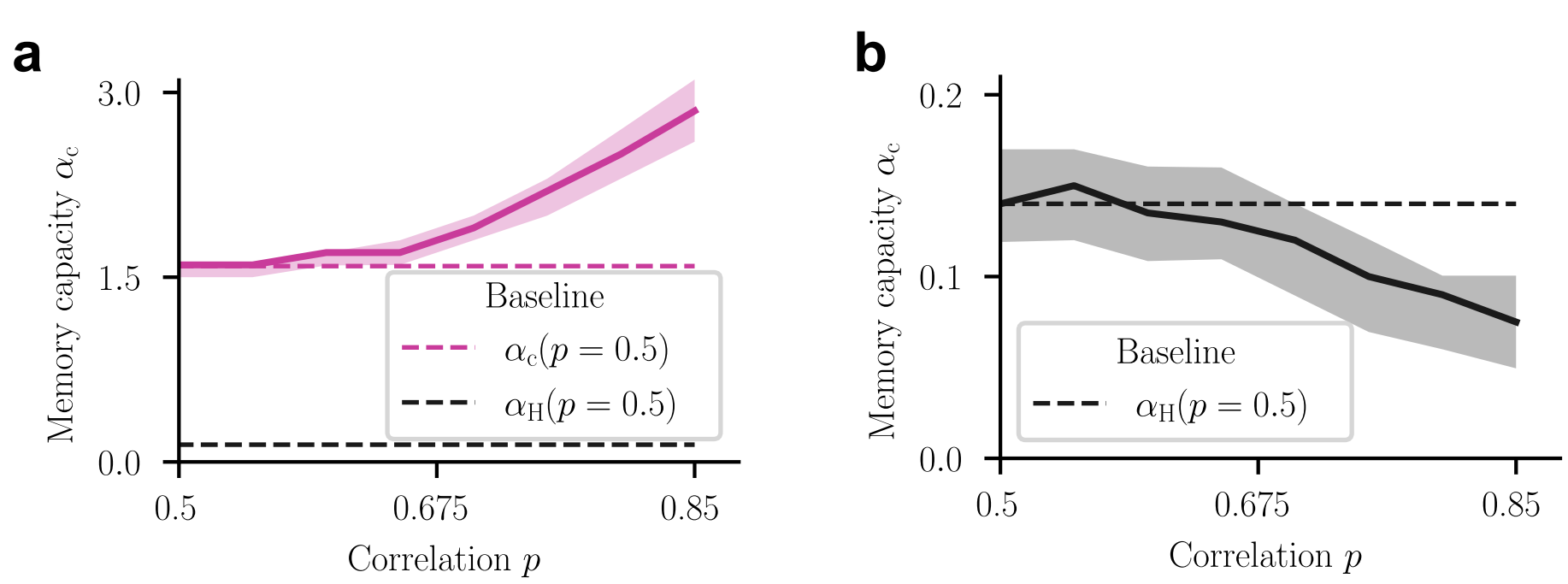

Supplementary Figure 10: **Infomorphic Hopfield networks increase their capacity when trained on spatially correlated patterns. a** When spatial correlations p within patterns are introduced, the memory capacity improves for networks trained on the redundancy goal. **b** Introducing the same correlations as in **a**, the Hebbian rule is able to encode fewer memories. The curves in both panels show the median values across 20 network initializations, and the shaded areas denote the corresponding central 90% percentile.

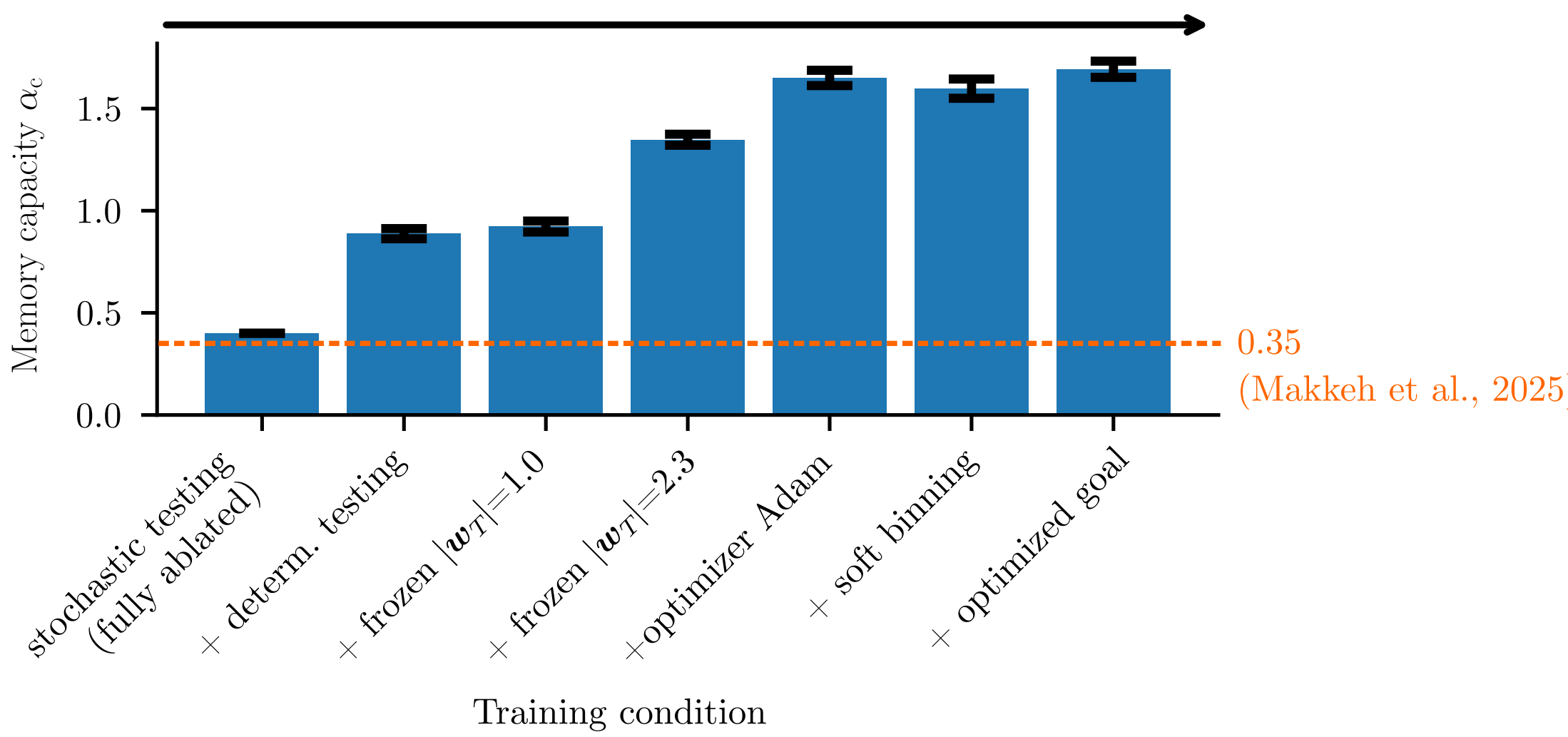

Supplementary Figure 11: **Ablation experiments point to the most effective features of the training procedure for reaching high memory capacity.** In a sequential approach we removed several features of the training procedure for the left-most condition and added them back in one by one. The conditions are (i) fully ablated, (ii) deterministic testing using a Heaviside activation function (vs. stochastic sigmoidal), (iii) frozen target weights $\boldsymbol{w}_T$ (vs. learnable ones), (iv) higher initial scale of target weights (2.3 vs 1.0), (v) improved optimizer for training (Adam vs. SGD), (vi) binning method (differentiable soft vs. hard) and (vii) optimized goal (vs. pure redundancy maximization). The fully ablated condition reaches a capacity comparable to the one reported in [5]. The error bars correspond to the standard deviation over 20 runs.

To create spatial correlations, each pattern $\xi^{(p)}$ is procedurally generated in a stepwise manner, where the probability of flipping the next element of a pattern $p(\xi_{i+1}^{(p)} = 1|\xi_i^{(p)} = -1) = p(\xi_{i+1}^{(p)} = -1|\xi_i^{(p)} = 1)p$ is controlled by the parameter p. The first element of each pattern is chosen randomly with $p(\xi_1^{(p)} = 1) = 0.5$. Increasing p increases the average size of blocks within a pattern without changing the balance of $+1$ and -1 in patterns. While capacity improves for the redundancy method, further investigation could explore other methods of generating correlated patterns, e.g. between patterns, or compare changes in capacity with other learning methods or theoretical bounds.

8 Ablation Experiments

Our infomorphic Hopfield networks build on those introduced in [5], but employ multiple changes to the network and training procedure. Through these changes, the resulting memory capacity increased more than fourfold, from 0.35 to above 1.59. To quantify the effect of the most relevant changes, we conducted a series of ablation experiments. Starting from a 'fully ablated' condition that most closely mirrors the setup of [5], features of the training procedure are successively added in while measuring their effect on memory capacity (Figure 11).

The five features varied in the ablation are:

- **Testing procedure:** stochastic neuron outputs over 20 recall time steps (as in Makkeh et al. [5]) $\rightarrow$ deterministic outputs with up to 100 recall time steps.
- **Target weights $|\boldsymbol{w}_T|$:** learnable, initialized at scale $1.0 \rightarrow$ frozen at scale $1.0 \rightarrow$ frozen at scale 2.3.
- **Optimizer:** SGD $\rightarrow$ Adam.
- **PID estimation:** hard (non-differentiable) binning $\rightarrow$ soft binning (see section 4).

- **Goal function:** $G = \Pi_{\text{red}} \to$ optimized goal $G^{(i)}$ from the main text (Section 3.4).

Out of these, the most effective changes are the deterministic testing procedure, the fixed scale of target weights of 2.3 and the switch to the more sophisticated Adam optimizer.

Further differences to the original were omitted from the ablation study because they were not needed to match the 0.35 capacity reported in [5]. First, the goal function was simplified from $G = \Pi_{\text{red}} + 0.1\, I(Y; R, T)$ in [5] to pure redundancy, $G = \Pi_{\text{red}}$, while retaining high capacity and stable learning. Second, the activation function was simplified from $A(r,t) = \frac{r^8 \operatorname{sign}(r) + t^8 \operatorname{sign}(t)}{r^8 + t^8} \cdot \left(r^8 + t^8\right)^{1/8}$ to the linear form $A(r,t) = r + t$. Additionally, we note that the target weights in [5] differ from our setup in ways beyond initialization scale: there, they are drawn from a normal distribution (with both positive and negative values). The Hopfield layer was also followed by a simple decoder layer to compensate for inverse neuron representations. These structural differences are outside the scope of the ablation.

References

[1] Abdullah Makkeh, Aaron J Gutknecht, and Michael Wibral. Introducing a differentiable measure of pointwise shared information. *Physical Review E*, 103(3):032149, 2021.

[2] Pavel Tolmachev and Jonathan H. Manton. New insights on learning rules for Hopfield networks: Memory and objective function minimisation. In *2020 International Joint Conference on Neural Networks (IJCNN)*, page 1–8. IEEE, July 2020. doi:10.1109/ijcnn48605.2020.9207405. URL `http://dx.doi.org/10.1109/IJCNN48605.2020.9207405`.

[3] David A Ehrlich, Kyle Schick-Poland, Abdullah Makkeh, Felix Lanfermann, Patricia Wollstadt, and Michael Wibral. Partial information decomposition for continuous variables based on shared exclusions: Analytical formulation and estimation. *Physical Review E*, 110(1):014115, 2024.

[4] Jim Kay. Information-theoretic neural networks for unsupervised learning: mathematical and statistical considerations. Technical Report 1573387449478062080, Scottish Agricultural Statistics Service, 1994.

[5] Abdullah Makkeh, Marcel Graetz, Andreas C. Schneider, David A. Ehrlich, Viola Priesemann, and Michael Wibral. A general framework for interpretable neural learning based on local information-theoretic goal functions. *Proceedings of the National Academy of Sciences*, 122(10):e2408125122, 2025. doi:10.1073/pnas.2408125122. URL `https://www.pnas.org/doi/abs/10.1073/pnas.2408125122`.

[6] Robert J. McEliece, Edward C. Posner, Eugene R. Rodemich, and Santosh S. Venkatesh. The capacity of the Hopfield associative memory. *IEEE Trans. Inf. Theory*, 33:461–482, 1987. URL `https://api.semanticscholar.org/CorpusID:14340808`.

[7] Nils Bertschinger, Johannes Rauh, Eckehard Olbrich, Jürgen Jost, and Nihat Ay. Quantifying unique information. *Entropy*, 16(4):2161–2183, 2014.

[8] Paul L Williams and Randall D Beer. Nonnegative decomposition of multivariate information. *arXiv preprint arXiv:1004.2515*, 2010.

[9] Conor Finn and Joseph T Lizier. Pointwise partial information decompositionusing the specificity and ambiguity lattices. *Entropy*, 20(4):297, 2018.

[10] Nils Bertschinger, Johannes Rauh, Eckehard Olbrich, and Jürgen Jost. Shared information—new insights and problems in decomposing information in complex systems. In Thomas Gilbert, Markus Kirkilionis, and Gregoire Nicolis, editors, *Proceedings of the European Conference on Complex Systems 2012*, pages 251–269, Cham, 2013. Springer International Publishing. ISBN 978-3-319-00395-5.

[11] R. G. James, C. J. Ellison, and J. P. Crutchfield. dit: a Python package for discrete information theory. *The Journal of Open Source Software*, 3(25):738, 2018. doi:https://doi.org/10.21105/joss.00738.

[12] Abdullah Makkeh, Dirk Oliver Theis, and Raul Vicente. Broja-2pid: A robust estimator for bertschinger et al.'s bivariate partial information decomposition. *Entropy*, 20(4):271, 2018.
