# Supplementary material for: Redundancy maximization as a principle of associative memory learning in Hopfield networks
Source: Nat Commun. 2026 Nov 6;17:11499. doi: 10.1038/s41467-026-78777-2 (PMC13743752; doi:10.1038/s41467-026-78777-2)
Supplement: Supplementary file 2 [file 41467_2026_78777_MOESM2_ESM.pdf]

nature portfolio

Peer Review File

Redundancy Maximization as a Principle of Associative Memory Learning in Hopfield Networks

Corresponding Author: Professor Viola Priesemann

This file contains all reviewer reports in order by version, followed by all author rebuttals in order by version.

Version 0:

Reviewer comments:

Reviewer #1

(Remarks to the Author)
Contributions:

Based on the partial information decomposition or PID framework, this manuscript studies the role of the local redundant information (between the node input and the target output) in learning the weights of an associative memory network. First, the manuscript provides empirical evidence that this redundant information is high in a classical associative memory network (below memory capacity) where the weights are learned using the standard Hebbian rule. Using this as a motivation, the manuscript proposes the learning of weights by explicitly maximizing the redundant information, and demonstrates that this increases the memory capacity. Furthermore, more empirical results are provided to highlight how maximization of mutual information results in similar memory capacity, and performing a hyperparameter optimization of the different PID term weights (in a weighted sum objective) can further improve the memory capacity. Finally, comparison to other weight learning schemes highlight the improved memory capacity of the redundancy maximizing weight learning.

Novel & significance:

First, it is not clear how novel or significant is the main thesis of this manuscript that the goal of any (supervised) learning is to the maximize the redundant information between the input and the target output. This was already presented in [13] for both supervised learning, and associative memory learning. If redundancy maximization between input and output is the main contribution, the novelty is somewhat limited.

One potential novel aspect is what is listed in lines 48-50 as "an information-theoretic analysis of a classical Hebbian Hopfield network which reveals that redundancy between recurrent input and target (teaching signal) dominates the neurons' output when operating below capacity". However, the validation of this claim is somewhat limited:
- Figure 1D is solely an empirical observation and relies on how the redundancy and other information related terms are computed. Relying on single method of empirical evaluation of these terms, such as I_{cap}^{sx}, does not necessarily imply that the dependencies we see are not an effect of the (inaccuracies in the) empirical estimates. In fact, as shown in Appendix E, for small values of N (implying less reliable estimates of information-theoretic quantities), the high redundancy shown in Figure 1D at below capacity does not hold. This makes it unclear whether the high redundancy of the Hebbian weights (below capacity) is due to true underlying connection or an artifact of the measurement quality.
- Given that the information-theoretic quantities such as redundancy are quite formally defined, this would be a more significant contribution if there was a theoretical (or at least more technical) intuition of why Hebbian weights have high redundancy. This is an expected result as the Hebbian weights are computed using the targets, but a formal connection would be of interest.
- Finally, it is unclear how robust the estimated redundancy of the Hebbian weights are as it is not clear how the necessary w_T, the weights for the target output, are obtained as they are not part of the Hebbian learning. It seems as per Appendix A that w_T is never learned in any of the experiments, and set to 2.3. If this is the value used to compute the redundancy between the target and input in a network with Hebbian weights, it would be important to understand how this value affects the presented results in Figure 1D (and elsewhere).

Focusing on the redundancy maximizing weight learning scheme, the novelty is already limited given that Makkeh et al. [13] had already considered this. Authors say that they are "building on and improving the proof of concept provided by Makkeh

et al. [13]", and explain in lines 226-231 that they are using a different activation scheme and binning procedure (to compute the information-theoretic quantities). However, there are some concerns here:
- First, it is not clear why these modifications should lead to such a significant increase in the memory capacity from the 0.35 reported in [13] to ~1.6 in this manuscript. As noted by the authors, this is a very significant fourfold increase, and warrants a discussion (with theoretical intution and ablations) of why such an increase in capacity was obtained. Without this intuition, it is not clear what is it that is leading to the increase in capacity as both schemes are already maximizing redundancy.
- Second, since these capacities are empirically calculated (without any theoretical evaluations), it is not clear if such a high memory capacity will be obtained across varying values for the number of neurons N --- does increasing the number of neurons increase or decrease the capacity? Would the "finite size effects" reported in Appendix E imply that the capacity increases with N as the redundancy estimate gets better?
- Another confusing aspect of the results in Figure 2, especially Figures 2C,D, is that, while maximizing redundancy seems to increase memory capacity, it is unclear why the accuracy drops at \alpha_c^{red} = ~1.6 when the redundancy continues to be almost as high as possible (close to 1.0). While the authors briefly discuss this in lines 145-147, I think this warrants a more technical explanation. To me, this result seems to imply that high redundancy does not necessarily imply high accuracy. Since the ~1.6 is not a theoretical capacity limit, it is just not clear up until which point would high redundancy and high accuracy coincide, and when do high redundancy start co-occurring with low accuracy. If redundancy is the underlying mechanism, the slight drop in redundancy should only translate to a slight drop in accuracy, not a sharp drop as seen in Figure 2C,D. Thus, this result seems counter-intuitive to me.
- Finally, it would be good to show the computational complexity of the redundancy maximizing weight learning related to the Hebbian learning scheme, or other baselines. While I understand that the PID based schemes would be expensive and not scalable as it requires the estimation of joint probabilities, it would be good to get a sense of the kind of computation we would need to spend relative to existing schemes.

Regarding the analysis comparing redundancy maximization to mutual information maximization, the analysis needs to be further clarified:
- First, it would be good to understand why we only consider the input-target mutual information I(Y:T) and co-information I(Y:R:T), and not something like the I(Y:R,T)?
- In the maximization of G = I(Y:T), it is not clear why the redundancy \Pi_{red} starts dropping beyond the capacity in Figure 3C, but it does not with G = \Pi_{red} (as seen in Figure 2D). As there are weight configurations with high redundancy (as in Fig 2D), shouldn't maximizing I(Y:T) = \Pi_{red} + \Pi_{unq, T} pick configurations where \Pi_{red} continues to be large beyond the \alpha_c^{red} as these configurations would have higher I(Y:T). The only setup where this would not be true is if the unique information \Pi_{unq, T} goes dramatically negative. However, this situation is very odd as one should not expect negative unique information.
- Thus, the justification for claiming that maximizing mutual information implicitly maximizes redundancy seems insufficient. Furthermore, it is important to understand what happens at the threshold \alpha_c^{red} that I(Y:I) starts diverging from \Pi_{red} (although, to me it seems like they should not as per my above comment about the optimization).
- In fact, if redundancy maximization is truly sufficient, the capacity should not be affected at all by $\gamma_{unq, T}$ in Figure 3D as we are maximizing the redundancy in all cases. However, the results show that negative values of $\gamma_{unq, T}$ (implying also minimizing \Pi_{unq, T}) actually hurts capacity.

The analysis using hyperparameter optimization for the goal parameters is very interesting. However, the results highlight the lack of interpretability in the results (which is counter to the claims of interpretability in the PID framework). For the optimal hyperparameters in Figure 4C,D, it is odd that \gamma_{unq,T} is both positive and negative, implying that we can either maximize \Pi_{unq,T} or minimize it, which makes the decomposition uninterpretable. This is also counter to the results shown in Figure 3D, which implied that negative \gamma_{unq,T} leads to worse capacity, and increasing it does not affect capacity significantly. Thus, it is unclear how we can square the resuts in Figure 3D with the hyperparameter optimization results in Figure 4C,D.

Minor typographical error:

- In line 201, I think the authors wish to refer to Figure 5C, not Figure 5B.

Conclusion:

Overall, in this manuscript, the authors put forward an intuitive, though somewhat expected and known as per [13], objective for learning weights in the classical associative memory network. However, the objective itself requires the hard estimation of joint probabilities, and the current manuscript does not discuss how the estimation scheme and quality affects the results in this manuscript. Furthermore, the results are empirical in nature, and are limited to a small number of configurations of associative memory networks, making it unclear whether these results are robust and significant in general.

(Remarks on code availability)

Reviewer #2

(Remarks to the Author)

In this paper, Blumel et al., use the partial information decomposition to interrogate the dynamics of associative memory in Hopfield networks, finding that Hebbian learning seems to be driven by redundancy, and apparently compromised by unique and synergistic interactions. They then go on to show that an "infomorphic neuron", trained to maximize redundant information (or some linear combination of partial information atoms) can achieve very high performances on an associative learning task - comparable with current state of the art.

In general, I am a big fan of this paper. I think this is an excellent application of PID to a concrete and well-defined problem. I am also very enthusiastic about the use of information-theoretic objective functions. So much of information theory in complex systems stops at the level of descriptive statistics (e.g. neuroscience studies that ask "is there more synergy in an awake brain than an unconscious one"), and it is exciting to see a new spin on these methods.

In general the methodology seems strong and the results robust. I have some small technical quibbles, but they can be addressed with minor revisions.

One thing that gives me pause is that the authors also try to maximize the co-information, and find that no patterns are stored. I cannot find any place in the paper where the definition of co-information is given, so I will assume that here the definition is the same as the original Bell paper [1], which is positive when redundant interactions dominate, and negative when synergistic interactions dominate. Later, Williams and Beer [2] showed that, for two sources X_1, X_2 and one target Y, the co-information is equivalent to Red({1}{2} ; Y) - Syn({1,2} ; Y). Based on this identity, I find it a little concerning that optimizing the co-information apparently does not perform at all, even when the i_{sx} redundancy function does. If it were the case that redundancy generally improves performance, then naively, I would expect optimizing the co-information to perform equivalently well, especially since the goal function $G^{(i)}$ has a very co-information-like form (positively weighting redundancy, negatively weighting synergy).

As it stands, I don't know what to make of this apparent inconsistency. Are the authors sure that the co-information was implemented correctly? The literature on co-information is messy, sometimes co-information is used to refer to the inverse-signed measure, which is Syn({1,2) ; Y) - Red({1}{2} ; Y).

Another small thing is that I'd like to see a cartoon that describes Hopfield networks a little more intuitively. Fig. 1A kind of starts, but it's still a little abstract. Is there a visualization that describes the entire process up updating (incluing intuitive representations of equations like Eqs. 1 and 2? I think this would go a long way towards making the paper more accessible.

All in all, I commend the authors for an impressive piece of work.

[1] Bell, A. J. (2003, April). The co-information lattice. 4th International Symposium on Independent Component Analysis and Blind Signal Separation, Nara, Japan.
[2] Williams, P. L., & Beer, R. D. (2010). Nonnegative Decomposition of Multivariate Information. arXiv:1004.2515

(Remarks on code availability)

Reviewer #3

(Remarks to the Author)
The authors provide an analysis of Hopfield networks based on partial information decomposition (PID). The authors find empirically that high redundancy is correlated with high recall accuracy in Hopfield networks, but is not strongly correlated with other components used in the PID profile. This is used as motivation to optimize for redundancy. Building on previous work on infomorphic networks, they design a type of Hopfield network that optimizes the informorphic objective, which is composed of a weighted sum of PID components. They run multiple experiments that test various weightings of the infomorphic objective, including hyper-parameter sweeps, and observe that redundancy is the most important component for associative memory recall accuracy. They also show that their objective leads to better accuracy than other SOTA objectives at high memory loads.

The authors perform an insightful and novel analysis of Hopfield networks based on PID, and they use this analysis to improve performance of auto-associative networks over SOTA methods. The paper is well written, the diagrams provide useful explanations of concepts and experiments, and the experiments are logical.

Below I provide suggestions for making the paper a bit clearer for those new to PID, and how to strengthen the empirical results and explanation of the results.

For equations 3 and 4, I believe it would be helpful to have a table, possibly in the appendix, that explicitly defines the pi terms (pi_{syn}, pi_{red}, etc.). These are nicely visualized in Figure 1C, but I can’t seem to find an explicit mathematical definition in the paper. They can be inferred from equations 3,4, and the diagrams, but an explicit definition would be nice. If these definitions do exist in the paper, could the authors make sure there is a clear reference to it? Otherwise, could the authors spell these definitions out somewhere?

From what I could tell, the authors ran all of their tests from section 3.2 onwards with networks of size 100 neurons, and section 3.1 experiments with networks size 500 neurons. Is this correct? If so, could the authors also run tests on other sized networks? I believe it is important the authors establish that their empirical results generalize to any reasonably sized

network. For example, maybe they could test at [50, 100, 200, 400, 800] neurons to see if there are any clear trends as they double the size of the network. Maybe doing this for the tests in figure 2 and figure 5 would be sufficient. Also, showing the findings from section 3.1 generalize to at least one more network size would be useful too. These extra results can then be placed in the appendix and noted in the main text.

Would it be feasible for the authors to test a version of their memory network on continuous valued vectors, e.g., vectors with values between 0 and 1 or between -1 and 1? I suspect this may best be left to future work, but I was curious what the authors' thinking is about this.

The authors provide empirical motivation for optimizing for redundancy for auto-associative memory. However, I think the paper lacks a clear mathematical intuition for why this should be the case. For example, I do not see an attempt in the discussion section to provide mathematical intuition for why, for example, redundancy should be more important than mutual information or other similar measures when training auto-associative memory units. Do the authors believe there is good mathematical intuition for why we see the empirical results we do? Is there reason to believe this empirical result is not somehow unique to the particular experiments ran in the paper (e.g., with binary vectors, at 100 neurons, etc.) and generalizes beyond it? If so, what is this explanation?

(Remarks on code availability)

Version 1:

Reviewer comments:

Reviewer #1

(Remarks to the Author)
I would like to first thank the authors for addressing all my comments in a thorough professional manner. I really enjoyed reading the updated version and the author responses. I appreciate the detailed clarifications to my various questions regarding the effect of the different weighted sum objective for infomorphic learning and how to appropriately interpret the results. The new results covering the scaling of the number of memories (Appendix E), the analysis of the training runtimes (Figure 20), and the effect (or rather lack thereof) of changing the PID term estimation schemes and parameters (Appendix F.4) clearly highlight the robustness of the trends initially seen on smaller experiments.

I also appreciate the proper positioning with respect to Makkeh et al [13], highlighting the role of redundancy maximization in associative memory learning. The new analysis in F.1 provides a concrete connection with the geometric separation (necessary for recall in Hopfield models) and the maximal redundancy achieved when the separation holds. Analysis in F.3 studies the Hebbian learning, showing how the available gap between the signal and the noise terms (at different memory loads) translates to the separation condition, which in turn leads to insights regarding the redundancy (loads when it is almost maximal vs loads where redundancy starts to drop, matching the existing empirical evaluations). In my opinion, these results provide a more thorough connection between redundancy maximization and associative memory learning.

I also appreciate the ablation experiments in Appendix H demonstrating the different additions on top of Makkeh et al [13], and how it affects capacity. However, the results are a bit counterintuitive. Based on Figure 17, it appears that the major capacity jumps come from (i) deterministic testing for (5x) longer number of recall time steps, (ii) modifying the frozen w_T value from 1.0 to 2.3, and (iii) switching the optimizer from SGD to Adam. Jump (i) implies that one just needs to take the model learned from Makkeh et al [13] and iterate it for longer recall timesteps with appropriate nonlinear activation (as in the standard Hopfield model). The jump (ii) indicates, somewhat counterintuitively, that freezing the w_T parameters (instead of learning it from data) and manually setting it to 2.3 is much better than trying to learn the w_T value. Jump (iii) indicates that we had the right learning objective, but we were not optimizing it appropriately (or correspondingly, the SGD hyperparameters might not have been sufficiently tuned). All these are interesting additions, but appear more like engineering steps, and do not seem to align with the main contributions of this submission on top of Makkeh et al [13] such as the role of redundancy and the weight objective. It is possible that I am misunderstanding something, but I find these ablation results separating this current submission from prior art a bit limiting.

Finally, this w_T parameter appears to be a critical hyperparameter in terms of capacity. Thus it is odd to see coarse-grain evaluation of this parameter for hebbian learning in the set w_T \in { 10^{-10}, 1, 10, 100} in Figure 18, whereas a much more fine-grained values around [0,10] is considered for infomorphic learning in Figure 19. If the coarse-grain grid (from Hebbian learning) was used, the whole range of [2,5] where memory capacity is above 1 is completely lost. It is also not clear why learning the w_T is so detrimental; it appears better than Hebbian but quite a drastic drop from the fixed w_T setting. Also, given the scaling experiments in Appendix E, the interaction of this w_T and the number of neurons and memories is not well understood. This critical role of a somewhat underdiscussed hyperparameter undercuts the main contribution of this submission.

(Remarks on code availability)

Reviewer #2

(Remarks to the Author)
The paper can be published as-is. I commend the team for an excellent piece of work.

(Remarks on code availability)

Open Access This Peer Review File is licensed under a Creative Commons Attribution 4.0 International License, which permits use, sharing, adaptation, distribution and reproduction in any medium or format, as long as you give appropriate credit to the original author(s) and the source, provide a link to the Creative Commons license, and indicate if changes were made.
In cases where reviewers are anonymous, credit should be given to 'Anonymous Referee' and the source.
The images or other third party material in this Peer Review File are included in the article’s Creative Commons license, unless indicated otherwise in a credit line to the material. If material is not included in the article’s Creative Commons license and your intended use is not permitted by statutory regulation or exceeds the permitted use, you will need to obtain permission directly from the copyright holder.
To view a copy of this license, visit https://creativecommons.org/licenses/by/4.0/

Response to Reviewers

We would like to thank the reviewers for their careful and constructive feedback. In response, we made substantial additions to the manuscript, including: (i) a new introductory figure illustrating Hopfield network retrieval process, (ii) a formal analysis of why Hebbian learning produces high redundancy, with both an analytical derivation and an intuitive visualization (new Appendix F), (iii) validation of our PID analysis using alternative measures and estimation parameters (new Appendix F.4), (iv) ablation experiments quantifying each methodological improvement over prior work (new Appendix H), and (v) scaling experiments across networks size (new Appendix E). Below we provide a detailed point-by-point response to each of the reviewers' comments. Modifications in the manuscript are highlighted in red.

Reviewer 1

Contributions

Based on the partial information decomposition or PID framework, this manuscript studies the role of the local redundant information (between the node input and the target output) in learning the weights of an associative memory network. First, the manuscript provides empirical evidence that this redundant information is high in a classical associative memory network (below memory capacity) where the weights are learned using the standard Hebbian rule. Using this as a motivation, the manuscript proposes the learning of weights by explicitly maximizing the redundant information, and demonstrates that this increases the memory capacity. Furthermore, more empirical results are provided to highlight how maximization of mutual information results in similar memory capacity, and performing a hyperparameter optimization of the different PID term weights (in a weighted sum objective) can further improve the memory capacity. Finally, comparison to other weight learning schemes highlight the improved memory capacity of the redundancy maximizing weight learning.

We thank the reviewer for their thorough and constructive review. In the following, we address each of the raised points individually.

Novel & significance

First, it is not clear how novel or significant is the main thesis of this manuscript that the goal of any (supervised) learning is to the maximize the redundant information between the input and the target output. This was already presented in [13] for both supervised learning, and associative memory learning. If redundancy maximization between input and output is the main contribution, the novelty is somewhat limited.

Makkeh et al. [13] provided proof of concept that redundancy maximization can be sufficient for associative memory learning with limited performance. To achieve this, they made a series of unconventional model choices, such as adding a separate readout layer, ignoring part of the gradients due to a non-differentiable binning procedure, choosing a tailored activation function and adding other information atoms besides redundancy in their goal function for stability reasons.

In this work, we significantly expand on this idea in three major ways. First, we show that redundancy is also increased by Hebbian learning for associate memory networks, providing evidence that the seemingly ad-hoc goal function chosen by Makkeh et al. [13] might in fact point to a more fundamental underlying principle. Second, by incorporating important improvements and simplifications to the infomorphic Hopfield framework which address the above-mentioned issues, we show that the associative memory performance of redundancy maximization matches or even exceeds state-of-the-art learning rules. To demonstrate the impact of each of these improvements, we have added an ablation study

to Appendix H (Figure 17). Third, we demonstrate via hyperparameter optimization that in addition to maximizing redundancy, suppressing other information terms can further improve the memory capacity.

Overall, through the analysis of redundancy in Hebbian networks, state-of-the-art performance and hyperparameter optimization, this work provides a comprehensive understanding of associative memory learning through the principle of redundancy maximization.

> One potential novel aspect is what is listed in lines 48-50 as "an information-theoretic analysis of a classical Hebbian Hopfield network which reveals that redundancy between recurrent input and target (teaching signal) dominates the neurons' output when operating below capacity". However, the validation of this claim is somewhat limited:
> - Figure 1D is solely an empirical observation and relies on how the redundancy and other information related terms are computed. Relying on single method of empirical evaluation of these terms, such as I^{sx}_{cap}, does not necessarily imply that the dependencies we see are not an effect of the (inaccuracies in the) empirical estimates. In fact, as shown in Appendix E, for small values of N (implying less reliable estimates of information-theoretic quantities), the high redundancy shown in Figure 1D at below capacity does not hold. This makes it unclear whether the high redundancy of the Hebbian weights (below capacity) is due to true underlying connection or an artifact of the measurement quality.

We agree that the estimation of information quantities can be challenging, and that our results could benefit from additional validations of our methodology. To this end, we have added the following validations: First, we analyzed the Hebbian Hopfield networks using other PID measures, to ensure that the observed trends are not due to the idiosyncrasies of the chosen $I^{\mathrm{sx}}_{\cap}$ PID measure (see Figure 14). Second, we reran our analysis with varied estimation parameters, such as different numbers of bins in the estimation of the probability distributions (see Figure 15). We find that these variations produce qualitatively identical results, and have added an appendix section (Appendix F.4) where we discuss the results for those comparisons in detail. Additionally, we also added an analytical derivation of redundancy in Hebbian Hopfield networks (Appendix F.3). While the derivation uses the definition of $I^{\mathrm{sx}}_{\cap}$, it is independent of any binning assumptions and shows the same behavior as our empirical results.

Note, however, that the finite size effects discussed in Appendix E are not related to the estimation method, but point to a general limitation of information theory. For small number of patterns (necessary for studying low α in small networks), by chance specific elements of the target pattern can be constant over all patterns, resulting in vanishing entropy. With the entropy providing an upper bound to redundancy, there can accordingly also be no redundancy in these situations, rendering a PID analysis futile. To further clarify this effect we expanded Appendix E.

> - Given that the information-theoretic quantities such as redundancy are quite formally defined, this would be a more significant contribution if there was a theoretical (or at least more technical) intuition of why Hebbian weights have high redundancy. This is an expected result as the Hebbian weights are computed using the targets, but a formal connection would be of interest.

We thank the reviewer for this suggestion. As already mentioned, we added a new appendix section (Appendix F.3) that provides an analytical derivation of redundancy in Hebbian Hopfield networks based on the definition of $I^{\mathrm{sx}}_{\cap}$. We also added more technical intuition, based on the separation of the conditional input distributions $p(R \mid T = +1)$ and $p(R \mid T = -1)$ and the properties of the pointwise $I^{\mathrm{sx}}_{\cap}$ (Appendix F.1). To support this part, we also included new figures (Figures 9-11). We have placed this section in the appendix rather than the main text, as the formal treatment involves details of the $I^{\mathrm{sx}}_{\cap}$ measure that may be too technical for the main text.

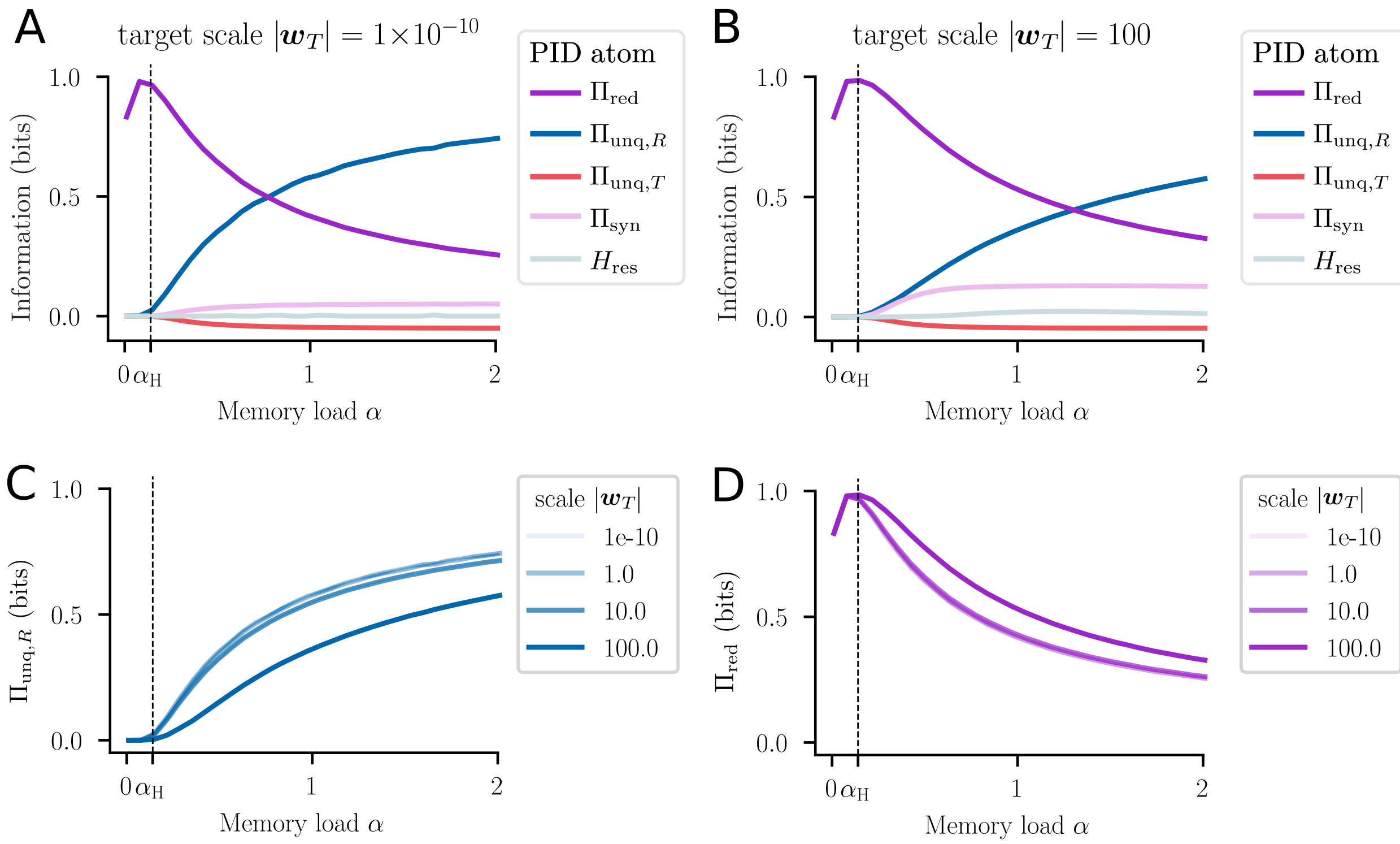

Figure 18: **Increasing the target weights w_T does not qualitatively change the transition in redundancy for Hebbian learning.** **A:** The PID profile for Hebbian learning as depicted in Fig. 2D. The target weights are set to $\boldsymbol{w}_T = 1 \cdot 10^{-10}$. Above the memory capacity, falling redundancy is replaced with recurrent unique $\Pi_{\text{unq},R}$. **B:** The PID profile is reproduced with target weights $w_T = 100$ now dominating the activity of the network. Above the memory capacity, falling redundancy is replaced with both recurrent unique $\Pi_{\text{unq},R}$ and synergistic information Π_{syn}. **C-D:** The changes in unique recurrent information $\Pi_{\text{unq},R}$ (Panel **C**) and Π_{red} are shown as a function of target weights $|\boldsymbol{w}_T|$. For larger target weights there is a relative drop in recurrent unique information and an increase in redundancy at high memory loads. The memory load α at which the transition in redundancy occurs is not affected by the scale.

- Finally, it is unclear how robust the estimated redundancy of the Hebbian weights are as it is not clear how the necessary w_T, the weights for the target output, are obtained as they are not part of the Hebbian learning. It seems as per Appendix A that w_T is never learned in any of the experiments, and set to 2.3. If this is the value used to compute the redundancy between the target and input in a network with Hebbian weights, it would be important to understand how this value affects the presented results in Figure 1D (and elsewhere).

We thank the reviewer for this valuable comment. Indeed, the target weights $\boldsymbol{w}_T$ are set to 1×10^{-10} for our analysis of Hebbian networks to reflect the lack of a direct target input in conventional Hebbian Hopfield networks. We have included this setting in Table 1 now. Additional experiments show that the PID profile obtained in Figure 2 is robust with respect to changes in $\boldsymbol{w}_T$ (see Figure 18), unless the target input becomes too large (and thus driving). Here, we varied the scale of target weights from 1×10^{-10} to 100, while keeping the recurrent weights $\boldsymbol{w}_R$ fixed at the value obtained from outer-product learning. As the target weights become driving, the transition in PID atoms still occurs around the Hebbian capacity α_H. At the same time, the increase in unique recurrent information $\Pi_{\text{unq,R}}$ becomes smaller, while there is less loss of redundancy Π_{red} and some gain in synergy Π_{syn}.

When considering infomorphic Hopfield networks actively trained on redundancy, the scale of target weights $\boldsymbol{w}_t$ does have an impact on the memory capacity (see also Fig. 17). As can be seen in Fig. 17, we chose the value of 2.3 from a range of values that maximize capacity. However, even if this scale is not set optimally or if the target weights are learned during training, high capacities far exceeding α_H are still attainable.

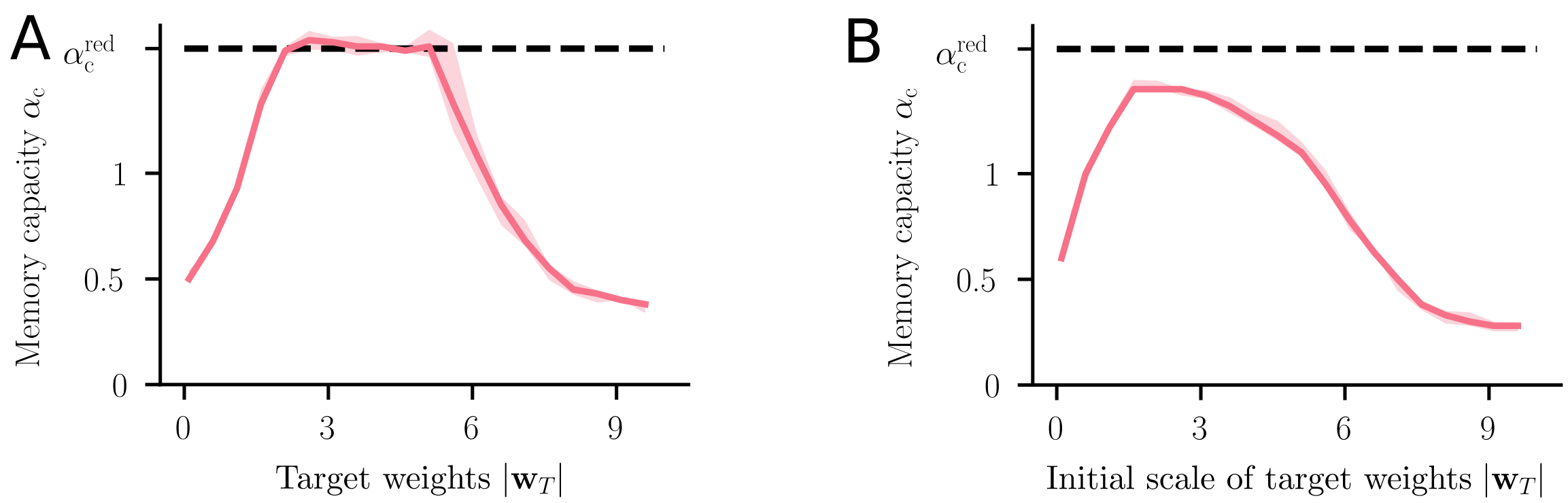

Figure 19: **The memory capacity of infomorphic Hopfield networks exceeds the Hebbian baseline even for far-from-optimal target weight settings. A:** Memory capacity α_c is shown as a function of scale of target weights $|\boldsymbol{w}_T|$. As for most experiments, here the target weights are frozen (not learnable) during training and the networks are trained with redundancy as a goal. There is a wide range of scales $|\boldsymbol{w}_T| \in (2.2, 5)$ for which the measured capacity plateaus. **B:** Same as panel A, but the target weights $\boldsymbol{w}_T$ are also learned during training.

Focusing on the redundancy maximizing weight learning scheme, the novelty is already limited given that Makkeh et al. [13] had already considered this. Authors say that they are "building on and improving the proof of concept provided by Makkeh et al. [13]", and explain in lines 226-231 that they are using a different activation scheme and binning procedure (to compute the information-theoretic quantities). However, there are some concerns here:

- First, it is not clear why these modifications should lead to such a significant increase in the memory capacity from the 0.35 reported in [13] to 1.6 in this manuscript. As noted by the authors, this is a very significant fourfold increase, and warrants a discussion (with theoretical intution and ablations) of why such an increase in capacity was obtained. Without this intuition, it is not clear what is it that is leading to the increase in capacity as both schemes are already maximizing redundancy.

We agree with the reviewer that the significant improvements over the results by Makkeh et al. [13] should be better highlighted with their individual impact on task performance clearly assessed. To this end, we have conducted ablation experiments, in which we successively remove the different optimizations to demonstrate their respective impact.

While Makkeh et al. [13] performed an initial investigation revealing that redundancy maximization can serve as a local goal to implement associative memories, the present work aims to test the hypothesis that redundancy is indeed both necessary and sufficient for associative memory function in both Hebbian Hopfield Networks and infomorphic Hopfield Networks. As an early conceptual study, Makkeh et al. [13] made several non-standard choices in the design of Hopfield networks, which we improve upon here: testing was performed using stochastic activations, underestimating the measured capacity, and the setup of the target weights $\boldsymbol{w}_T$ differed from the one employed here (see section above).

The effects of individual changes on the final performance of the model are discussed in the new section in Appendix H. Finally, in comparison to [13], we simplified a number of architectural choices, which we did not reproduce for this ablation study for their negligible impact on performance; these include: removing the additional decoder layer, using a simple additive activation function $A(t, r) = r + t$ instead of a complex non-linear activation function, removing terms

from the goal function that had been introduced for stability, and eliminating sequential dependencies in training by re-initializing the activations for each new pattern, to name a few. These simplifications show that the choices in [13] were not essential to achieve high memory capacity, and that redundancy as a goal is more robust than can be inferred from [13] alone.

> - Second, since these capacities are empirically calculated (without any theoretical evaluations), it is not clear if such a high memory capacity will be obtained across varying values for the number of neurons N — does increasing the number of neurons increase or decrease the capacity? Would the "finite size effects" reported in Appendix E imply that the capacity increases with N as the redundancy estimate gets better?

The observed capacity of the redundancy goal does not change as the network size is increased further. We extended the range of network sizes shown in Appendix D.3 to clearly show this. For very large numbers of neurons above $N \approxeq 800$, the scale of target weights needs to be increased to retain a very high capacity. When scaling this parameter appropriately, we observe small gains in capacity α_c^{red}. These gains are still in line with the expected linear scaling. Separately, the finite size effects only appear when the absolute number of patterns to be learned is too small to guarantee that both possible target inputs $T \in \{-w_T, w_T\}$ are realized for all neurons. The effect of only one target input being realized results in $H(T) = 0$, $I(Y : T) = 0$ and $\Pi_{\mathrm{red}} = 0$, representing a limitation of information theory. As a consequence, small networks at very small memory loads do not learn properly, however these effects disappear quickly with increased α .

> - Another confusing aspect of the results in Figure 2, especially Figures 2C,D, is that, while maximizing redundancy seems to increase memory capacity, it is unclear why the accuracy drops at $\alpha_c^{red} =$ 1.6 when the redundancy continues to be almost as high as possible (close to 1.0). While the authors briefly discuss this in lines 145-147, I think this warrants a more technical explanation. To me, this result seems to imply that high redundancy does not necessarily imply high accuracy. Since the 1.6 is not a theoretical capacity limit, it is just not clear up until which point would high redundancy and high accuracy coincide, and when do high redundancy start co-occurring with low accuracy. If redundancy is the underlying mechanism, the slight drop in redundancy should only translate to a slight drop in accuracy, not a sharp drop as seen in Figure 2C,D. Thus, this result seems counter-intuitive to me.

Even though our results suggest redundancy maximization as the underlying principle behind associative memory learning, we make no formal connections between specific values of redundancy and memory capacity. However, the sharp drop in recall accuracy can be understood through the dynamics of the Hopfield networks during retrieval: The PID analysis is performed at the first step, where all other neurons are still initialized in the correct pattern state. During subsequent retrieval steps, a single neuron error can propagate through the recurrent connections, causing further neurons to produce incorrect outputs. This effect explains why a small decrease in redundancy can lead to a sharp drop in final recall accuracy.

> - Finally, it would be good to show the computational complexity of the redundancy maximizing weight learning related to the Hebbian learning scheme, or other baselines. While I understand that the PID based schemes would be expensive and not scalable as it requires the estimation of joint probabilities, it would be good to get a sense of the kind of computation we would need to spend relative to existing schemes.

Since our approach uses an iterative learning procedure in contrast to the single-step algorithm for Hebbian learning, the computational complexity of our procedure is indeed significantly higher.

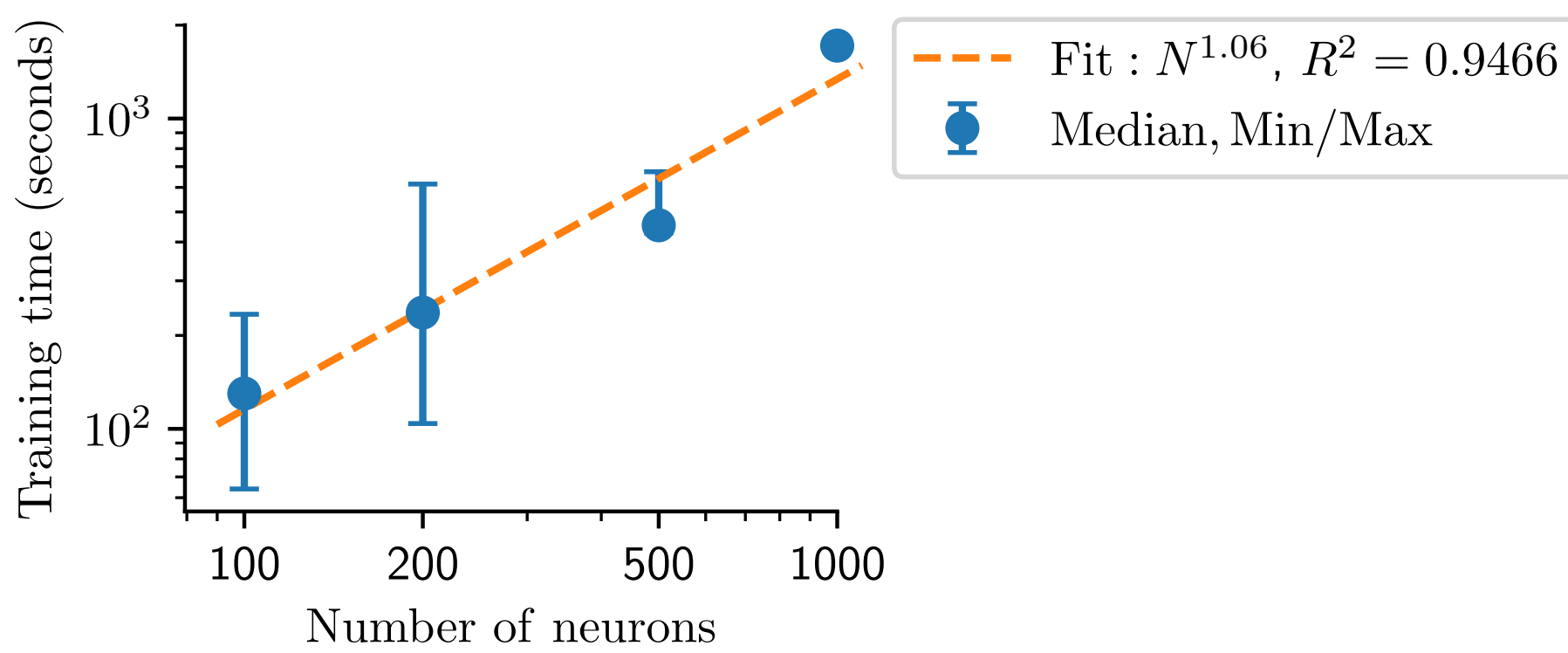

Figure 20: **Training time of infomorphic Hopfield Networks scales linear with the number of neurons.** The training times for infomorphic Hopfield networks with varying numbers of neurons are shown. The memory load is fixed at $\alpha = 0.2$. A least square fit returns a scaling exponent of 1.06 which is slightly above linear as expected.

However, as the recurrent input of each neuron is first integrated into a scalar before calculating the joint probabilities, the direct scaling with respect to the number of neurons is linear. While the lateral weight matrix itself grows quadratically with the number of neurons, this does not dominate the overall training time: in Figure 20, we show that the empirical scaling exponent is approximately 1.06, i.e. nearly linear. The training was done on a CPU-cluster node with 2 Sapphire Rapids 8468. Due to the vectorized structure of our implementation, the training is more efficient on a GPU machine. However, this near-linear scaling holds only as long as the hardware can fully parallelize the computation. Once the parallelization capacity is exhausted, the quadratic growth of the weight matrix becomes the bottleneck, resulting in expected quadratic scaling with the number of neurons.

Nevertheless, the aim of this work is not to suggest a practical replacement to Hebbian networks or modern associative memory networks, but to uncover a general principle that these schemes adhere to and through which improvements to the more practical implementations may be suggested.

Regarding the analysis comparing redundancy maximization to mutual information maximization, the analysis needs to be further clarified:
- First, it would be good to understand why we only consider the input-target mutual information I(Y:T) and co-information I(Y:R:T), and not something like the I(Y:R,T)?

The output-target mutual information $I(Y : T)$ and co-information $I(Y : R : T)$ were chosen because of their intuitive suitability to the associative memory problem. Nevertheless, we empirically tested $I(Y : R, T)$ and found it to produce a capacity of $\alpha_c \approx 0.85$. The other simple candidate goal function $G = I(Y : R)$ was found to not work at all. We have included both results into the main text.

- In the maximization of G = I(Y:T), it is not clear why the redundancy Π_{red} starts dropping beyond the capacity in Figure 3C, but it does not with $G = \Pi_{red}$ (as seen in Figure 2D). As there are weight configurations with high redundancy (as in Fig 2D), shouldn't maximizing $I(Y : T) = \Pi_{red} + \Pi_{unq,T}$ pick configurations where Π_{red} continues to be large beyond the α_c^{red} as these configurations would have higher I(Y:T). The only setup where this would not be true is if the unique information $\Pi_{unq,T}$ goes dramatically negative. However, this situation is very odd as one should not expect negative unique information.

As $I(Y:T) = \Pi_{\text{red}} + c\Pi_{\text{unq},T}$, maximizing $I(Y:T)$ does not necessarily find the solution that also maximizes Π_{red} on its own if a direction with a strong gradient to $\Pi_{\text{unq,T}}$ overcompensates for a relative loss of redundancy. Optimizing redundancy alone reliably selects the weight configuration which maximizes redundancy, thus necessarily matching or exceeding the redundancy found by other learning rules with additional terms. Why the local loss landscape changes for higher α in such a way as to tilt mutual information maximization to a solution with more unique information, however, remains an open question. While SxPID (based on the $I_{\cap}^{\text{sx}}$ redundancy measure) allows negative values for the unique information terms in principle [21], this does not appear to be relevant to the phenomenon in question.

- Thus, the justification for claiming that maximizing mutual information implicitly maximizes redundancy seems insufficient. Furthermore, it is important to understand what happens at the threshold α_c^{red} that $I(Y:I)$ starts diverging from Π_{red} (although, to me it seems like they should not as per my above comment about the optimization).

In our results, we show that both maximizing mutual information and maximizing redundancy directly lead to high redundancy below capacity. Since removing the unique information from the mutual information goal function does not majorly negatively impact performance, we suggest that it is the redundant contribution which primarily determines task performance.

- In fact, if redundancy maximization is truly sufficient, the capacity should not be affected at all by $\gamma_{unq,T}$ in Figure 3D as we are maximizing the redundancy in all cases. However, the results show that negative values of $\gamma_{unq,T}$ (implying also minimizing $\Pi_{unq,T}$) actually hurts capacity.

As mentioned before, optimizing a goal function containing a positive term for redundancy does not necessarily result in the same maximal redundancy as optimizing for redundancy alone. We see in our experiments that whenever a goal function manages to find a high-redundancy solution, the task performance is likewise high. Therefore, sufficiency of redundancy does not imply that optimizing for any goal function involving redundancy will yield maximum performance. Rather, our claim is the inverse: If a good solution is found, it must have—explicitly or implicitly—optimized for redundancy.

The analysis using hyperparameter optimization for the goal parameters is very interesting. However, the results highlight the lack of interpretability in the results (which is counter to the claims of interpretability in the PID framework). For the optimal hyperparameters in Figure 4C,D, it is odd that $\gamma_{unq,T}$ is both positive and negative, implying that we can either maximize $\Pi_{unq,T}$ or minimize it, which makes the decomposition uninterpretable. This is also counter to the results shown in Figure 3D, which implied that negative $\gamma_{unq,T}$ leads to worse capacity, and increasing it does not affect capacity significantly. Thus, it is unclear how we can square the resuts in Figure 3D with the hyperparameter optimization results in Figure 4C,D.

Our results show that high task performance can indeed be achieved both with positive or negative values for $\gamma_{\text{unq},T}$. This implies that adding or removing unique target information as a goal does not majorly affect the results, matching our hypothesis that the optimization for redundancy is the decisive factor in determining task performance. Upon close inspection, the results shown in Figure 5 (previously Fig. 4) are in agreement with those in Figure 4D (previously Fig. 3D): There, the goal parameter for redundancy is fixed at 1 and the parameters for synergy and recurrent unique are fixed at 0. In Figure 5A, these values can be found as the center points in the rightmost column, which do show decreasing performance for negative contributions of $\gamma_{\text{unq},T}$.

Minor typographical error

- In line 201, I think the authors wish to refer to Figure 5C, not Figure 5B.

We thank the reviewer for pointing this out, we fixed it.

Conclusion

Overall, in this manuscript, the authors put forward an intuitive, though somewhat expected and known as per [13], objective for learning weights in the classical associative memory network. However, the objective itself requires the hard estimation of joint probabilities, and the current manuscript does not discuss how the estimation scheme and quality affects the results in this manuscript. Furthermore, the results are empirical in nature, and are limited to a small number of configurations of associative memory networks, making it unclear whether these results are robust and significant in general.

We thank you for your insightful and detailed feedback. With the additional experiments and clarifications that your comments have inspired, we believe that the strength of our arguments has been significantly improved.

Reviewer 2

In this paper, Blumel et al., use the partial information decomposition to interrogate the dynamics of associative memory in Hopfield networks, finding that Hebbian learning seems to be driven by redundancy, and apparently compromised by unique and synergistic interactions. They then go on to show that an "infomorphic neuron", trained to maximize redundant information (or some linear combination of partial information atoms) can achieve very high performances on an associative learning task - comparable with current state of the art.

In general, I am a big fan of this paper. I think this is an excellent application of PID to a concrete and well-defined problem. I am also very enthusiastic about the use of information-theoretic objective functions. So much of information theory in complex systems stops at the level of descriptive statistics (e.g. neuroscience studies that ask "is there more synergy in an awake brain than an unconscious one"), and it is exciting to see a new spin on these methods.

In general the methodology seems strong and the results robust. I have some small technical quibbles, but they can be addressed with minor revisions.

One thing that gives me pause is that the authors also try to maximize the co-information, and find that no patterns are stored. I cannot find any place in the paper where the definition of co-information is given, so I will assume that here the definition is the same as the original Bell paper [1], which is positive when redundant interactions dominate, and negative when synergistic interactions dominate. Later, Williams and Beer [2] showed that, for two sources X_1, X_2 and one target Y, the co-information is equivalent to Red(12 ; Y) - Syn(1,2 ; Y). Based on this identity, I find it a little concerning that optimizing the co-information apparently does not perform at all, even when the i_{sx} redundancy function does. If it were the case that redundancy generally improves performance, then naively, I would expect optimizing the co-information to perform equivalently well, especially since the goal function $G^{(i)}$ has a very co-information-like form (positively weighting redundancy, negatively weighting synergy).

As it stands, I don't know what to make of this apparent inconsistency. Are the authors sure that the co-information was implemented correctly? The literature on co-information is messy, sometimes co-information is used to refer to the inverse-signed measure, which is Syn(1,2) ; Y) - Red(12 ; Y).

To avoid ambiguity, we have added the formal definition of co-information to our manuscript and confirmed that the definition of co-information used by us indeed corresponds to redundancy minus synergy (see also Fig. 4A depicting the classical co-information goal on the right). We examined the network's learning behavior, varying only the goal parameters for redundancy and synergy (see Figure 21A). The results indicate two distinct regions with non-zero performance, separated by minimal performance exactly at the line of (multiples of) co-information as goal function ($\gamma_{\mathrm{red}} = -\gamma_{\mathrm{syn}}$). Thus, while we can confirm that co-information itself is not a valid goal function, adding even small amounts of redundancy leads to a goal function with intermediate capacity $\alpha_c \approx 0.8$. If a purely classical goal function is desired, adding $I(Y : T)$ or $I(Y : R, T)$ to the goal function also has the same effects. Nevertheless, why only the exact goal of (multiples of) co-information produces lower performance than small perturbations from it remains a striking result warranting a more comprehensive analysis in future work.

> Another small thing is that I'd like to see a cartoon that describes Hopfield networks a little more intuitively. Fig. 1A kind of starts, but it's still a little abstract. Is there a visualization that describes the entire process up updating (including intuitive representations of equations like Eqs. 1 and 2? I think this would go a long way towards making the paper more accessible.

We thank the reviewer for this suggestion. We added a new introductory figure (new Figure 1) that illustrates the retrieval process of a Hopfield network, including stored patterns, the recurrent weight structure, the iterative update dynamics, and the energy landscape perspective. We hope this makes the paper more accessible to readers less familiar with Hopfield networks.

> All in all, I commend the authors for an impressive piece of work.

[1] Bell, A. J. (2003, April). The co-information lattice. 4th International Symposium on Independent Component Analysis and Blind Signal Separation, Nara, Japan. [2] Williams, P. L., & Beer, R. D. (2010). Nonnegative Decomposition of Multivariate Information. arXiv:1004.2515

Reviewer 3

> The authors provide an analysis of Hopfield networks based on partial information decomposition (PID). The authors find empirically that high redundancy is correlated with high recall accuracy in Hopfield networks, but is not strongly correlated with other components used in the PID profile. This is used as motivation to optimize for redundancy. Building on previous work on infomorphic networks, they design a type of Hopfield network that optimizes the informorphic objective, which is composed of a weighted sum of PID components. They run multiple experiments that test various weightings of the infomorphic objective, including hyper-parameter sweeps, and observe that redundancy is the most important component for associative memory recall accuracy. They also show that their objective leads to better accuracy than other SOTA objectives at high memory loads.
> The authors perform an insightful and novel analysis of Hopfield networks based on PID, and they use this analysis to improve performance of auto-associative networks over SOTA methods. The paper is well written, the diagrams provide useful explanations of concepts and experiments, and the experiments are logical.
> Below I provide suggestions for making the paper a bit clearer for those new to PID, and how to strengthen the empirical results and explanation of the results.
> For equations 3 and 4, I believe it would be helpful to have a table, possibly in the appendix, that explicitly defines the Π terms (Π_{syn},Π_{red}, etc.). These are nicely visualized in Figure 1C, but I can't seem to find an explicit mathematical definition in the paper. They can be inferred from equations 3,4, and the diagrams, but an

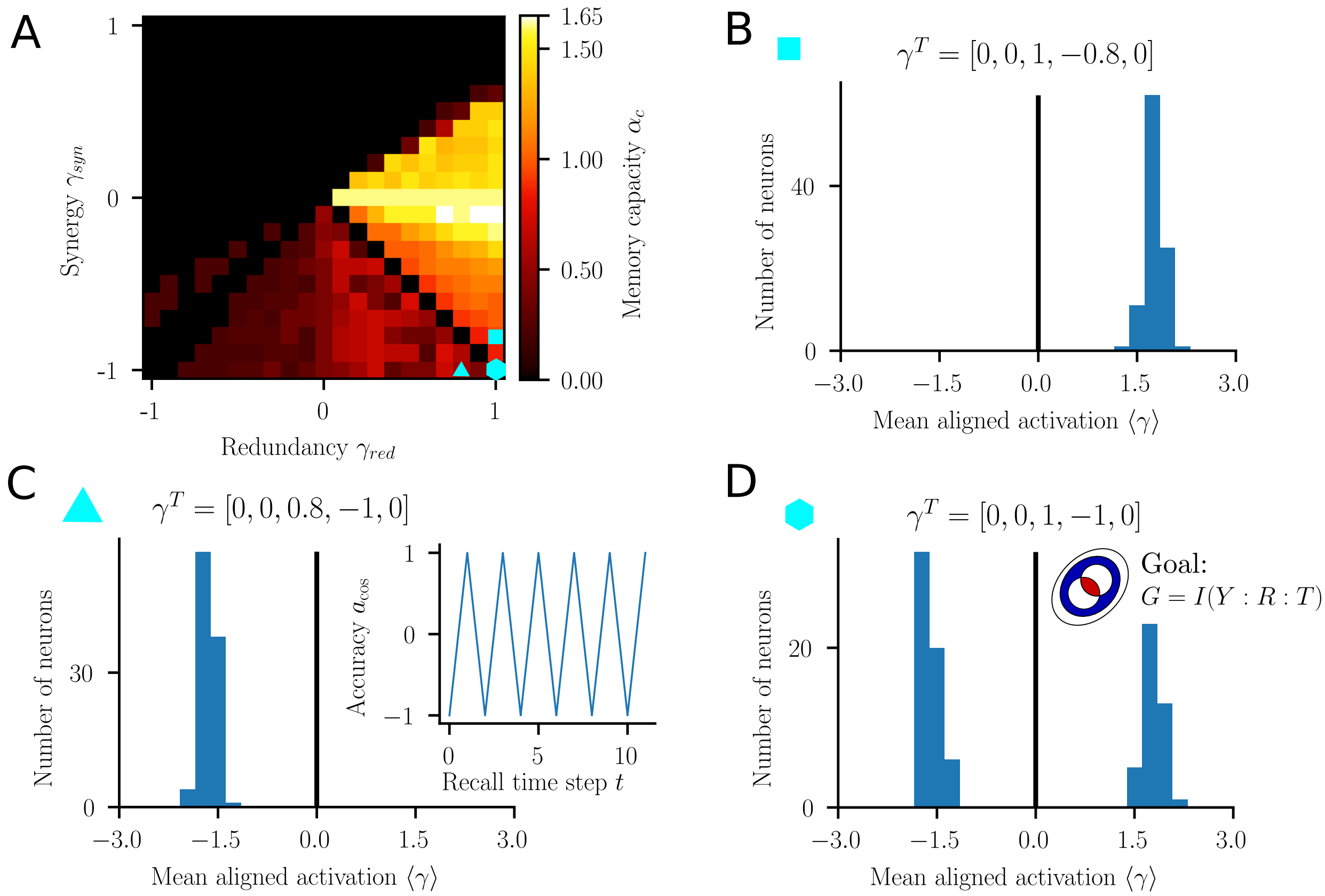

Figure 21: **Co-Information seems to fail as a goal because it is located directly between two regions with qualitatively different solutions. A:** The memory capacity is shown in the goal space as a function of goal strength for redundancy γ_{red} and γ_{syn}. The landscape shows two regions of successful solutions, one centered around redundancy $G = \Pi_{\text{red}}$ and one around negative synergy $G = -\Pi_{\text{syn}}$. Co-information lies on the diagonal exactly separating the two. **B:** The panel shows the frequency of mean alignment $\langle\gamma\rangle = \langle\frac{RT}{|W_i|}\rangle$ between target T and recurrent activation R at each neuron after training primarily on redundancy. The network was trained at a memory load of $\alpha = 0.2$ and all neurons have positive mean alignments. Panels **B-D** have their exact goal marked in cyan in Panel A. **C:** Networks trained primarily on minimizing synergy lead to oscillations between the pattern and its mirror image. The inset shows accuracy as a function of recall time stept t for the goal function $G = 0.8\Pi_{\text{red}} - \Pi_{\text{syn}}$. Below the (reduced) memory capacity α_c, accuracy oscillates between 1 and -1 during recall. In accordance with this, the mean alignment $\langle\gamma\rangle$ is negative for all neurons. **D:** When training on co-information, the mean alignments $\gamma = \langle\frac{RT}{|W_i|}\rangle$ split into two. Each neuron shows either a strong positive or negative mean level of activation, thus representing a mix between the solutions seen in the two regions of goal space.

explicit definition would be nice. If these definitions do exist in the paper, could the authors make sure there is a clear reference to it? Otherwise, could the authors spell these definitions out somewhere?

We thank the reviewer for pointing this out. The information atoms are calculated via Moebius inversion of Eq.3 after defining a measure for redundant information. We have added the explicit calculation of information atoms to Appendix subsection A.1 and reference it in section subsection 2.2 of the main text.

> From what I could tell, the authors ran all of their tests from section 3.2 onwards with networks of size 100 neurons, and section 3.1 experiments with networks size 500 neurons. Is this correct? If so, could the authors also run tests on other sized networks? I believe it is important the authors establish that their empirical results generalize to any reasonably sized network. For example, maybe they could test at [50, 100, 200, 400, 800] neurons to see if there are any clear trends as they double the size of the network. Maybe doing this for the tests in figure 2 and figure 5 would be sufficient. Also, showing the findings from section 3.1 generalize to at least one more network size would be useful too. These extra results can then be placed in the appendix and noted in the main text.

This is a very valuable comment. Indeed, we implicitly claim that memory capacity scales linearly with the number of neurons in the network, but neglected to provided evidence for this hypothesis. As requested, we have run experiments for multiple network sizes and added the results to Appendix E (Figure 8), with references from the main text. Both memory capacity α_c and stability $f_{\max}$ are found empirically to scale linearly with network size, up to some minor differences in shape.

> Would it be feasible for the authors to test a version of their memory network on continuous valued vectors, e.g., vectors with values between 0 and 1 or between -1 and 1? I suspect this may best be left to future work, but I was curious what the authors' thinking is about this.

This is a very intriguing question, especially against the background of recent successes in continuous-valued Hopfield networks. Unfortunately, our framework would need major adaptations to accommodate for continuous targets. While infomorphic neurons do in fact easily deal with continuous inputs due to the binning procedure (see for example Schneider et al. [14]), their outputs are discrete and currently also binary, because the discrete PID measure requires us to calculate probabilities for each output. This discreteness in the output would inevitably lead to poor reconstruction performance for continuous-valued memory patterns because no matter which pattern was put into the network, all attractors would be $\in \{-1, 1\}^N$. Overcoming the limitation of discrete outputs would require using continuous PID estimators. While continuous PID estimators exist (e.g., [35]), they are typically not differentiable and their introduction would require a major re-work of the current framework for infomorphic neurons, a comprehensive account of which we will have to leave to future work.

> The authors provide empirical motivation for optimizing for redundancy for auto-associative memory. However, I think the paper lacks a clear mathematical intuition for why this should be the case. For example, I do not see an attempt in the discussion section to provide mathematical intuition for why, for example, redundancy should be more important than mutual information or other similar measures when training auto-associative memory units. Do the authors believe there is good mathematical intuition for why we see the empirical results we do? Is there reason to believe this empirical result is not somehow unique to the particular experiments ran in the paper (e.g., with binary vectors, at 100 neurons, etc.) and generalizes beyond it? If so, what is this explanation?

We thank the reviewer for raising this important question, which prompted us to substantially strengthen the theoretical foundations of our work. We have added a comprehensive analytical treatment in Appendix F and summarize the core arguments below.

Why redundancy is *necessary* for associative memory. In Appendix F.1, we provide a formal proof that high redundancy is a necessary condition for successful associative memory function, independent of network size or how the weights are obtained. For successful recall under a Heaviside activation, each neuron's recurrent input R must satisfy $\mathrm{sgn}(R) = \mathrm{sgn}(T)$ for almost all samples. Geometrically, the conditional distributions $p(R \mid T = +w_T)$ and

$p(R \mid T = -w_T)$ must be supported on opposite sides of $R = 0$. Starting from the $I_\cap^{\mathrm{sx}}$ definition, we show that whenever these supports are disjoint in this way, the union probabilities collapse and redundancy attains its maximum value $H(Y) = H(T) = 1$ bit. Conversely, *any* overlap across the decision boundary strictly decreases redundancy. Since disjoint supports are precisely the condition for perfect recall, redundancy is provably maximal if and only if associative memory is successful.

Why redundancy as the underlying principle and not mutual information or co-information. The reviewer asks specifically why redundancy should be more important than classical measures. The PID consistency equations give a clean answer: classical information-theoretic quantities can be maximized in two ways—one that increases its redundant component and supports associative memory, and one that increases other components that do not. **Target mutual information** $I(Y : T) = \Pi_{\mathrm{red}} + \Pi_{\mathrm{unq},T}$ can be raised either by increasing Π_{red} (the recurrent input genuinely encoding the target) or by increasing $\Pi_{\mathrm{unq},T}$ (the neuron simply copying the teaching signal). Below capacity, gradient ascent finds the redundancy maximization route, which is why $I(Y : T)$ matches redundancy in performance (Figure 4**C**). Above capacity, however, the shortfall in achievable Π_{red} is compensated by persistent $\Pi_{\mathrm{unq},T}$, keeping $I(Y : T)$ high even as recall degrades. Under $G = \Pi_{\mathrm{red}}$, by contrast, $\Pi_{\mathrm{unq},T}$ does not increase above capacity (Figure 3**D**). **Co-information** $\Pi_{\mathrm{red}} - \Pi_{\mathrm{syn}}$ fails as a goal because it leads to a mix of two qualitatively different solutions (see Figure 21). **Recurrent mutual information** $I(Y : R)$ does not constrain alignment with T and similarly fails.

Generality of the results. We support the generality of our core results with several additions to the appendix:

- Analytical derivation for Hebbian networks (Appendix F.3): A closed-form expression for $I_\cap^{\mathrm{sx}}(Y : R; T)$ as a function of α and N reproduces the empirical profile of Figure 2D (Figure 13**C**), confirming the signature is not an artifact of finite sampling or binning.
- Robustness across PID measures and binning (Appendix F.4, Figure 14–Figure 15): The qualitative profile is preserved under $I_\cap^{\mathrm{BROJA}}$, $I_\cap^{\min}$, I^{PM}, and I^{MMI}, and across a range of bin counts.
- Scaling with network size (Appendix E, Figure 8–Figure 9): Capacity and stability scale linearly with N from 50 to 800 neurons.

We have updated the Discussion to point readers to Appendix F and added cross-references in Sections 3.1 and 3.3 of the revised manuscript.

Response to Reviewers

We would like to thank the reviewers for their careful and constructive feedback. In response, we made substantial additions to the manuscript, including: (i) a new introductory figure illustrating Hopfield network retrieval process, (ii) a formal analysis of why Hebbian learning produces high redundancy, with both an analytical derivation and an intuitive visualization (new Appendix F), (iii) validation of our PID analysis using alternative measures and estimation parameters (new Appendix F.4), (iv) ablation experiments quantifying each methodological improvement over prior work (new Appendix H), and (v) scaling experiments across networks size (new Appendix E). Below we provide a detailed point-by-point response to each of the reviewers' comments. Modifications in the manuscript are highlighted in red.

Reviewer 1

Contributions

Based on the partial information decomposition or PID framework, this manuscript studies the role of the local redundant information (between the node input and the target output) in learning the weights of an associative memory network. First, the manuscript provides empirical evidence that this redundant information is high in a classical associative memory network (below memory capacity) where the weights are learned using the standard Hebbian rule. Using this as a motivation, the manuscript proposes the learning of weights by explicitly maximizing the redundant information, and demonstrates that this increases the memory capacity. Furthermore, more empirical results are provided to highlight how maximization of mutual information results in similar memory capacity, and performing a hyperparameter optimization of the different PID term weights (in a weighted sum objective) can further improve the memory capacity. Finally, comparison to other weight learning schemes highlight the improved memory capacity of the redundancy maximizing weight learning.

We thank the reviewer for their thorough and constructive review. In the following, we address each of the raised points individually.

Novel & significance

First, it is not clear how novel or significant is the main thesis of this manuscript that the goal of any (supervised) learning is to the maximize the redundant information between the input and the target output. This was already presented in [13] for both supervised learning, and associative memory learning. If redundancy maximization between input and output is the main contribution, the novelty is somewhat limited.

Makkeh et al. [1] provided proof of concept that redundancy maximization can be sufficient for associative memory learning with limited performance. To achieve this, they made a series of unconventional model choices, such as adding a separate readout layer, ignoring part of the gradients due to a non-differentiable binning procedure, choosing a tailored activation function and adding other information atoms besides redundancy in their goal function for stability reasons.

In this work, we significantly expand on this idea in three major ways. First, we show that redundancy is also increased by Hebbian learning for associate memory networks, providing evidence that the seemingly ad-hoc goal function chosen by Makkeh et al. [1] might in fact point to a more fundamental underlying principle. Second, by incorporating important improvements and simplifications to the infomorphic Hopfield framework which address the above-mentioned issues, we show that the associative memory performance of redundancy maximization matches or even exceeds state-of-the-art learning rules. To demonstrate the impact of each of these improvements, we have added an ablation study to Appendix

H (Figure 17). Third, we demonstrate via hyperparameter optimization that in addition to maximizing redundancy, suppressing other information terms can further improve the memory capacity.

Overall, through the analysis of redundancy in Hebbian networks, state-of-the-art performance and hyperparameter optimization, this work provides a comprehensive understanding of associative memory learning through the principle of redundancy maximization.

> One potential novel aspect is what is listed in lines 48-50 as "an information-theoretic analysis of a classical Hebbian Hopfield network which reveals that redundancy between recurrent input and target (teaching signal) dominates the neurons' output when operating below capacity". However, the validation of this claim is somewhat limited:
> - Figure 1D is solely an empirical observation and relies on how the redundancy and other information related terms are computed. Relying on single method of empirical evaluation of these terms, such as I^{sx}_{cap}, does not necessarily imply that the dependencies we see are not an effect of the (inaccuracies in the) empirical estimates. In fact, as shown in Appendix E, for small values of N (implying less reliable estimates of information-theoretic quantities), the high redundancy shown in Figure 1D at below capacity does not hold. This makes it unclear whether the high redundancy of the Hebbian weights (below capacity) is due to true underlying connection or an artifact of the measurement quality.

We agree that the estimation of information quantities can be challenging, and that our results could benefit from additional validations of our methodology. To this end, we have added the following validations: First, we analyzed the Hebbian Hopfield networks using other PID measures, to ensure that the observed trends are not due to the idiosyncrasies of the chosen $I_\cap^{\mathrm{sx}}$ PID measure (see Figure 14). Second, we reran our analysis with varied estimation parameters, such as different numbers of bins in the estimation of the probability distributions (see Figure 15). We find that these variations produce qualitatively identical results, and have added an appendix section (Appendix F.4) where we discuss the results for those comparisons in detail. Additionally, we also added an analytical derivation of redundancy in Hebbian Hopfield networks (Appendix F.3). While the derivation uses the definition of $I_\cap^{\mathrm{sx}}$, it is independent of any binning assumptions and shows the same behavior as our empirical results.

Note, however, that the finite size effects discussed in Appendix E are not related to the estimation method, but point to a general limitation of information theory. For small number of patterns (necessary for studying low α in small networks), by chance specific elements of the target pattern can be constant over all patterns, resulting in vanishing entropy. With the entropy providing an upper bound to redundancy, there can accordingly also be no redundancy in these situations, rendering a PID analysis futile. To further clarify this effect we expanded Appendix E.

> - Given that the information-theoretic quantities such as redundancy are quite formally defined, this would be a more significant contribution if there was a theoretical (or at least more technical) intuition of why Hebbian weights have high redundancy. This is an expected result as the Hebbian weights are computed using the targets, but a formal connection would be of interest.

We thank the reviewer for this suggestion. As already mentioned, we added a new appendix section (Appendix F.3) that provides an analytical derivation of redundancy in Hebbian Hopfield networks based on the definition of $I_\cap^{\mathrm{sx}}$. We also added more technical intuition, based on the separation of the conditional input distributions $p(R \mid T = +1)$ and $p(R \mid T = -1)$ and the properties of the pointwise $I_\cap^{\mathrm{sx}}$ (Appendix F.1). To support this part, we also included new figures (Figures 9-11). We have placed this section in the appendix rather than the main text, as the formal treatment involves details of the $I_\cap^{\mathrm{sx}}$ measure that may be too technical for the main text.

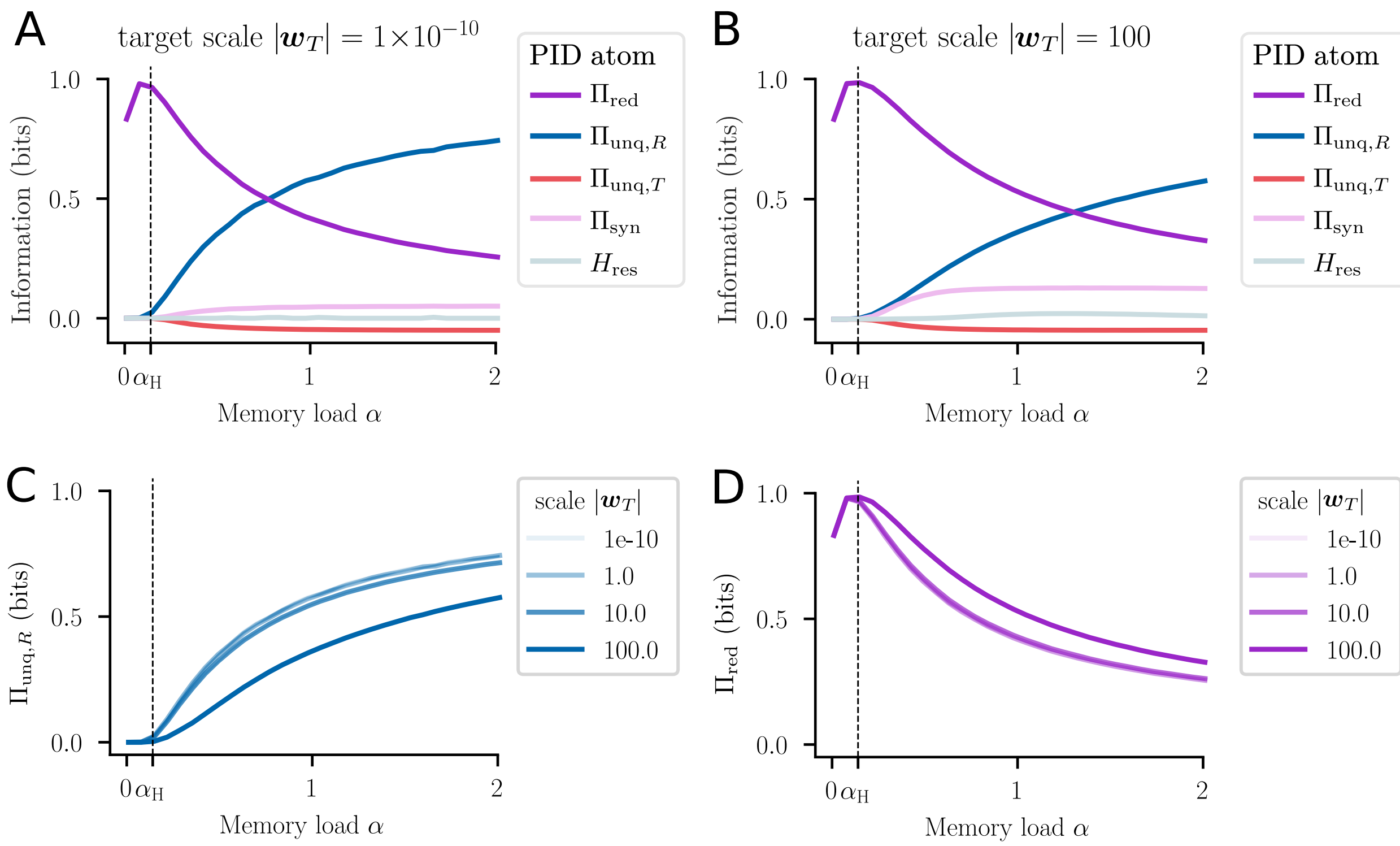

Figure 1: **Increasing the target weights w_T does not qualitatively change the transition in redundancy for Hebbian learning. A:** The PID profile for Hebbian learning as depicted in Fig. 2D. The target weights are set to $\boldsymbol{w}_T = 1 \cdot 10^{-10}$. Above the memory capacity, falling redundancy is replaced with recurrent unique $\Pi_{\text{unq},R}$. **B:** The PID profile is reproduced with target weights $w_T = 100$ now dominating the activity of the network. Above the memory capacity, falling redundancy is replaced with both recurrent unique $\Pi_{\text{unq},R}$ and synergistic information Π_{syn}. **C-D:** The changes in unique recurrent information $\Pi_{\text{unq},R}$ (Panel **C**) and Π_{red} are shown as a function of target weights $|\boldsymbol{w}_T|$. For larger target weights there is a relative drop in recurrent unique information and an increase in redundancy at high memory loads. The memory load α at which the transition in redundancy occurs is not affected by the scale.

- Finally, it is unclear how robust the estimated redundancy of the Hebbian weights are as it is not clear how the necessary w_T, the weights for the target output, are obtained as they are not part of the Hebbian learning. It seems as per Appendix A that w_T is never learned in any of the experiments, and set to 2.3. If this is the value used to compute the redundancy between the target and input in a network with Hebbian weights, it would be important to understand how this value affects the presented results in Figure 1D (and elsewhere).

We thank the reviewer for this valuable comment. Indeed, the target weights $\boldsymbol{w}_T$ are set to 1×10^{-10} for our analysis of Hebbian networks to reflect the lack of a direct target input in conventional Hebbian Hopfield networks. We have included this setting in Table 1 now. Additional experiments show that the PID profile obtained in Figure 8 is robust with respect to changes in $\boldsymbol{w}_T$ (see Figure 13), unless the target input becomes too large (and thus driving). Here, we varied the scale of target weights from 1×10^{-10} to 100, while keeping the recurrent weights $\boldsymbol{w}_R$ fixed at the value obtained from outer-product learning. As the target weights become driving, the transition in PID atoms still occurs around the Hebbian capacity α_H. At the same time, the increase in unique recurrent information $\Pi_{\text{unq,R}}$ becomes smaller, while there is less loss of redundancy Π_{red} and some gain in synergy Π_{syn}.

When considering infomorphic Hopfield networks actively trained on redundancy, the scale of target weights $\boldsymbol{w}_t$ does have an impact on the memory capacity (see also Figure 17). As can be seen in Figure 17, we chose the value of 2.3 from a range of values that maximize capacity. However, even if this scale is not set optimally or if the target weights are learned during training, high capacities far exceeding α_H are still attainable.

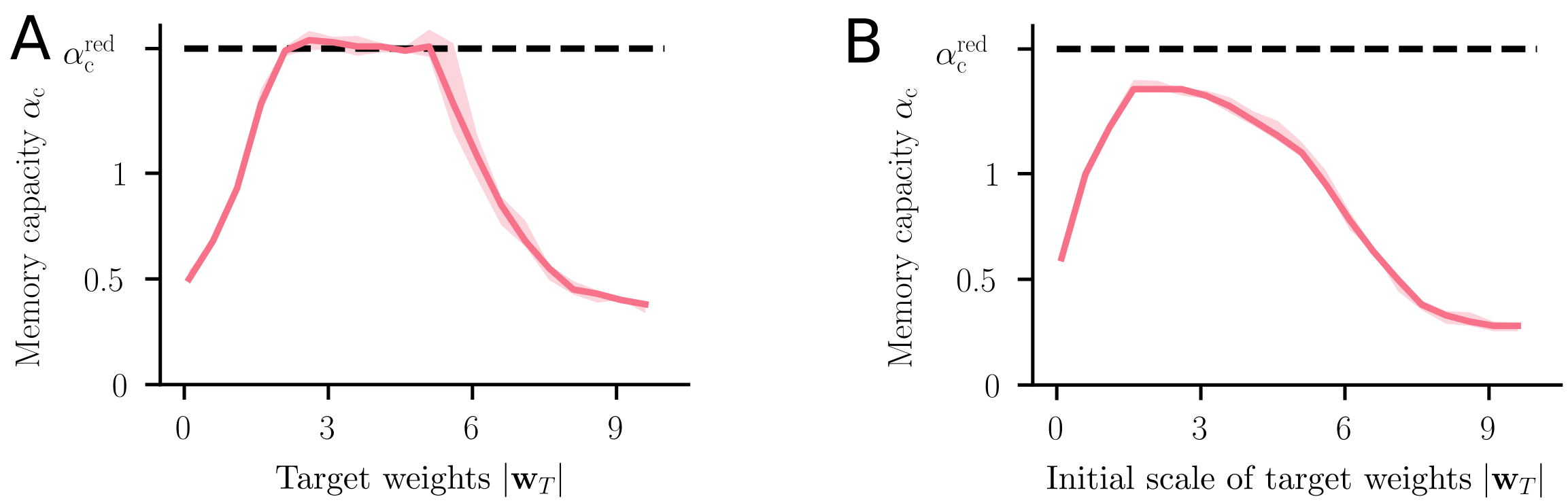

Figure 2: **The memory capacity of infomorphic Hopfield networks exceeds the Hebbian baseline even for far-from-optimal target weight settings. A:** Memory capacity α_c is shown as a function of scale of target weights $|\boldsymbol{w}_T|$. As for most experiments, here the target weights are frozen (not learnable) during training and the networks are trained with redundancy as a goal. There is a wide range of scales $|\boldsymbol{w}_T| \in (2.2, 5)$ for which the measured capacity plateaus. **B:** Same as panel A, but the target weights $\boldsymbol{w}_T$ are also learned during training.

Focusing on the redundancy maximizing weight learning scheme, the novelty is already limited given that Makkeh et al. [13] had already considered this. Authors say that they are "building on and improving the proof of concept provided by Makkeh et al. [13]", and explain in lines 226-231 that they are using a different activation scheme and binning procedure (to compute the information-theoretic quantities). However, there are some concerns here:

- First, it is not clear why these modifications should lead to such a significant increase in the memory capacity from the 0.35 reported in [13] to 1.6 in this manuscript. As noted by the authors, this is a very significant fourfold increase, and warrants a discussion (with theoretical intution and ablations) of why such an increase in capacity was obtained. Without this intuition, it is not clear what is it that is leading to the increase in capacity as both schemes are already maximizing redundancy.

We agree with the reviewer that the significant improvements over the results by Makkeh et al. [1] should be better highlighted with their individual impact on task performance clearly assessed. To this end, we have conducted ablation experiments, in which we successively remove the different optimizations to demonstrate their respective impact.

While Makkeh et al. [1] performed an initial investigation revealing that redundancy maximization can serve as a local goal to implement associative memories, the present work aims to test the hypothesis that redundancy is indeed both necessary and sufficient for associative memory function in both Hebbian Hopfield Networks and infomorphic Hopfield Networks. As an early conceptual study, Makkeh et al. [1] made several non-standard choices in the design of Hopfield networks, which we improve upon here: testing was performed using stochastic activations, underestimating the measured capacity, and the setup of the target weights $\boldsymbol{w}_T$ differed from the one employed here (see section above).

The effects of individual changes on the final performance of the model are discussed in the new section in Appendix H. Finally, in comparison to [1], we simplified a number of architectural choices, which we did not reproduce for this ablation study for their negligible impact on performance; these include: removing the additional decoder layer, using a simple additive activation function $A(t, r) = r + t$ instead of a complex non-linear activation function, removing terms

from the goal function that had been introduced for stability, and eliminating sequential dependencies in training by re-initializing the activations for each new pattern, to name a few. These simplifications show that the choices in [1] were not essential to achieve high memory capacity, and that redundancy as a goal is more robust than can be inferred from [1] alone.

> - Second, since these capacities are empirically calculated (without any theoretical evaluations), it is not clear if such a high memory capacity will be obtained across varying values for the number of neurons N — does increasing the number of neurons increase or decrease the capacity? Would the "finite size effects" reported in Appendix E imply that the capacity increases with N as the redundancy estimate gets better?

The observed capacity of the redundancy goal does not change as the network size is increased further. We extended the range of network sizes shown in Appendix D.3 to clearly show this. For very large numbers of neurons above $N \approxeq 800$, the scale of target weights needs to be increased to retain a very high capacity. When scaling this parameter appropriately, we observe small gains in capacity α_c^{red}. These gains are still in line with the expected linear scaling. Separately, the finite size effects only appear when the absolute number of patterns to be learned is too small to guarantee that both possible target inputs $T \in \{-w_T, w_T\}$ are realized for all neurons. The effect of only one target input being realized results in $H(T) = 0$, $I(Y : T) = 0$ and $\Pi_{\mathrm{red}} = 0$, representing a limitation of information theory. As a consequence, small networks at very small memory loads do not learn properly, however these effects disappear quickly with increased α .

> - Another confusing aspect of the results in Figure 2, especially Figures 2C,D, is that, while maximizing redundancy seems to increase memory capacity, it is unclear why the accuracy drops at $\alpha_c^{red} =$ 1.6 when the redundancy continues to be almost as high as possible (close to 1.0). While the authors briefly discuss this in lines 145-147, I think this warrants a more technical explanation. To me, this result seems to imply that high redundancy does not necessarily imply high accuracy. Since the 1.6 is not a theoretical capacity limit, it is just not clear up until which point would high redundancy and high accuracy coincide, and when do high redundancy start co-occurring with low accuracy. If redundancy is the underlying mechanism, the slight drop in redundancy should only translate to a slight drop in accuracy, not a sharp drop as seen in Figure 2C,D. Thus, this result seems counter-intuitive to me.

Even though our results suggest redundancy maximization as the underlying principle behind associative memory learning, we make no formal connections between specific values of redundancy and memory capacity. However, the sharp drop in recall accuracy can be understood through the dynamics of the Hopfield networks during retrieval: The PID analysis is performed at the first step, where all other neurons are still initialized in the correct pattern state. During subsequent retrieval steps, a single neuron error can propagate through the recurrent connections, causing further neurons to produce incorrect outputs. This effect explains why a small decrease in redundancy can lead to a sharp drop in final recall accuracy.

> - Finally, it would be good to show the computational complexity of the redundancy maximizing weight learning related to the Hebbian learning scheme, or other baselines. While I understand that the PID based schemes would be expensive and not scalable as it requires the estimation of joint probabilities, it would be good to get a sense of the kind of computation we would need to spend relative to existing schemes.

Since our approach uses an iterative learning procedure in contrast to the single-step algorithm for Hebbian learning, the computational complexity of our procedure is indeed significantly higher.

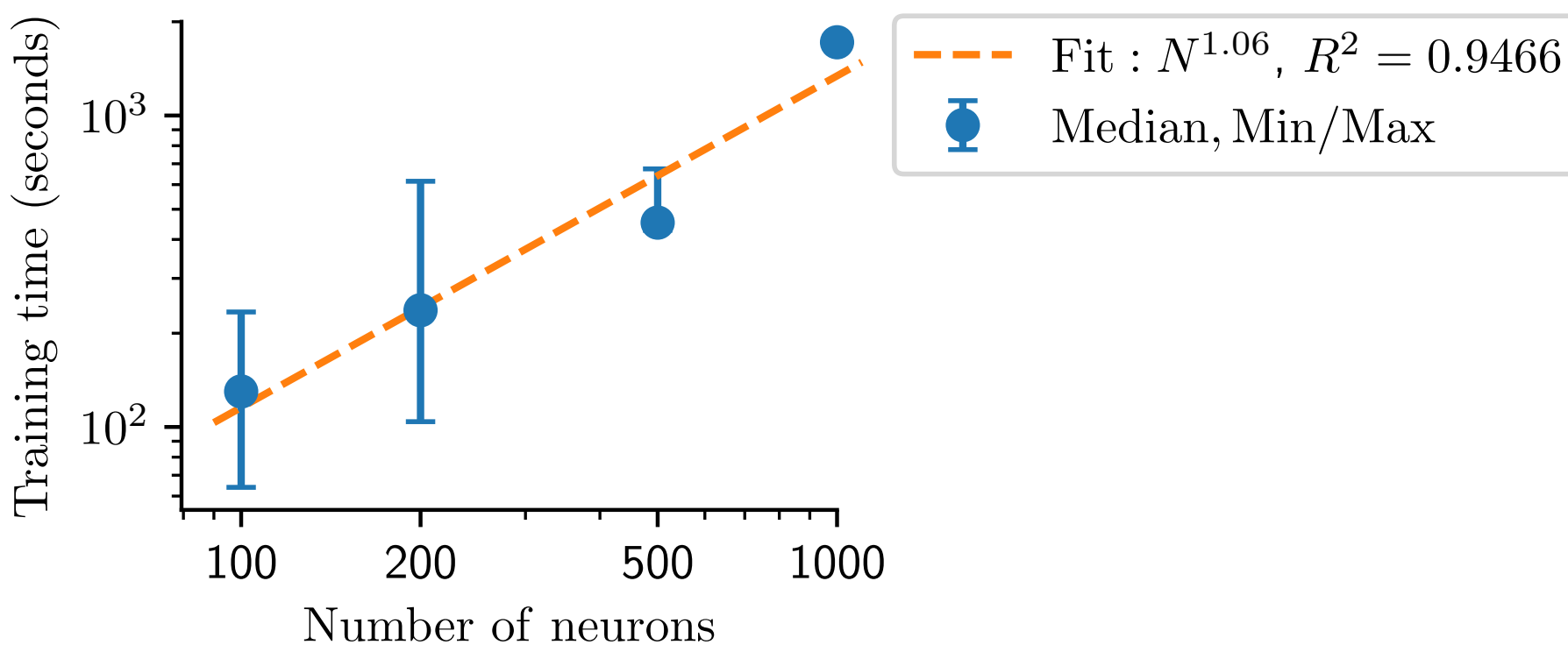

Figure 3: **Training time of infomorphic Hopfield Networks scales linear with the number of neurons.** The training times for infomorphic Hopfield networks with varying numbers of neurons are shown. The memory load is fixed at $\alpha = 0.2$. A least square fit returns a scaling exponent of 1.06 which is slightly above linear as expected.

However, as the recurrent input of each neuron is first integrated into a scalar before calculating the joint probabilities, the direct scaling with respect to the number of neurons is linear. While the lateral weight matrix itself grows quadratically with the number of neurons, this does not dominate the overall training time: in Figure 3, we show that the empirical scaling exponent is approximately 1.06, i.e. nearly linear. The training was done on a CPU-cluster node with 2 Sapphire Rapids 8468. Due to the vectorized structure of our implementation, the training is more efficient on a GPU machine. However, this near-linear scaling holds only as long as the hardware can fully parallelize the computation. Once the parallelization capacity is exhausted, the quadratic growth of the weight matrix becomes the bottleneck, resulting in expected quadratic scaling with the number of neurons.

Nevertheless, the aim of this work is not to suggest a practical replacement to Hebbian networks or modern associative memory networks, but to uncover a general principle that these schemes adhere to and through which improvements to the more practical implementations may be suggested.

Regarding the analysis comparing redundancy maximization to mutual information maximization, the analysis needs to be further clarified:
- First, it would be good to understand why we only consider the input-target mutual information I(Y:T) and co-information I(Y:R:T), and not something like the I(Y:R,T)?

The output-target mutual information $I(Y : T)$ and co-information $I(Y : R : T)$ were chosen because of their intuitive suitability to the associative memory problem. Nevertheless, we empirically tested $I(Y : R, T)$ and found it to produce a capacity of $\alpha_c \approx 0.85$. The other simple candidate goal function $G = I(Y : R)$ was found to not work at all. We have included both results into the main text.

- In the maximization of G = I(Y:T), it is not clear why the redundancy Π_{red} starts dropping beyond the capacity in Figure 3C, but it does not with $G = \Pi_{red}$ (as seen in Figure 2D). As there are weight configurations with high redundancy (as in Fig 2D), shouldn't maximizing $I(Y : T) = \Pi_{red} + \Pi_{unq,T}$ pick configurations where Π_{red} continues to be large beyond the α_c^{red} as these configurations would have higher I(Y:T). The only setup where this would not be true is if the unique information $\Pi_{unq,T}$ goes dramatically negative. However, this situation is very odd as one should not expect negative unique information.

As $I(Y : T) = \Pi_{\text{red}} + c\Pi_{\text{unq},T}$, maximizing $I(Y : T)$ does not necessarily find the solution that also maximizes Π_{red} on its own if a direction with a strong gradient to $\Pi_{\text{unq},\text{T}}$ overcompensates for a relative loss of redundancy. Optimizing redundancy alone reliably selects the weight configuration which maximizes redundancy, thus necessarily matching or exceeding the redundancy found by other learning rules with additional terms. Why the local loss landscape changes for higher α in such a way as to tilt mutual information maximization to a solution with more unique information, however, remains an open question. While SxPID (based on the $I_{\cap}^{\text{sx}}$ redundancy measure) allows negative values for the unique information terms in principle [2], this does not appear to be relevant to the phenomenon in question.

- Thus, the justification for claiming that maximizing mutual information implicitly maximizes redundancy seems insufficient. Furthermore, it is important to understand what happens at the threshold α_c^{red} that $I(Y : I)$ starts diverging from Π_{red} (although, to me it seems like they should not as per my above comment about the optimization).

In our results, we show that both maximizing mutual information and maximizing redundancy directly lead to high redundancy below capacity. Since removing the unique information from the mutual information goal function does not majorly negatively impact performance, we suggest that it is the redundant contribution which primarily determines task performance.

- In fact, if redundancy maximization is truly sufficient, the capacity should not be affected at all by $\gamma_{unq,T}$ in Figure 3D as we are maximizing the redundancy in all cases. However, the results show that negative values of $\gamma_{unq,T}$ (implying also minimizing $\Pi_{unq,T}$) actually hurts capacity.

As mentioned before, optimizing a goal function containing a positive term for redundancy does not necessarily result in the same maximal redundancy as optimizing for redundancy alone. We see in our experiments that whenever a goal function manages to find a high-redundancy solution, the task performance is likewise high. Therefore, sufficiency of redundancy does not imply that optimizing for any goal function involving redundancy will yield maximum performance. Rather, our claim is the inverse: If a good solution is found, it must have—explicitly or implicitly—optimized for redundancy.

The analysis using hyperparameter optimization for the goal parameters is very interesting. However, the results highlight the lack of interpretability in the results (which is counter to the claims of interpretability in the PID framework). For the optimal hyperparameters in Figure 4C,D, it is odd that $\gamma_{unq,T}$ is both positive and negative, implying that we can either maximize $\Pi_{unq,T}$ or minimize it, which makes the decomposition uninterpretable. This is also counter to the results shown in Figure 3D, which implied that negative $\gamma_{unq,T}$ leads to worse capacity, and increasing it does not affect capacity significantly. Thus, it is unclear how we can square the resuts in Figure 3D with the hyperparameter optimization results in Figure 4C,D.

Our results show that high task performance can indeed be achieved both with positive or negative values for $\gamma_{\text{unq},T}$. This implies that adding or removing unique target information as a goal does not majorly affect the results, matching our hypothesis that the optimization for redundancy is the decisive factor in determining task performance. Upon close inspection, the results shown in Figure 5 (previously Fig. 4) are in agreement with those in Figure 4D (previously Fig. 3D): There, the goal parameter for redundancy is fixed at 1 and the parameters for synergy and recurrent unique are fixed at 0. In Figure 5A, these values can be found as the center points in the rightmost column, which do show decreasing performance for negative contributions of $\gamma_{\text{unq},T}$.

Minor typographical error

- In line 201, I think the authors wish to refer to Figure 5C, not Figure 5B.

We thank the reviewer for pointing this out, we fixed it.

Conclusion

Overall, in this manuscript, the authors put forward an intuitive, though somewhat expected and known as per [13], objective for learning weights in the classical associative memory network. However, the objective itself requires the hard estimation of joint probabilities, and the current manuscript does not discuss how the estimation scheme and quality affects the results in this manuscript. Furthermore, the results are empirical in nature, and are limited to a small number of configurations of associative memory networks, making it unclear whether these results are robust and significant in general.

We thank you for your insightful and detailed feedback. With the additional experiments and clarifications that your comments have inspired, we believe that the strength of our arguments has been significantly improved.

Reviewer 2

In this paper, Blumel et al., use the partial information decomposition to interrogate the dynamics of associative memory in Hopfield networks, finding that Hebbian learning seems to be driven by redundancy, and apparently compromised by unique and synergistic interactions. They then go on to show that an "infomorphic neuron", trained to maximize redundant information (or some linear combination of partial information atoms) can achieve very high performances on an associative learning task - comparable with current state of the art.

In general, I am a big fan of this paper. I think this is an excellent application of PID to a concrete and well-defined problem. I am also very enthusiastic about the use of information-theoretic objective functions. So much of information theory in complex systems stops at the level of descriptive statistics (e.g. neuroscience studies that ask "is there more synergy in an awake brain than an unconscious one"), and it is exciting to see a new spin on these methods.

In general the methodology seems strong and the results robust. I have some small technical quibbles, but they can be addressed with minor revisions.

One thing that gives me pause is that the authors also try to maximize the co-information, and find that no patterns are stored. I cannot find any place in the paper where the definition of co-information is given, so I will assume that here the definition is the same as the original Bell paper [1], which is positive when redundant interactions dominate, and negative when synergistic interactions dominate. Later, Williams and Beer [2] showed that, for two sources X_1, X_2 and one target Y, the co-information is equivalent to Red(12 ; Y) - Syn(1,2 ; Y). Based on this identity, I find it a little concerning that optimizing the co-information apparently does not perform at all, even when the i_{sx} redundancy function does. If it were the case that redundancy generally improves performance, then naively, I would expect optimizing the co-information to perform equivalently well, especially since the goal function $G^{(i)}$ has a very co-information-like form (positively weighting redundancy, negatively weighting synergy).

As it stands, I don't know what to make of this apparent inconsistency. Are the authors sure that the co-information was implemented correctly? The literature on co-information is messy, sometimes co-information is used to refer to the inverse-signed measure, which is Syn(1,2) ; Y) - Red(12 ; Y).

To avoid ambiguity, we have added the formal definition of co-information to our manuscript and confirmed that the definition of co-information used by us indeed corresponds to redundancy minus synergy (see also Fig. 4A depicting the classical co-information goal on the right). We examined the network's learning behavior, varying only the goal parameters for redundancy and synergy (see Figure 4A). The results indicate two distinct regions with non-zero performance, separated by minimal performance exactly at the line of (multiples of) co-information as goal function ($\gamma_{\mathrm{red}} = -\gamma_{\mathrm{syn}}$). Thus, while we can confirm that co-information itself is not a valid goal function, adding even small amounts of redundancy leads to a goal function with intermediate capacity $\alpha_c \approx 0.8$. If a purely classical goal function is desired, adding $I(Y:T)$ or $I(Y:R,T)$ to the goal function also has the same effects. Nevertheless, why only the exact goal of (multiples of) co-information produces lower performance than small perturbations from it remains a striking result warranting a more comprehensive analysis in future work.

> Another small thing is that I'd like to see a cartoon that describes Hopfield networks a little more intuitively. Fig. 1A kind of starts, but it's still a little abstract. Is there a visualization that describes the entire process up updating (including intuitive representations of equations like Eqs. 1 and 2? I think this would go a long way towards making the paper more accessible.

We thank the reviewer for this suggestion. We added a new introductory figure (new Figure 1) that illustrates the retrieval process of a Hopfield network, including stored patterns, the recurrent weight structure, the iterative update dynamics, and the energy landscape perspective. We hope this makes the paper more accessible to readers less familiar with Hopfield networks.

> All in all, I commend the authors for an impressive piece of work.

[1] Bell, A. J. (2003, April). The co-information lattice. 4th International Symposium on Independent Component Analysis and Blind Signal Separation, Nara, Japan. [2] Williams, P. L., & Beer, R. D. (2010). Nonnegative Decomposition of Multivariate Information. arXiv:1004.2515

Reviewer 3

> The authors provide an analysis of Hopfield networks based on partial information decomposition (PID). The authors find empirically that high redundancy is correlated with high recall accuracy in Hopfield networks, but is not strongly correlated with other components used in the PID profile. This is used as motivation to optimize for redundancy. Building on previous work on infomorphic networks, they design a type of Hopfield network that optimizes the informorphic objective, which is composed of a weighted sum of PID components. They run multiple experiments that test various weightings of the infomorphic objective, including hyper-parameter sweeps, and observe that redundancy is the most important component for associative memory recall accuracy. They also show that their objective leads to better accuracy than other SOTA objectives at high memory loads.
> The authors perform an insightful and novel analysis of Hopfield networks based on PID, and they use this analysis to improve performance of auto-associative networks over SOTA methods. The paper is well written, the diagrams provide useful explanations of concepts and experiments, and the experiments are logical.
> Below I provide suggestions for making the paper a bit clearer for those new to PID, and how to strengthen the empirical results and explanation of the results.
> For equations 3 and 4, I believe it would be helpful to have a table, possibly in the appendix, that explicitly defines the Π terms (Π_{syn},Π_{red}, etc.). These are nicely visualized in Figure 1C, but I can't seem to find an explicit mathematical definition in the paper. They can be inferred from equations 3,4, and the diagrams, but an

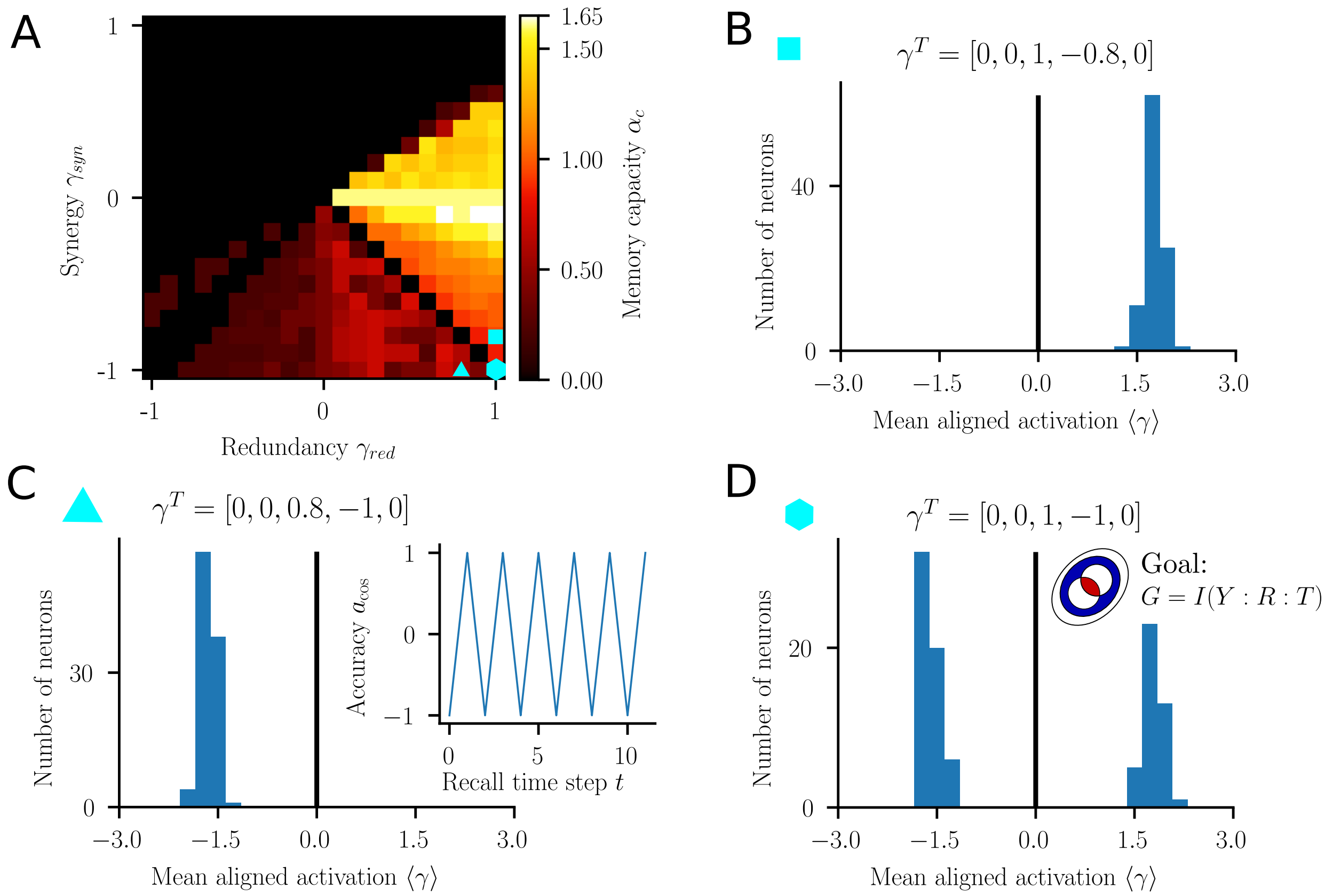

Figure 4: **Co-Information seems to fail as a goal because it is located directly between two regions with qualitatively different solutions. A:** The memory capacity is shown in the goal space as a function of goal strength for redundancy γ_{red} and γ_{syn}. The landscape shows two regions of successful solutions, one centered around redundancy $G = \Pi_{\text{red}}$ and one around negative synergy $G = -\Pi_{\text{syn}}$. Co-information lies on the diagonal exactly separating the two. **B:** The panel shows the frequency of mean alignment $\langle\gamma\rangle = \langle\frac{RT}{|W_i|}\rangle$ between target T and recurrent activation R at each neuron after training primarily on redundancy. The network was trained at a memory load of $\alpha = 0.2$ and all neurons have positive mean alignments. Panels **B-D** have their exact goal marked in cyan in Panel A. **C:** Networks trained primarily on minimizing synergy lead to oscillations between the pattern and its mirror image. The inset shows accuracy as a function of recall time step$t$ for the goal function $G = 0.8\Pi_{\text{red}} - \Pi_{\text{syn}}$. Below the (reduced) memory capacity α_c, accuracy oscillates between 1 and -1 during recall. In accordance with this, the mean alignment $\langle\gamma\rangle$ is negative for all neurons. **D:** When training on co-information, the mean alignments $\gamma = \langle\frac{RT}{|W_i|}\rangle$ split into two. Each neuron shows either a strong positive or negative mean level of activation, thus representing a mix between the solutions seen in the two regions of goal space.

explicit definition would be nice. If these definitions do exist in the paper, could the authors make sure there is a clear reference to it? Otherwise, could the authors spell these definitions out somewhere?

We thank the reviewer for pointing this out. The information atoms are calculated via Moebius inversion of Eq. 7 after defining a measure for redundant information. We have added the explicit calculation of information atoms to Appendix subsection A.1 and reference it in section 4.2 of the main text.

From what I could tell, the authors ran all of their tests from section 3.2 onwards with networks of size 100 neurons, and section 3.1 experiments with networks size 500 neurons. Is this correct? If so, could the authors also run tests on other sized networks? I believe it is important the authors establish that their empirical results generalize to any reasonably sized network. For example, maybe they could test at [50, 100, 200, 400, 800] neurons to see if there are any clear trends as they double the size of the network. Maybe doing this for the tests in figure 2 and figure 5 would be sufficient. Also, showing the findings from section 3.1 generalize to at least one more network size would be useful too. These extra results can then be placed in the appendix and noted in the main text.

This is a very valuable comment. Indeed, we implicitly claim that memory capacity scales linearly with the number of neurons in the network, but neglected to provided evidence for this hypothesis. As requested, we have run experiments for multiple network sizes and added the results to Appendix E (Figure 8), with references from the main text. Both memory capacity α_c and stability $f_{\max}$ are found empirically to scale linearly with network size, up to some minor differences in shape.

Would it be feasible for the authors to test a version of their memory network on continuous valued vectors, e.g., vectors with values between 0 and 1 or between -1 and 1? I suspect this may best be left to future work, but I was curious what the authors' thinking is about this.

This is a very intriguing question, especially against the background of recent successes in continuous-valued Hopfield networks. Unfortunately, our framework would need major adaptations to accommodate for continuous targets. While infomorphic neurons do in fact easily deal with continuous inputs due to the binning procedure (see for example Schneider et al. [3]), their outputs are discrete and currently also binary, because the discrete PID measure requires us to calculate probabilities for each output. This discreteness in the output would inevitably lead to poor reconstruction performance for continuous-valued memory patterns because no matter which pattern was put into the network, all attractors would be $\in \{-1, 1\}^N$. Overcoming the limitation of discrete outputs would require using continuous PID estimators. While continuous PID estimators exist (e.g., [4]), they are typically not differentiable and their introduction would require a major re-work of the current framework for infomorphic neurons, a comprehensive account of which we will have to leave to future work.

The authors provide empirical motivation for optimizing for redundancy for auto-associative memory. However, I think the paper lacks a clear mathematical intuition for why this should be the case. For example, I do not see an attempt in the discussion section to provide mathematical intuition for why, for example, redundancy should be more important than mutual information or other similar measures when training auto-associative memory units. Do the authors believe there is good mathematical intuition for why we see the empirical results we do? Is there reason to believe this empirical result is not somehow unique to the particular experiments ran in the paper (e.g., with binary vectors, at 100 neurons, etc.) and generalizes beyond it? If so, what is this explanation?

We thank the reviewer for raising this important question, which prompted us to substantially strengthen the theoretical foundations of our work. We have added a comprehensive analytical treatment in Appendix F and summarize the core arguments below.

Why redundancy is *necessary* for associative memory. In Appendix F.1, we provide a formal proof that high redundancy is a necessary condition for successful associative memory function, independent of network size or how the weights are obtained. For successful recall under a Heaviside activation, each neuron's recurrent input R must satisfy $\mathrm{sgn}(R) = \mathrm{sgn}(T)$ for almost all samples. Geometrically, the conditional distributions $p(R \mid T = +w_T)$ and

$p(R \mid T = -w_T)$ must be supported on opposite sides of $R = 0$. Starting from the $I_\cap^{\text{sx}}$ definition, we show that whenever these supports are disjoint in this way, the union probabilities collapse and redundancy attains its maximum value $H(Y) = H(T) = 1$ bit. Conversely, *any* overlap across the decision boundary strictly decreases redundancy. Since disjoint supports are precisely the condition for perfect recall, redundancy is provably maximal if and only if associative memory is successful.

Why redundancy as the underlying principle and not mutual information or co-information. The reviewer asks specifically why redundancy should be more important than classical measures. The PID consistency equations give a clean answer: classical information-theoretic quantities can be maximized in two ways—one that increases its redundant component and supports associative memory, and one that increases other components that do not. **Target mutual information** $I(Y : T) = \Pi_{\text{red}} + \Pi_{\text{unq},T}$ can be raised either by increasing Π_{red} (the recurrent input genuinely encoding the target) or by increasing $\Pi_{\text{unq},T}$ (the neuron simply copying the teaching signal). Below capacity, gradient ascent finds the redundancy maximization route, which is why $I(Y : T)$ matches redundancy in performance (Figure 4**C**). Above capacity, however, the shortfall in achievable Π_{red} is compensated by persistent $\Pi_{\text{unq},T}$, keeping $I(Y : T)$ high even as recall degrades. Under $G = \Pi_{\text{red}}$, by contrast, $\Pi_{\text{unq},T}$ does not increase above capacity (Figure 3**D**). **Co-information** $\Pi_{\text{red}} - \Pi_{\text{syn}}$ fails as a goal because it leads to a mix of two qualitatively different solutions (see Figure 4). **Recurrent mutual information** $I(Y : R)$ does not constrain alignment with T and similarly fails.

Generality of the results. We support the generality of our core results with several additions to the appendix:

- Analytical derivation for Hebbian networks (Appendix F.3): A closed-form expression for $I_\cap^{\text{sx}}(Y : R; T)$ as a function of α and N reproduces the empirical profile of Figure 2fig:hebbD (Figure 13**C**), confirming the signature is not an artifact of finite sampling or binning.
- Robustness across PID measures and binning (Appendix F.4, Figure 14–Figure 15): The qualitative profile is preserved under $I_\cap^{\text{BROJA}}$, $I_\cap^{\text{min}}$, I^{PM}, and I^{MMI}, and across a range of bin counts.
- Scaling with network size (Appendix E, Figure 8–Figure 9): Capacity and stability scale linearly with N from 50 to 800 neurons.

We have updated the Discussion to point readers to Appendix F and added cross-references in Sections 2.1 and 2.3 of the revised manuscript.

Reviewer 1

> I would like to first thank the authors for addressing all my comments in a thorough professional manner. I really enjoyed reading the updated version and the author responses. I appreciate the detailed clarifications to my various questions regarding the effect of the different weighted sum objective for infomorphic learning and how to appropriately interpret the results. The new results covering the scaling of the number of memories (Appendix E), the analysis of the training runtimes (Figure 20), and the effect (or rather lack thereof) of changing the PID term estimation schemes and parameters (Appendix F.4) clearly highlight the robustness of the trends initially seen on smaller experiments.
> I also appreciate the proper positioning with respect to Makkeh et al [13], highlighting the role of redundancy maximization in associative memory learning. The new analysis in F.1 provides a concrete connection with the geometric separation (necessary for recall in Hopfield models) and the maximal redundancy achieved when the separation holds. Analysis in F.3 studies the Hebbian learning, showing how the available gap between the signal and the noise terms (at different memory loads) translates to the separation condition, which in turn leads to insights regarding the redundancy (loads when it is almost maximal vs loads where redundancy starts to drop, matching the existing empirical evaluations). In my opinion, these results provide a more thorough connection between redundancy maximization and associative memory learning.

We want to thank the reviewer for this positive assessment, and for their earlier comments which helped us to improve our paper.

> I also appreciate the ablation experiments in Appendix H demonstrating the different additions on top of Makkeh et al [13], and how it affects capacity. However, the results are a bit counterintuitive. Based on Figure 17, it appears that the major capacity jumps come from (i) deterministic testing for (5x) longer number of recall time steps, (ii) modifying the frozen w_T value from 1.0 to 2.3, and (iii) switching the optimizer from SGD to Adam. Jump (i) implies that one just needs to take the model learned from Makkeh et al [13] and iterate it for longer recall timesteps with appropriate nonlinear activation (as in the standard Hopfield model). The jump (ii) indicates, somewhat counterintuitively, that freezing the w_T parameters (instead of learning it from data) and manually setting it to 2.3 is much better than trying to learn the w_T value. Jump (iii) indicates that we had the right learning objective, but we were not optimizing it appropriately (or correspondingly, the SGD hyperparameters might not have been sufficiently tuned). All these are interesting additions, but appear more like engineering steps, and do not seem to align with the main contributions of this submission on top of Makkeh et al [13] such as the role of redundancy and the weight objective. It is possible that I am misunderstanding something, but I find these ablation results separating this current submission from prior art a bit limiting.

We agree with the reviewer's summary of the additions since Makkeh et al., and that these are not major conceptual changes to the training procedure. Appendix H (Supplementary Note 8) was not intended as a primary contribution. Its purpose was to improve reproducibility by documenting the changes needed to reach the reported capacities. We also agree that the resulting improvements in capacity relative to other learning rules (Sec. 2.5) are a contribution distinct from our analysis of Hebbian learning (including App. F / SN 6, added during review).

In our view, however, these improvements align with the paper's claims in a broader sense, because they are what enable the more comprehensive tests of robustness—both with respect to input noise (Sec. 2.5) and to different goal functions (Sec. 2.4). Together these empirically test the generality of redundancy learning and support the theoretical arguments given earlier in the text. The central claim itself, namely that maximizing local redundancy between recurrent and target

inputs is necessary and sufficient for associative memory learning, rests on the results in the main text. It is further supported by App. F / SN 6 and is independent of the specific choice of optimizer, recall length, or w_T.

> Finally, this w_T parameter appears to be a critical hyperparameter in terms of capacity. Thus it is odd to see coarse-grain evaluation of this parameter for hebbian learning in the set $w_T \in 10^{-10}, 1, 10, 100$ in Figure 18, whereas a much more fine-grained values around [0,10] is considered for infomorphic learning in Figure 19. If the coarse-grain grid (from Hebbian learning) was used, the whole range of [2,5] where memory capacity is above 1 is completely lost. It is also not clear why learning the w_T is so detrimental; it appears better than Hebbian but quite a drastic drop from the fixed w_T setting. Also, given the scaling experiments in Appendix E, the interaction of this w_T and the number of neurons and memories is not well understood. This critical role of a somewhat underdiscussed hyperparameter undercuts the main contribution of this submission.

The reason for the different ranges of w_T reflect their fundamentally different roles across the two figures.

In the Hebbian setting, T is purely used as an auxiliary variable to analyze the PID between R and T about Y and not for learning, as it never enters the Hebbian weight update rule. In the Hebbian Hopfield network, the parameter w_T can only have a significant impact on the network's dynamics by being extremely high and thereby allowing an active influence of T on the output. The comparison of the results for four different values of $w_T \in 10^{-10}, 1, 10, 100$ is thus more of a robustness check on the PID computation itself. The goal of the comparison is not to find a capacity optimum as the Hebbian recall is fully determined by the weights defined by the outer product of the patterns.

In contrast, in the infomorphic setting w_T is a hyperparameter used during the training phase of the networks that directly influences the neuron's learning and therefore also affects the memory capacity. This is the reason for a more fine grained comparison of the capacity.

However, your criticism regarding the role of the parameter itself is very important and we added a sentence to the limitations discussion noting that a systematic understanding of w_T and especially its interaction with network size remains an open question.

Reviewer 2

> The paper can be published as-is. I commend the team for an excellent piece of work.

We would like to thank the reviewer for their positive and encouraging feedback.

References

[1] Abdullah Makkeh, Marcel Graetz, Andreas C. Schneider, David A. Ehrlich, Viola Priesemann, and Michael Wibral. A general framework for interpretable neural learning based on local information-theoretic goal functions. *Proceedings of the National Academy of Sciences*, 122(10):e2408125122, 2025. doi:10.1073/pnas.2408125122. URL `https://www.pnas.org/doi/abs/10.1073/pnas.2408125122`.

[2] Abdullah Makkeh, Aaron J Gutknecht, and Michael Wibral. Introducing a differentiable measure of pointwise shared information. *Physical Review E*, 103(3):032149, 2021.

[3] Andreas C. Schneider, Valentin Neuhaus, David A. Ehrlich, Abdullah Makkeh, Alexander S. Ecker, Viola Priesemann, and Michael Wibral. What should a neuron aim for? Designing local objective functions based on information theory. In *The Thirteenth International Conference on Learning Representations*, 2025. URL `https://openreview.net/forum?id=CLE09ESvul`.

[4] David A Ehrlich, Kyle Schick-Poland, Abdullah Makkeh, Felix Lanfermann, Patricia Wollstadt, and Michael Wibral. Partial information decomposition for continuous variables based on shared exclusions: Analytical formulation and estimation. *Physical Review E*, 110(1):014115, 2024.
